# Linear Superposition and Prediction of Bacterial Promoter Activity Dynamics in Complex Conditions

**DOI:** 10.1371/journal.pcbi.1003602

**Published:** 2014-05-08

**Authors:** Daphna Rothschild, Erez Dekel, Jean Hausser, Anat Bren, Guy Aidelberg, Pablo Szekely, Uri Alon

**Affiliations:** Department of Molecular Cell Biology, Weizmann Institute of Science, Rehovot, Israel; Princeton University, United States of America

## Abstract

Bacteria often face complex environments. We asked how gene expression in complex conditions relates to expression in simpler conditions. To address this, we obtained accurate promoter activity dynamical measurements on 94 genes in *E. coli* in environments made up of all possible combinations of four nutrients and stresses. We find that the dynamics across conditions is well described by two principal component curves specific to each promoter. As a result, the promoter activity dynamics in a combination of conditions is a weighted average of the dynamics in each condition alone. The weights tend to sum up to approximately one. This weighted-average property, called linear superposition, allows predicting the promoter activity dynamics in a combination of conditions based on measurements of pairs of conditions. If these findings apply more generally, they can vastly reduce the number of experiments needed to understand how *E. coli* responds to the combinatorially huge space of possible environments.

## Author Summary

Bacteria face complex conditions in important settings such as our body and in biotechnological applications such as biofuel production. Understanding how bacteria respond to complex conditions is a hard problem: the number of conditions that need to be tested grows exponentially with the number of nutrients, stresses and other factors that make up the environment. To overcome this exponential explosion, we present an approach that allows computing the dynamics of gene expression in a complex condition based on measurements in simple conditions. This is based on the main discovery in this paper: using accurate promoter activity measurements, we find that promoter activity dynamics in a cocktail of media is a weighted average of the dynamics in each medium alone. The weights in the average are constant across time, and can be used to predict the dynamics in arbitrary cocktails based only on measurements on pairs of conditions. Thus, dynamics in complex conditions is, for the vast majority of genes, much simpler than it might have been; this simplicity allows new mathematical formula for accurate prediction in new conditions.

## Introduction

Bacteria respond to their environment by regulating gene expression [1]–[5]. Gene expression is determined by global factors such as the cell's growth rate and overall transcription and translation capacity [6]–[10], together with specific factors such as transcription regulators that respond to specific signals.

The environments that bacteria encounter are often complex, made up of combinations of many biochemical components and physical parameters. For example, natural habitats of bacteria include the soil [11], [12] and the human gut [13]–[15]. Complex conditions are also of interest in applications such as food science and bioenergy [16]–[20]. It is therefore of interest to understand how cells respond to complex conditions. However, experimental tests run up against a combinatorial explosion problem: in order to test all combinations of N factors, one needs 2^N^ experiments. For example, a food scientist that seeks to test bacterial gene expression in all possible cocktails of 20 ingredients at two possible doses needs more than a million experiments, 2^20^ = 1,048,576 experiments. If four doses are considered, 4^20^∼10^12^ experiments are needed. Important recent advances on bacterial gene expression made by Gerosa et al [7] and Keren et al [10] do not overcome this concern, because one still needs to measure expression in each combination of conditions. Thus, the search for simplifying principles is important.

One such simplifying principle was suggested in a study of the protein dynamics in human cancer cells in response to drug cocktails [21]. Protein dynamics in a drug combination were well described by weighted averages of the dynamics in the individual drugs. This feature was termed linear superposition (also known as convex combination or weighted average). Furthermore, it was found that measuring dynamics in drug pairs could be used to predict the dynamics in drug triplets and quadruplets. This opens a possibility for avoiding the combinatorial explosion problem: To predict gene expression in all possible combinations of N drugs it is sufficient to measure all N(N-1)/2 pairwise combinations instead of 2^N^. For example, the response to all combinations of 20 drugs can be well approximated by measurement of the 190 pairwise combinations, rather than over a million combinations. The number of necessary experiments is reduced by more than 5000 fold.

Here, we asked whether the linear superposition principle might apply also to understanding the response of *E. coli* to combinations of growth conditions. Since we consider the transcriptional response of bacteria to natural stress conditions, rather than the proteomic response of cancer cells to anti-cancer drugs, this study explores this principle in a very different biological context. We used a promoter library to obtain accurate dynamics of 94 promoters as bacteria grew from exponential to stationary phase in all possible combinations of a set of nutrients and stresses. We find that dynamics in a mixture of conditions is, for most genes and conditions, well described as a linear combination – a weighted average – of the dynamics in the individual condition. The weights sum up to approximately one. We also found that part of the reason for this feature is that promoter activity dynamics for each gene seem to be quite limited, and are explained effectively by one or two principal components. Using linear superposition, we employ mathematical formulae that allow predicting the dynamics in cocktails of conditions based on measuring pairs of conditions. This suggests that the combinatorial explosion problem may be circumvented, to understand and predict bacterial responses to complex conditions.

## Results

### Promoter activity dynamics in combinatorial conditions was measured using an *E. coli* reporter library

We studied 94 genes and 2 control strains (see Materials and methods), in a 96 well plate format. We chose 94 genes which represent a wide range of biological functions (Table S1), and which have a strong detectable fluorescence signal in a range of growth conditions (more than 2 standard deviation above background).

**Figure 1:**
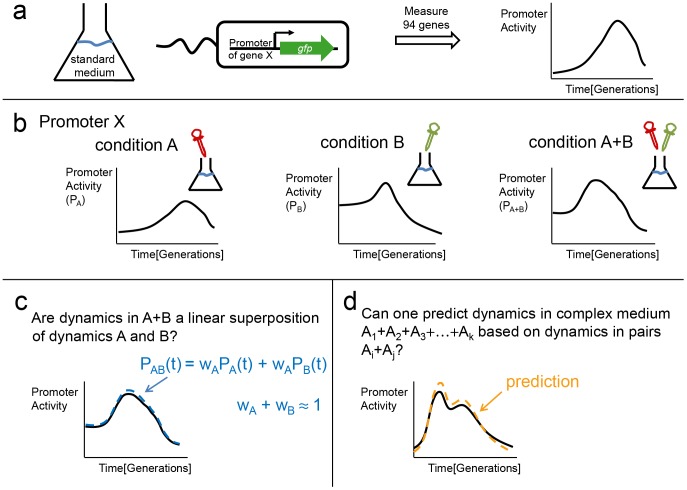
Schematic overview of workflow for measuring promoter activity dynamics and the analysis testing linear superposition of dynamics in different conditions. (a) *E. coli* reporter strains were grown in defined media conditions in 96-well plates and promoter activity – the rate of GFP accumulation per cell – was found as a function of time. (b) Promoter activity dynamics was measured in four conditions and all of their possible pair, triplet and quadruplet combinations. (c) We tested whether the dynamics in a combined condition is a linear combination (weighted average) of the dynamics in each individual condition. We further asked whether the weights sum up to one, signifying a linear superposition. (d) Finally, we asked whether dynamics in triplet and quadruplet conditions can be predicted based on dynamics in pairs of conditions.

We measured promoter activity of these genes as a function of time using the *E. coli* reporter library developed in our lab [22] (Figure 1a). Each reporter strain had a rapidly maturing GFP variant (gfpmut2) under control of a full length intragenic region containing the promoter for the gene of interest, on a low copy plasmid (Figure 1a). Promoter activity was measured as the time derivative of GFP fluorescence accumulation divided by cell density, as described [23]–[25] (Methods). Using this approach, the temporal dynamics of promoter activity can be measured at high accuracy [26]–[28].

We aimed at understanding the promoter activity dynamics in growth media composed of combinations of chemical conditions. For this purpose we chose 4 elementary conditions. Each condition is based on a chemically defined medium, M9+0.2% glucose as the carbon source. In each elementary condition one supplement is added (A) 0.05% casamino acids, (B) 3% ethanol, (C) 10 µM hydrogen peroxide H_2_O_2_ (D) 300 mM NaCl salt. In all four conditions, cells reached a similar final optical density (OD), with different growth rates (Table S2).

We studied combinations of these conditions by mixing the appropriate supplements into the standard medium. Thus, condition A+B is standard medium supplemented with 0.05% casamino acids and 3% ethanol (Figure 1b). In total, we studied all four single conditions, all six pairs, all four triplets and the quadruplet A+B+C+D (The different growth rates of all combinations is given in table S2).

In each condition, we measured promoter activity of the 94 genes at an 8 minute resolution, throughout batch culture growth, including exponential growth phase and stationary phase. Depending on the growth rate in a given condition the stationary phase was reached after 8 to 22 hours of growth. Each experiment was repeated on four different days.

### Promoter activity dynamics across conditions is described by one or two principal components

**Figure 2:**
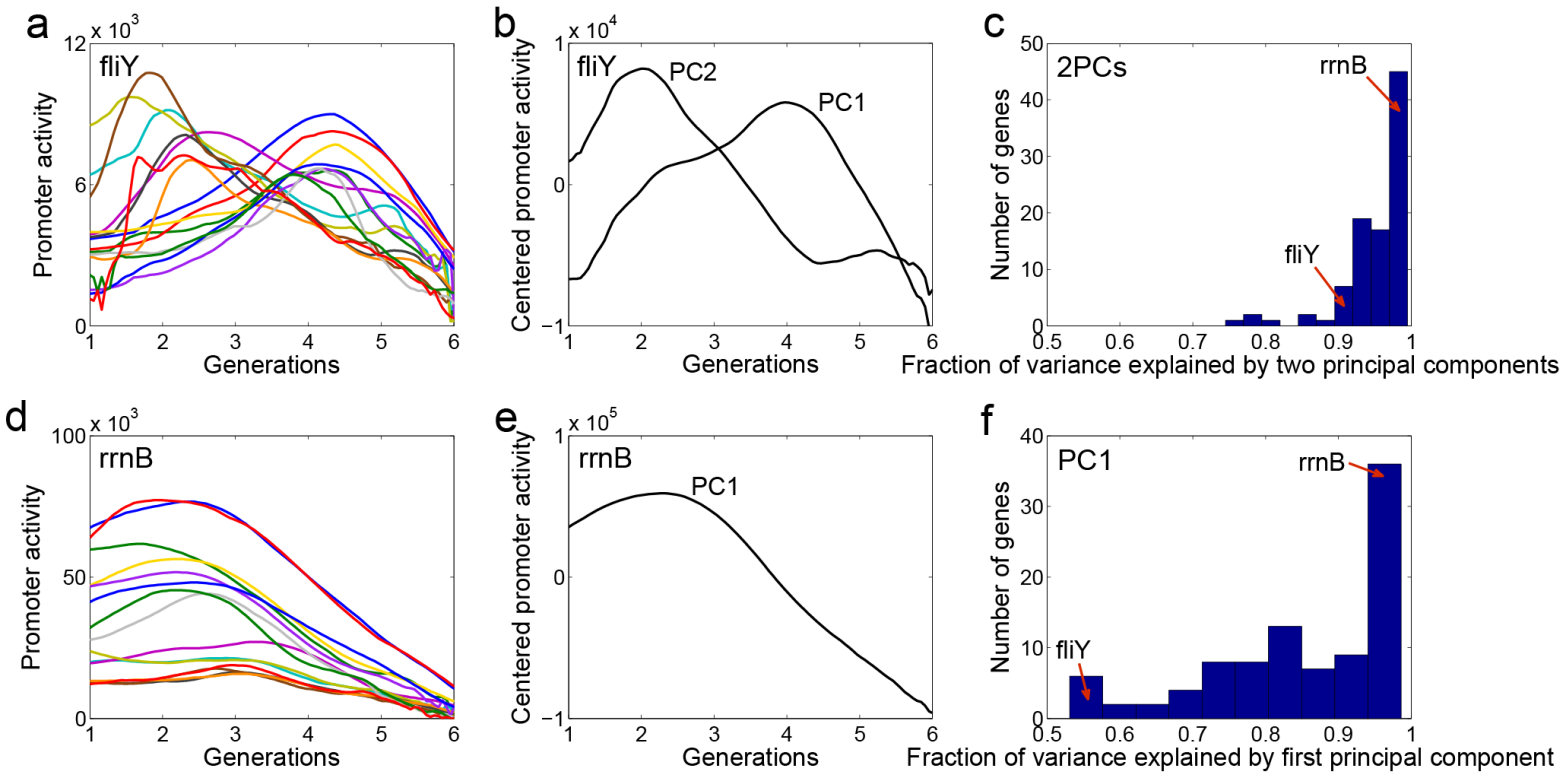
Promoter activities of genes can be well-described by one or two principal components. (a) *fliY* promoter activity dynamics in 15 different measured conditions (15 combinations of conditions A,B,C,D). (b) First two principal components dynamics of *fliY*, according to principal component analysis of *fliY* dynamics in all conditions and combinations. (c) Fraction of variance explained by the first two principal components for all 94 promoters in 15 environments. Red arrows: *fliY* and rrnB with 91% and 99% explained variance. (d) *rrnB* promoter activity dynamics in 15 different measured conditions (e) The first PC of *rrnB* according to principal component analysis of *rrnB* expression dynamics in all conditions and combinations. (f) Fraction of variance explained by only the first principal components for all 94 promoters in 15 environments. Red arrows *fliY* and rrnB with 53% and 98% explained variance.

We observed that promoter activity dynamics of a given promoter can vary both in shape and in amplitude across different growth conditions. Using principal component analysis we can identify the typical shapes of every promoter across conditions. In figure 2 we show the activity dynamics of *fliY* in all measured conditions (Figure 2a) and its two principal dynamic curves PC1 and PC2 (Figure 2b). We found that each promoter can be well described by two principal component dynamic curves, which explain 80–99% of its variance (Figure 2c). In more than 93% of the promoters, the two first PCs explain 90% or more of the variance (Figure 2c). Because of the 2PC property, each promoter activity curve is a linear combination of its two PCs to a good approximation. The first two PCs explain much more variance than expected in randomized data (See Figure 1 in Text S1).

About one third (30/94) of the promoters were well explained by one principal component in all measured conditions (Figure 2f). The dynamics of these promoters thus had a rather constant shape in different conditions, and differed only in amplitude. For example one PC explains 98% of the variance in the σ^70^ activated ribosomal promoter *rrnB* (Figure 2d,e). The other 2/3 of the promoters, explained well by 2PCs, showed condition-dependent shape changes in their dynamics. The low number of principal component curves needed in order to explain the promoter activity dynamics could be a result of general nonspecific transcription for promoters with only one PC (with only change in amplitude with different growth rates), and could be condition dependent yet limited in number for promoters with two principal component curves.

We find that for 76% of promoter activities, the first PC is highly correlated (R^2^ above 0.8) with instantaneous growth rate (See Figure 6a,b,c in Text S1). This may relate to a principal component analysis by Bollenbach et al [29] that instead of considering dynamics, considered a single point at exponential growth in response to antibiotic combinations. The first PC correlated with growth rate and the second with drug specific effects. The second PC in our dataset varies more widely in shape between different promoters (See Figure 6d in Text S1).

### Dynamics in a combination of conditions is well-described by a linear superposition of dynamics in the individual conditions

We now use the 2PC property to understand how promoter activity dynamics in a mixed condition P_A+B_ relate to the dynamics in each supplement alone, P_A_ and P_B_. Since a promoter can be described as a linear combination of the same 2PCs in any condition, we expect the combined P_A+B_ to be a combination of the one-supplement conditions P_A_ and P_B_:
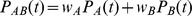



Where the best fit weights are w_A_ and w_B_. To find the best fit weights we aligned the dynamics in conditions A, B and A+B according to a shared axis of generations (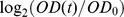 – see Materials and methods and Text S1 Extended methods). Using a generation axis helped compare conditions despite variations in growth rate. We performed linear regression of P_AB_ based on P_A_ and P_B_. These weights are constant over time. Similarly, dynamics in three and four supplements can be represented as linear combinations of the one supplement dynamic:
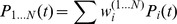



We determined the best fit weights w_i_^(1…N)^ using an error-in-variables linear regression [30] (where w_i_^(1…N)^ is the weight contributed by condition i which best fits the combined condition 1…N – see Text S1). To measure how similar a linear combination is to the measured combination dynamics we compute the relative fit error between the two (Text S1). Linear combination describes the dynamics well (relative error 10% see Text S1), as expected.

So far, these findings are consistent with the 2PC finding. However, we find information beyond the 2PC property, when we examine the sum of the weights in these equations. We find that the sum of weights w_A_+w_B_ in each fit is distributed around one (See Figure 2 in Text S1), with a standard deviation of 0.6. The weights are usually positive (76%>−0.05). This means that the linear combination is approximately a weighted average (see also [29]).The same applies to three and four supplement mixtures. We therefore tested a simpler model, named linear superposition, in which the weights are constrained to sum to one, and be positive:
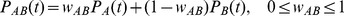



Here the dynamics in the mixture conditions is a linear combination of the individual supplement conditions but with only one free parameter w_AB_, with the constraint that w_AB_ ranges between 0 to 1.

**Figure 3:**
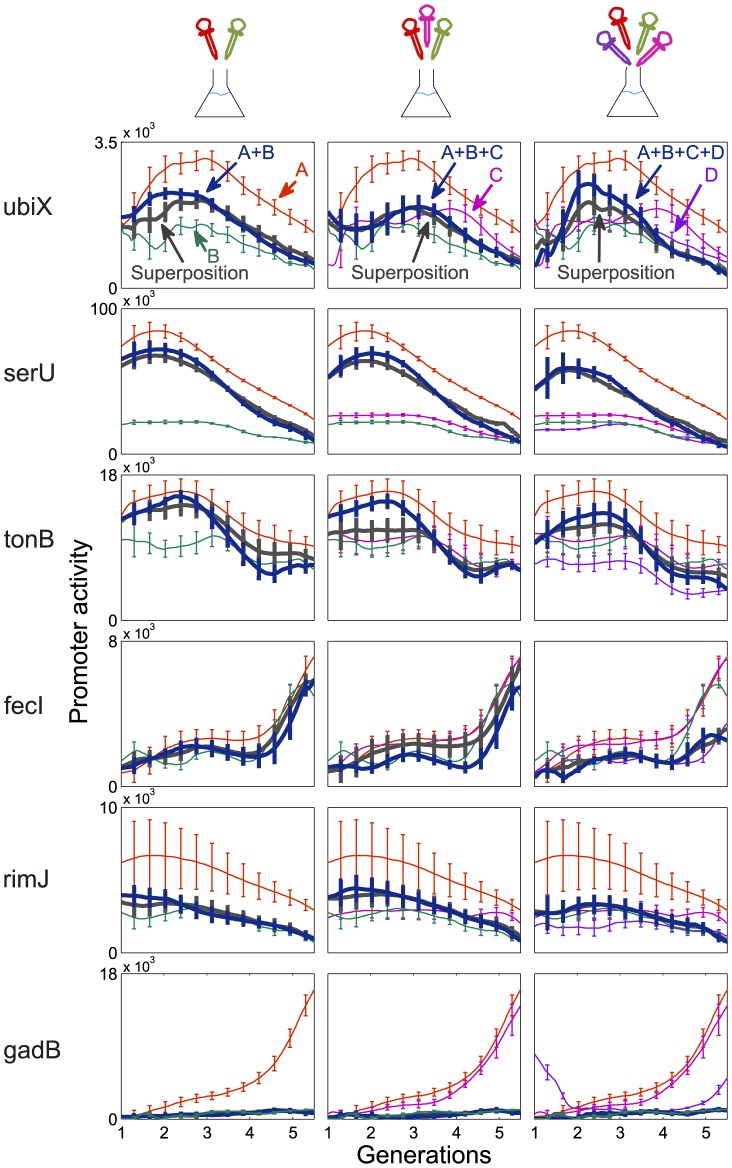
Promoter activity dynamics in combined conditions is diverse and well-described as a linear superposition of dynamics in individual conditions. Six representative promoters are shown (each row belongs to one promoter. The Promoter name is indicated on the left). First column shows individual and pair conditions A+B, second column shows a triplet condition (A+B+C), and the third column a quadruplet (A+B+C+D). A = 0.05% casamino acids, B = 3% Ethanol, C = 10 µM H_2_O_2_, D = 300 mM NaCl, all added to M9+0.2% glucose defined medium. Dynamics in the combined condition (blue curve) are well-described by the best fit linear superposition of individual condition dynamics (black curve). Error bars are standard error between 4 independent experiments on different days.

In most conditions, the linear superposition model gives a better score in describing the data in tests that take model simplicity into account (Akaike information criterion [31], which sums the log likelihood of the model fit and the number of model parameters, see Text S1). The linear superposition model also gave better predictions than a multiplicative superposition model (in which 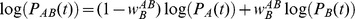, See Text S1). A representative sample of promoter dynamics and the corresponding linear superposition model is shown in figure 3. The mean fit error is 12%, with 86% showing less than 20% fit error. This compares well with the day-to-day experimental error estimated from 4 day-to-day repeats, with average error of 14% (See Figure 3 in Text S1). A table with the weights and errors for all promoters and conditions is provided in the SI (Table S3).

### The linear superposition model does not fit the dynamics of the *lacZ* gene in diauxie conditions

We also sought conditions where linear superposition does not apply. We found one such condition using the classic diauxic shift experiment [32]–[34]. In this case, bacteria grow on a combination of two sugars, glucose and lactose. They begin to utilize the preferred sugar, glucose, and only when glucose is depleted switch to using the second sugar, lactose. The cells thus delay the production of the lactose utilization system – the *lacZ* promoter – until glucose concentration becomes low[23]. Then, cells switch to growth on lactose and express *lacZ* vigorously.

**Figure 4:**
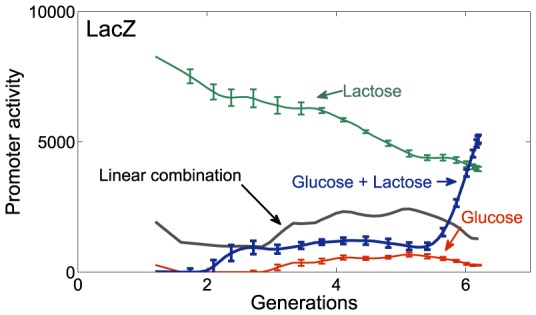
An example of deviation from linear combination is found in the *LacZ* promoter in a diauxic shift experiment. Promoter activity dynamics in a mixture of 0.04% glucose and 0.4% lactose (Blue line) is far from the best fit linear combination of dynamics of glucose or lactose alone (Black line). Error bars are standard error between three independent experiments on different days.

Considering glucose and lactose as conditions X and Y, one does not find that *lacZ* is a linear combination in the combined condition X+Y. This is because under glucose alone, *lacZ* is weakly expressed (Figure 4), and under lactose alone it is strongly and constantly expressed (Figure 4). Linear combination would mean a constant expression at some intermediate value. In contrast, in X+Y, *lacZ* expression is strongly time dependent (Figure 4). Such an effect is expected whenever two conditions interact to regulate genes sequentially [17], [35], rather than simultaneously. Another example we found is the metabolic operon *nudC*, which showed behavior similar to lacZ, and a poor fit to linear combination (See Figure 4 in Text S1). A table with the weights and errors for all promoters in the diauxic shift is provided in the SI (Table S4).

We note that all of the other 92 genes in our study showed good linear superposition in the diauxie condition. This suggests that linear combination might break down for specific genes where the conditions have a nonlinear, sequential effect or more generally distinct temporal dependence on their dynamics.

### Using linear superposition, dynamics in triplets and quadruplets can be predicted based on pairs of conditions

We now use linear superposition to predict the dynamics in a combination of conditions given only data on individual-supplement dynamics, and data on pairs (that is, given the weights w_i_^(ij)^ in pair conditions). Previous work by Wood et al [36], based on a different approach, successfully predicted the growth-inhibitory effect of antibiotic cocktails based on measurement of pairs of drugs. Such predictions are potentially useful because, as discussed in the introduction, it is much easier to measure all pairs than to measure all possible cocktails of N conditions.

The predictions rely on the assumption of linear superposition, specifically that weights sum to one. We apply the formula developed by Geva-Zatrosky et al [21] for predicting protein dynamics in cancer drug cocktails. The formula uses the fact that a combination, say A+B+C, can be treated in three different ways: a mixture of A+B and C, and equivalently as a mixture of A+C and B, and as a mixture of B+C and A. Each of these three possibilities can be described using superposition, and should yield the same result. This provides enough equations to predict the weights needed to calculate the triplet dynamics (See Text S1).

The formula predicts the linear superposition weights in an N-supplement cocktail
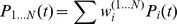



The prediction for the weights w_i_^(1…N)^ based on measurements of the weights in all cocktails of N-1 supplements is [21]:
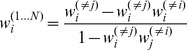
where the superscript (≠j) relates to which supplement is missing in the N-1 cocktail. When only pair data is available, this formula is used iteratively: the triplets are predicted from pair weights, the quadruplet uses these predictions for the triplets weights and so on.

**Figure 5:**
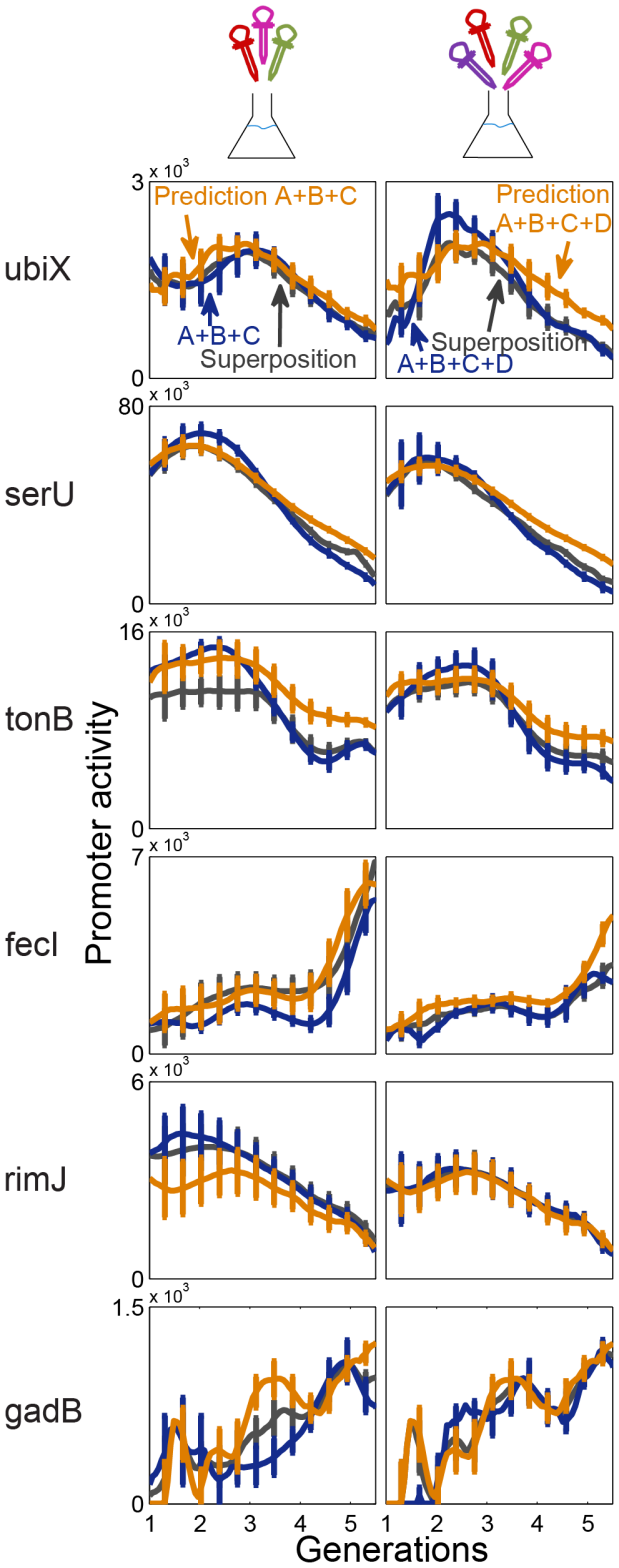
Dynamics in triplets and quadruplet is well-predicted by a formula that employs dynamics in pairs. Right column - prediction of triplet A+B+C (combination of casamino acids, ethanol and H_2_O_2_) – in orange line – follows the measured shape of the dynamics – blue curve. Shown are six representative promoters. The black curve is the best fit linear combination. Left column - same for the quadruplet A+B+C+D (combination of casamino acids, ethanol, H_2_O_2_ and NaCl). Error bars are standard error between 4 independent experiments on different days.

**Figure 6:**
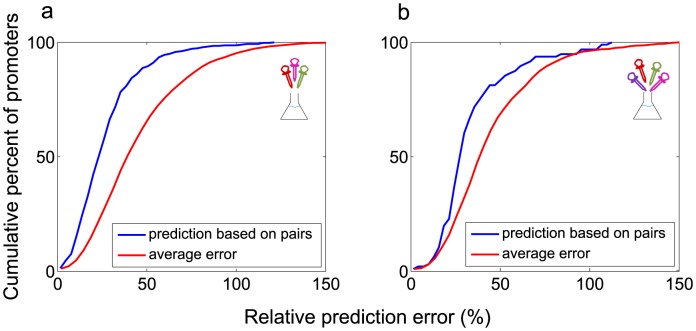
The prediction of dynamics in complex conditions based on pairs of conditions has an error that is smaller than the average expected error. (a) Cumulative histogram of prediction errors of all 4 triplet combinations (in blue) and of the average errors of all other measured conditions (in red) (b) same for the quadruplet.

Using this equation, with pair data only, we find good predictions for the promoter dynamics. Representative dynamics and predictions are shown in figure 5. The median relative error between prediction and measurement is 27% for triplets and 34% for the quadruplet (See Figure 5 in Text S1). These prediction errors are about 2 times larger than the day-to-day experimental error. To evaluate the predictive power of this formula we compared it to what one could expect given no additional information. For this purpose, we ‘predicted’ the dynamics for a given promoter in condition X by randomly picking an exemplar from the available set of measured curves for that promoter in all conditions except X. We then averaged the error between these ‘predictions’ and the measurement in condition X. For example, for a given promoter in condition A+B+C, we used the measured curves in all 14 conditions except A+B+C, namely the 4 single conditions (A,B,C,D), 6 pairs, 3 triplets after excluding A+B+C and one quadruplet. We generated 14 errors and compared the average error to the present formula prediction error. Our formula predictions show about 2.3 times less error than the average error for triplet conditions and about 1.5 times less error in the quadruplet condition (Figure 6).

## Discussion

We studied promoter activity dynamics in combinations of conditions by means of fluorescent reporters. We find that almost all promoters and conditions tested show a linear combination property: the dynamics in a combined condition is a linear combination of the dynamics in individual conditions. The weights in the combination tend to sum to one, and thus combinations act as weighted averages of individual conditions, a property called linear superposition. Linear superposition allowed us to predict the dynamics in triplets and quadruplet based on the dynamics in pairs of conditions. This prediction formula offers a way to reduce the combinatorial complexity of understanding complex conditions.

Genes regulated by specific signals that are strongly time dependent in the complex environment, such as *lacZ* in a diauxic shift experiment (Figure 4), may not display the linear superposition principle. Note that in the diauxie condition, 92 of the 93 other promoters did show linear superposition with good accuracy.

Almost all promoters in this study needed only two principal components to explain their dynamic curves across conditions. This finding is in line with studies on gene expression in a range of organisms [37]–[41]. About one third of the promoters did not show an environmental specific change in the shape of their dynamics and were well explained by only one principal component (Figure 2d–f). It would be interesting to extend this study to investigate the biological meaning of these principal components. It seems that the first PC captures general effects related to the growth [29] (See Figure 6 in Text S1), and the second captures the way that the specific regulation of the promoter changes its first PC dynamics.

The fact that two PCs explain the data well means that promoter activity in a mixed condition can be described as a linear combination of the promoter dynamics in the basic conditions. A further finding is that the sum of weights in this combination is distributed around one. A model of linear superposition, in which weights are constrained to be positive and sum to one, explain the data very well in most conditions. This feature- sum of weights equals one- is crucial to allow predictions of higher order combinations. If the sum of weights was not constrained, one would not have enough equations to predict the weights in a cocktail.

The linear superposition property calls for a biological explanation. One possible framework is the recently suggested finding that when cells compromise between a few tasks, their optimal solution is a gene expression profile that is a weighted average of the optimal profiles for each individual task [42]–[44]. Testing this theory, which is based on a multi-objective compromise between several tasks [45], also known as Pareto optimality, would require understanding the tasks of the cells under the present conditions. Pareto theory points to one possible reason why linear combination might be optimal, which applies in the limit of strong selection under environments which include many combinations of conditions. How linear summation is achieved is a mechanistic question which needs further research. One way that a linear summation can be achieved is when regulatory factors compete over a limiting component - for example: σ^70^ and σ^S^ compete over the RNA polymerase, such that the fraction of σ^70^-RNApol is equal to 1 minus the fraction of σ^S^-RNApol (here we neglected other σ factors). Therefore, the fraction of transcription allocated to growth (σ^70^) and survival (σ^S^) genes follows a line in gene expression space [43]. The position on the line is determined by the ratio of the two σ factor concentrations.

It would be interesting to extend this study to other genes, conditions and organisms. It would be important to find conditions where superposition breaks down, as for *lacZ* in the diauxie conditions described here, to find the limitations of this approach. This approach can be tested also in other levels of cell response, for example one may ask whether linear superposition applies to dynamics of metabolite fluxes [45], [46]. It would be interesting to extend this analysis to situations in which cells show all-or-none patterns of gene expression [35], [47]–[49], and to enhance our understanding of how bacteria compute [50]. If the present approach for predicting dynamics in complex conditions applies more generally, one may attempt to computationally navigate the combinatorial huge space of possible environments, to search for growth conditions with desired gene expression profiles.

## Materials and Methods

### Growth mediums

All media were based on M9 defined medium (42 mM Na_2_HPO_4_, 22 mM KH_2_PO_4_, 8.5 mM NaCl, 18.7 mM NH_4_Cl, 2 mM MgSO_4_, 0.1 mM CaCl). The media used in this study are: Casamino acids (M9 minimal medium, 0.2% glucose, 0.05% Casamino acids); NaCl (M9 minimal medium, 0.2% glucose, 300 mn NaCl); H_2_O_2_ (M9 minimal medium, 0.2% glucose, 10 µM H_2_O_2_); Ethanol (M9 minimal medium, 0.2% glucose, 3% ethanol); and all 15 combinations: 4 single conditions, 6 pairs, 4 triplets and one quadruplet. For example Casamino acids+NaCl (M9 minimal medium, 0.2% glucose, 0.05% Casamino acids, 300 mn NaCl). In addition we measured glucose alone (M9 minimal medium,0.2% glucose); Casamino acids with no glucose (M9 minimal medium,0.05% Casamino acids); Low concentration glucose (M9 minimal medium,0.04% glucose); Lactose (M9 minimal medium,0.4% lactose); Lactose with low concentration glucose (M9 minimal medium,0.4% lactose, 0.04% glucose). In each experiment the bacteria were cultivated in the presence 50 µg kanamycin/ml.

### Robotic assay for genome-wide promoter activity

GFP levels were measured over time for 96 reporter strains (Table S1), each bearing a green fluorescent protein gene (GFP) optimized for bacteria (gfpmut2) on a low copy plasmid (pSC101 origin). All strains in this study were derivatives of wild type *E. coli* K12 strain MG1655. Reporter strains were inoculated from frozen stocks and grown over-night on M9 with 0.2% glucose and 0.05% casamino acids for 16 hours in 600 µl high-brim 96-well plate and reached a final OD of ∼0.9. The 96-well plate was covered with breathable sealing films (Excel Scientific Inc.). The 96-well plates were prepared using a robotic liquid handler (FreedomEvo, Tecan Inc). Overnight cultures were diluted 1∶500 into the micro 96-well experiments plates. The final volume of the cultures in each well was 150 µl. A 100 µl layer of mineral oil (Sigma) was added on top to avoid evaporation and contamination, a step which we previously found not to significantly affect growth [25], [28]. Cells were grown in an automated incubator with shaking (6 hz) at 37°C. A robotic arm moved the micro 96-well plates from the incubator-shaker to the plate reader (Infinite F200, Tecan Inc.) and back. Optical density (600 nm) and fluorescence (535 nm) were thus measured periodically at intervals of ∼8 minutes until reaching stationary phase with a final OD of ∼0.15. Since the overnight cultures on high-brim 96-well plate reached a higher final OD equivalent to about 3 extra generations beyond the micro 96-well plates we obtain data for ∼6 generations of growth.

### Data analysis

Data was obtained from the plate reader software (Evoware, Tecan) and processed using custom Matlab software. Background fluorescence was subtracted from GFP measurements using a reporter strain bearing promoterless vector U139 for each well. Then, promoter activity was calculated using temporal derivative of GFP computed by finding the slope of a sliding window of 17 data points of GFP fluorescence using regression, divided by the mean OD over this window. Varying window size between 5 and 30 affects curve smoothness but does not change the conclusions of this study.

## Supporting Information

Table S1 A gene table with biological description of all promoters.
(XLSX)

Table S2 A table with the dynamics growth rate for all A,B,C,D condition combinations.
(XLS)

Table S3 A table with the weights of the linear superposition and errors for all promoters and A,B,C,D condition combinations.
(XLS)

Table S4 A table with the weights of the linear combination and errors for all promoters in the diauxic shift.
(XLS)

Text S1 Supporting information methods and figures.
(DOC)
