# Supplementary material for: Linear Superposition and Prediction of Bacterial Promoter Activity Dynamics in Complex Conditions
Source: PLoS Comput Biol. 2014 May 8;10(5):e1003602. doi: 10.1371/journal.pcbi.1003602 (PMC4014397; doi:10.1371/journal.pcbi.1003602)
Supplement: Text S1 — Supporting information methods and figures. (DOC) [file pcbi.1003602.s005.doc]

**Supplemental Instruction**

Contents

[Extended methods 3](#__RefHeading___Toc381712125)

[Figure S1 6](#__RefHeading___Toc381712126)

[Figure S2 7](#__RefHeading___Toc381712128)

[Figure S3 8](#__RefHeading___Toc381712130)

[Figure S4 9](#__RefHeading___Toc381712131)

[Figure S5 10](#__RefHeading___Toc381712132)

[Figure S6 11](#__RefHeading___Toc381712133)

[Table S1 11](#__RefHeading___Toc381712134)

[Table S2 12](#__RefHeading___Toc381712135)

[Table S3 12](#__RefHeading___Toc381712136)

[Table S4 12](#__RefHeading___Toc381712137)

[Promoter activity dynamics 13](#__RefHeading___Toc381712138)

[NaCl, Casamino acids, NaCl + Casamino acids 13](#__RefHeading___Toc381712139)

[NaCl, H_2_O_2_, NaCl + H_2_O_2_ 18](#__RefHeading___Toc381712140)

[NaCl, Ethanol, NaCl + Ethanol 22](#__RefHeading___Toc381712141)

[Casamino acids, H_2_O_2_, Casamino acids + H_2_O_2_ 26](#__RefHeading___Toc381712142)

[Casamino acids, Ethanol, Casamino acids + Ethanol 30](#__RefHeading___Toc381712143)

[H_2_O_2_, Ethanol, H_2_O_2_ + Ethanol 34](#__RefHeading___Toc381712144)

[Glucose, Casamino acids, Glucose + Casamino acids 38](#__RefHeading___Toc381712145)

[NaCl, Ethanol, Casamino acids, NaCl + Ethanol + Casamino acids 42](#__RefHeading___Toc381712146)

[NaCl, H_2_O_2_, Casamino acids, NaCl + H_2_O_2_ + Casamino acids 46](#__RefHeading___Toc381712147)

[NaCl, Ethanol, H_2_O_2_, NaCl + Ethanol + H_2_O_2_ 50](#__RefHeading___Toc381712148)

[Casamino acids, Ethanol, H_2_O_2_, Casamino acids + Ethanol + H_2_O_2_ 54](#__RefHeading___Toc381712149)

[Casamino acids, Ethanol, H_2_O_2_, NaCl, Casamino acids + Ethanol + H_2_O_2_ + NaCl 58](#__RefHeading___Toc381712150)

[Glucose, Lactose, Glucose + Lactose 62](#__RefHeading___Toc381712151)

[References 66](#__RefHeading___Toc381712152)

# Extended methods

**Generation interpolation**

Bacteria that grew in different growth conditions reached stationary phase at different times but reached similar final OD. Therefore in order to compare dynamics in different conditions, we interpolated dynamics in all conditions to a shared axis of generation times $\log_{2}\left(OD(t)/OD_{0}\right)$ with a fixed number of n=100 equally spaced points. Here, OD(t) is the OD at time t after reduction of background level (minimum OD level); OD_0_ = 1.8e-3 is the expected initial OD based on the final OD of the overnight culture OD_f_= 0.9 (overnight culture in high-brim 96-well plate with 600µl M9 + 0.2% glucose and 0.05% casamino acids in every well), divided by the dilution factor, 500 (OD_0_ = 0.9/500). We interpolated the data using different numbers of points ranging from n=60 to 250 generation data points. The results were insensitive to the number of time points n.

**Error-in-variables linear regression**

In order to find best weights for the linear combination and for the linear superposition, we use an element-wise weighted total lease squares [1]. Element-wise weighted total lease squares is an error-in-variables linear regression (A linear regression which accounts for [measurement errors](http://en.wikipedia.org/wiki/Measurement_errors) in the [independent variables](http://en.wikipedia.org/wiki/Independent_variables)).

**Computation of fit error and day-to-day experimental error**

To compute the fit error between the measured promoter activity *f* and the estimated promoter activity *g* in complex growth condition we use the above formula as followed: *f* is the median measured promoter activity (median of 4 repetitions) and *g* is the estimate promoter activity is the estimate promoter activity. *g* is calculated by multiplying the coefficients (found using linear combination, linear superposition or prediction based on pair coefficients data) times the median measured promoter activity of individual growth conditions. Relative error does not take a standard error of *f* and *g* under account.

To compute day-to-day experimental error, we measured the median experiment and compared each repetition to the median experiment. We repeated every experiment on four different days and computed for every gene and every condition the median promoter activity of four repetitions. We computed the relative error between each repetition and the median experiment to get four relative errors (between each repetition and the median experiment). The day-to-day experimental error is the median of the four relative errors. In this approach, both day-to-day experimental error and fit error are computed using the same experimental curve (the median of the repeats).

**Akaike information criterion for model selection**

For model selection we use the Aikaike information criterion on pair condition data [2]. Akaike score$A=2k-2(L)$ where L is the log likelihood and k is the number of free parameters. We compute the log likelihood of the two models, the linear combination model and the linear superposition model. The linear combination model has two free parameters, and the linear superposition model has one free parameter. Although linear superposition constraints the free parameter weight to range from 0 to 1, this is not taken into account for Akaike score. This is a limitation of the Akaike method since it does not prefer models with constraints (which lower the degree of freedom). Would it have been taken into account, the superposition model would have a lower Akaike score than with one parameter.

**Model selection between two alternative models multiplicative and additive models**

We considered two alternative one parameter models, an additive superposition model and a multiplicative superposition model:

$$P_{AB}(t)=P_{A}(t)^{(1-w_{B}^{AB})}P_{B}(t)^{w_{B}^{AB}}$$

The analysis of the multiplicative superposition model was conducted on the log space:

$$\log\left(P_{AB}(t)\right)=(1-w_{B}^{AB})\log\left(P_{A}(t)\right)+w_{B}^{AB}\log\left(P_{B}(t)\right)$$

The model becomes additive and all analysis and predictive formulas remain the same. This model gives good linear superposition fits yet we choose the additive model since it gave better predictions than a multiplicative superposition model.

**Prediction based on pair condition data formulas**

The following calculations are taken from [3]. For predicting the weights w_i_^(ijk)^ of a triplet condition, ijk based on pair condition data, we assume that the promoter activity P_ijk_ in a mixture of ijk is a linear superposition mixture of conditions:

$$P_{ijk}=w_{i}^{(ijk)}P_{i}+w_{j}^{(ijk)}P_{j}+w_{_{k}}^{(ijk)}P_{k}$$

$$w_{i}^{(ijk)}+w_{j}^{(ijk)}+w_{k}^{(ijk)}=1$$

$$P_{ijk}=(1-w_{i}^{(ijk)})P_{jk}+w_{_{i}}^{(ijk)}P_{i}=(1-w_{j}^{(ijk)})P_{ik}+w_{j}^{(ijk)}P_{j}=(1-w_{k}^{(ijk)})P_{ij}+w_{k}^{(ijk)}P_{k}$$

We then describe P_ij_,P_jk_ and P_ik_ as a linear superposition of the one component media with calculated weights:

$$P_{jk}(t)=(1-w_{k}^{(jk)})P_{j}(t)+w_{k}^{(jk)}P_{k}(t)$$

$$P_{ik}(t)=(1-w_{i}^{(ik)})P_{i}(t)+w_{i}^{(ik)}P_{k}(t)$$

When comparing weights ^wi(ij^k) we can extract six formulas:

$$w_{i}^{(ijk)}=(1-w_{k}^{(ijk)})w_{i}^{(ij)}=(1-w_{j}^{(ijk)})w_{i}^{(ik)}$$

$$w_{j}^{(ijk)}=(1-w_{k}^{(ijk)})w_{j}^{(ij)}=(1-w_{i}^{(ijk)})w_{j}^{(jk)}$$

$$w_{k}^{(ijk)}=(1-w_{i}^{(ijk)})w_{k}^{(jk)}=(1-w_{j}^{(ijk)})w_{k}^{(ik)}$$

These six equations are enough to solve w_i_^(ijk)^:

$$w_{i}^{(ijk)}=\frac{w_{i}^{(ik)}-w_{i}^{(ik)}w_{j}^{(jk)}}{1-w_{i}^{(ik)}w_{j}^{(jk)}}=\frac{w_{i}^{(ij)}-w_{i}^{(ij)}w_{k}^{(jk)}}{1-w_{i}^{(ij)}w_{k}^{(jk)}}$$

$$w_{j}^{(ijk)}=\frac{w_{j}^{(jk)}-w_{j}^{(jk)}w_{i}^{(ik)}}{1-w_{j}^{(jk)}w_{i}^{(ik)}}=\frac{w_{j}^{(ij)}-w_{j}^{(ij)}w_{k}^{(ik)}}{1-w_{j}^{(ij)}w_{k}^{(ik)}}$$

$$w_{k}^{(ijk)}=\frac{w_{k}^{(ik)}-w_{k}^{(ik)}w_{j}^{(ij)}}{1-w_{k}^{(ik)}w_{j}^{(ij)}}=\frac{w_{k}^{(jk)}-w_{k}^{(jk)}w_{i}^{(ij)}}{1-w_{k}^{(jk)}w_{i}^{(ij)}}$$

More generally, for predicting the weights w_i_^(1…N)^ of N conditions:

$w_{i}^{(1...N)}=\frac{w_{i}^{\neq j}-w_{i}^{\neq j}w_{j}^{\neq i}}{1-w_{i}^{\neq j}w_{j}^{\neq i}}$

Where ≠j means all media but j.

# Figure S1

#
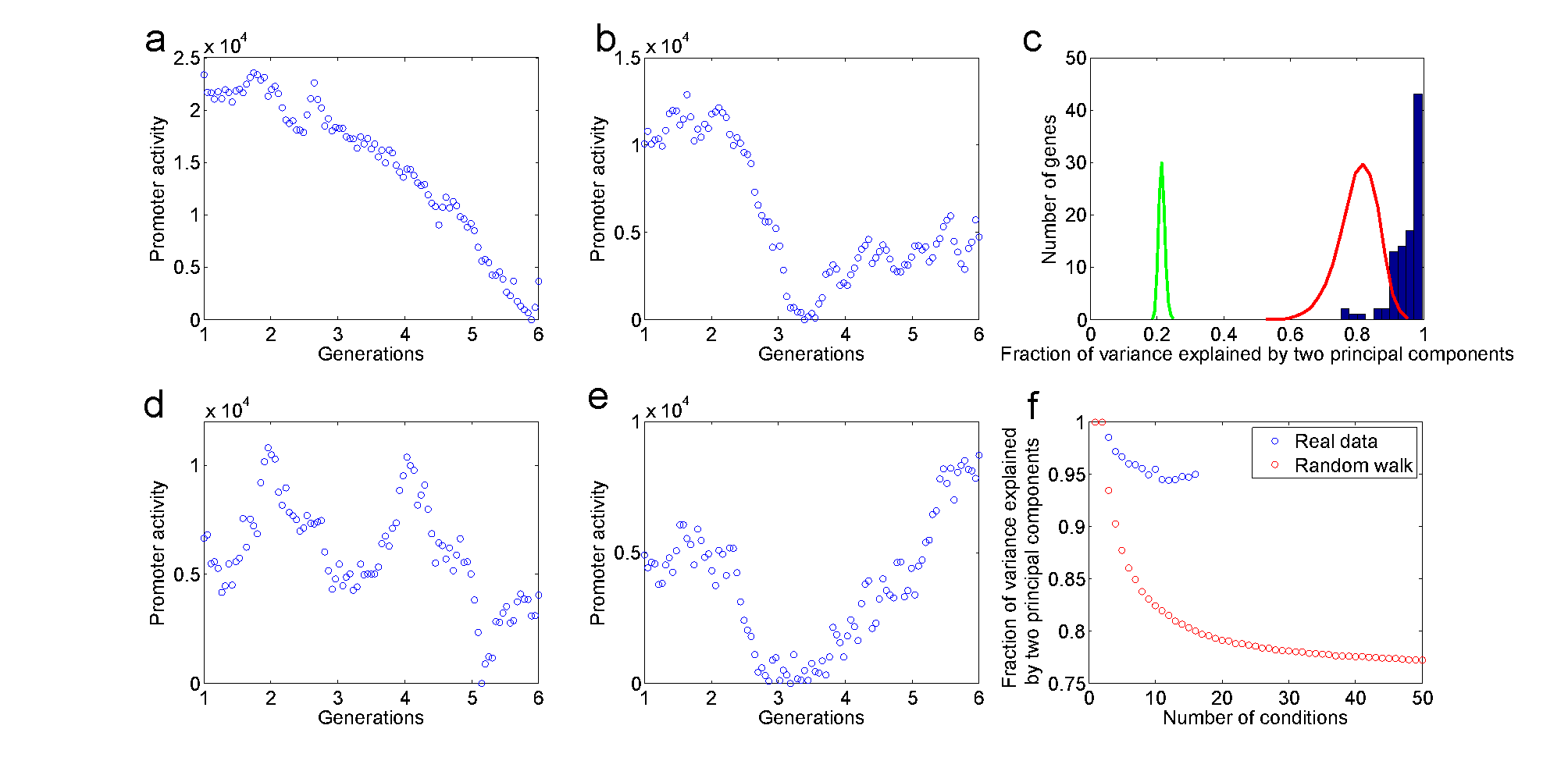


Two PCs explain much more variance than expected in randomized data

(a,b,d,e) 4 random curves mimicking random walk promoter activity dynamics (c) Blue – fraction of variance explained by the first two principal components for all 94 promoters in 16 environments (The same histogram as in figure 6d). Red – fraction of variance explained by the first two principal components for 94 genes each with 16 random walk dynamics. Green -- fraction of variance explained by the first two principal components for 94 genes each with 16 random dynamics. (f) The explained variance by two principal components decreases as a function of the number of conditions measured for each gene. Red – random walk data. Error bars are smaller than the dots themselves. Blue – experimental data. For the measured conditions, the median fraction of explained variance ranges between 95%-100% as a function of the number of environments for each gene (a median 95% explained variance is observed for all 16 environments in all genes).

# Figure S2

#
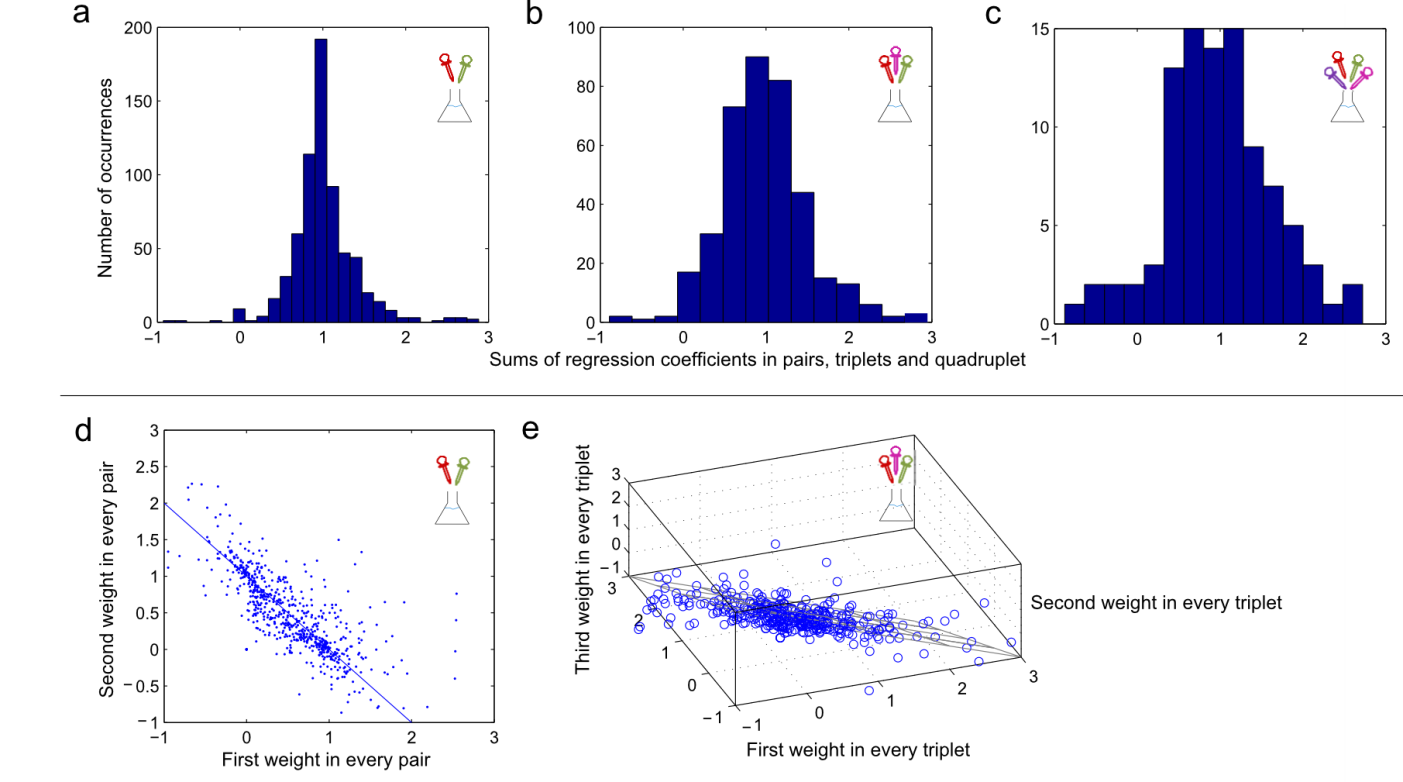


The sum of weights (regression coefficients of linear combination) is distributed around one.

(a) Distribution of sum of weights for all promoters in pair conditions (b) triplets (c) quadruplet (d) Weight 1 versus weight 2 in all pair conditions. Line is w1+w2=1 (e) Weights 1, 2 and 3 for all triplet experiments. Grey plane is w1+w2+w3=1

# Figure S3


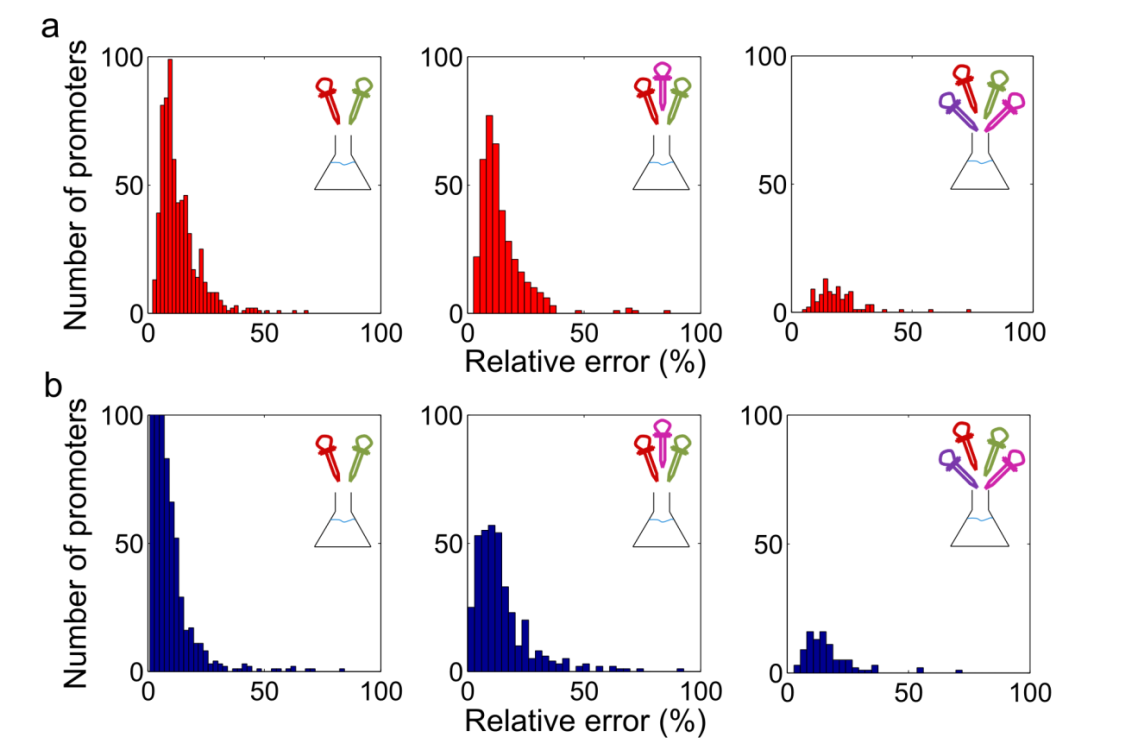


Error in linear superposition fit is similar to or smaller than the day-day experimental error.

(a) Day-to-day relative error histogram – Relative error between dynamics curves from experiments on different days in all 6 pairs of conditions (top left histogram), all 4 triplets (top middle histogram), the quadruplet (top right histogram) (b) Fit relative error histogram – Relative error of best fit linear superposition to dynamics in all 6 pairs of conditions (bottom left histogram), all 4 triplets (bottom middle histogram), the quadruplet (bottom right histogram)

# Figure S4

**
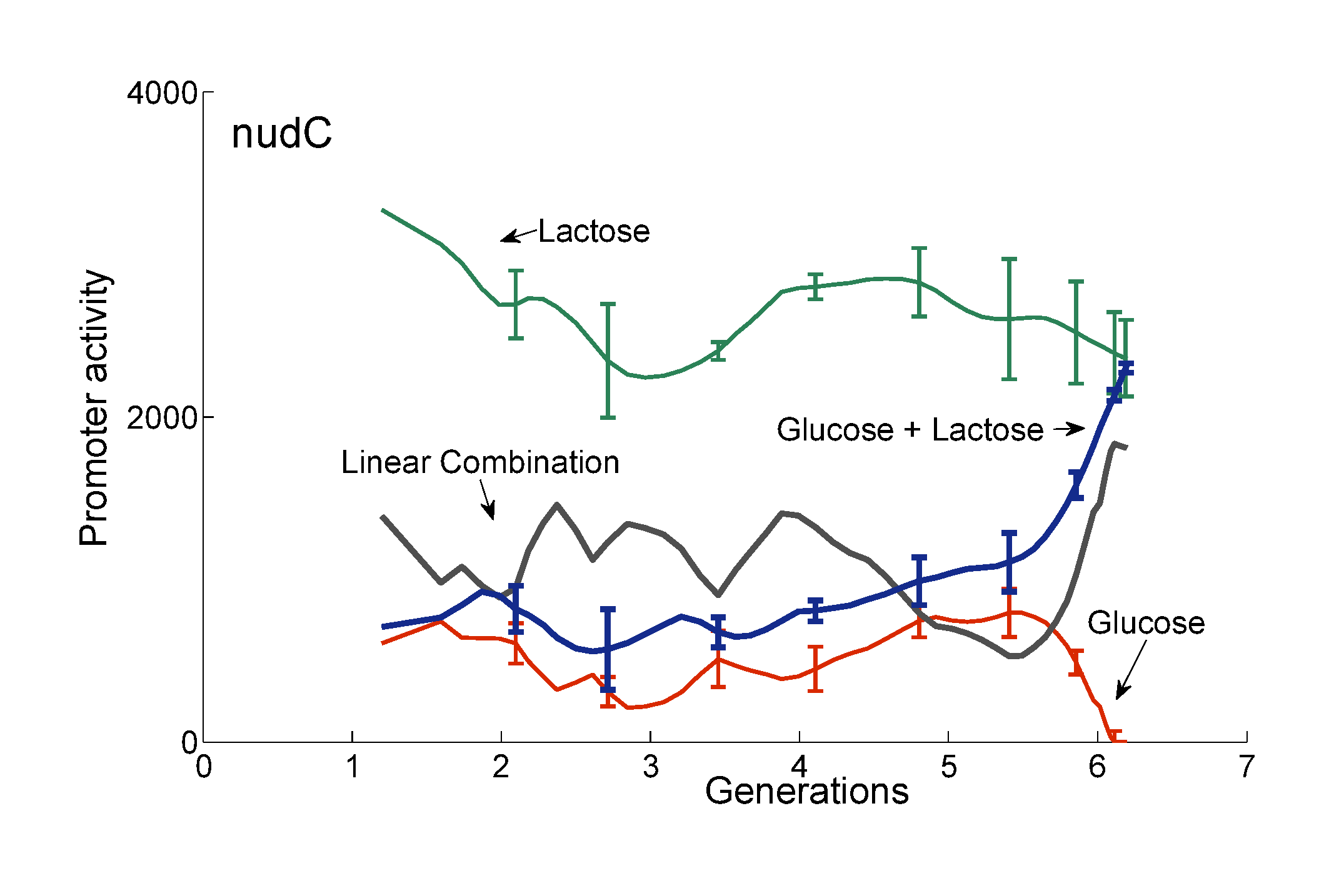
**

An example of deviation from linear combination is found in the nudC promoter in a diauxic shift experiment. Promoter activity dynamics in a mixture of 0.04% glucose and 0.4% lactose (Blue line) is far from the best fit linear combination of dynamics of glucose or lactose alone (Black line). Error bars are standard error between three independent experiments on different days.

# Figure S5


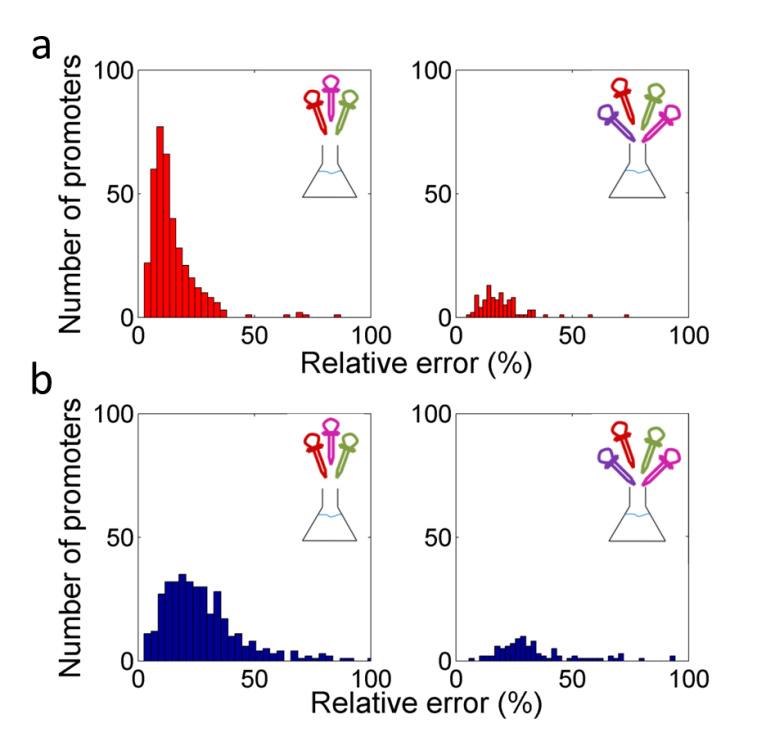


Errors in prediction based on pair weights are about two times larger than the day-to-day experimental error.

(a) Day-to-day relative error histogram – Relative error between dynamics curves from experiments on different days in all 4 triplets (top left histogram) and the quadruplet (top right histogram) (b) Prediction relative error histogram – Relative error of prediction based on pair weights to dynamics in all 4 triplets (bottom left histogram) and the quadruplet (bottom right histogram)

# Figure S6

**
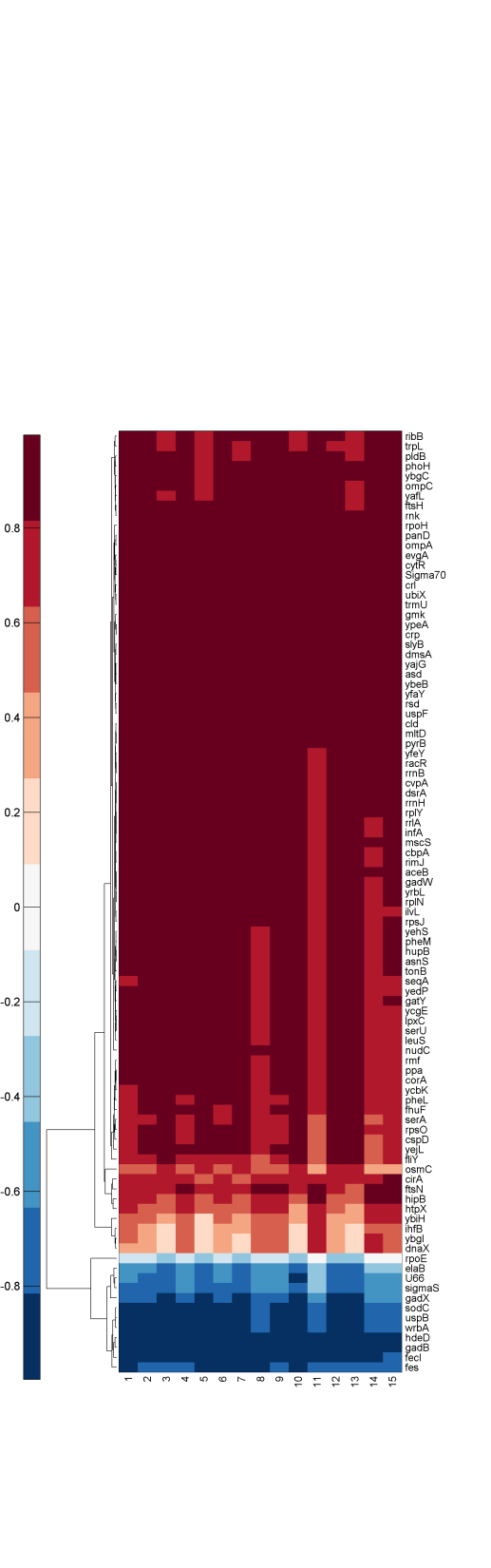

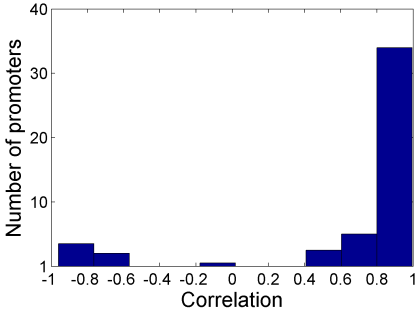

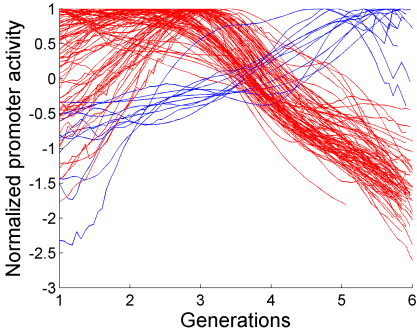

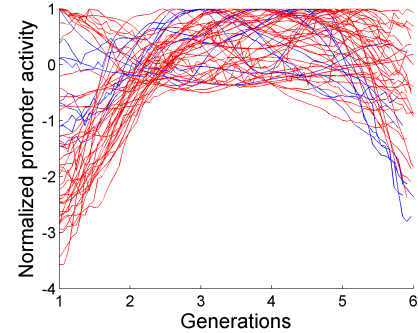
**

a

b

c

d

PC1

PC2

The first PC of most promoters is highly correlated to growth rate.

(a) A heat map of the correlation between the first principal component and the growth rate in every condition is plotted. Promoters are in the rows and conditions are in the columns.

The growth conditions 1-15 are:

1) M9 + glucose 0.2% + casamino acids 0.05%

2) M9 + glucose 0.2% + casamino acids 0.05% + ethanol 3%

3) M9 + glucose 0.2% + casamino acids 0.05% + H2O2 10uM

4) M9 + glucose 0.2% + ethanol 3%

5) M9 + glucose 0.2% + H2O2 10uM

6) M9 + glucose 0.2% + H2O2 10uM + ethanol 3%

7) M9 + glucose 0.2% + NaCl 300 mM

8) M9 + glucose 0.2% + NaCl 300 mM + casamino acids 0.05%

9) M9 + glucose 0.2% + NaCl 300 mM + ethanol 3%

10) M9 + glucose 0.2% + NaCl 300 mM + H2O2 10uM

11) M9 + glucose 0.2% + casamino acids 0.05% + H2O2 10uM + ethanol 3%

12) M9 + glucose 0.2% + NaCl 300 mM + casamino acids 0.05% + ethanol 3%

13) M9 + glucose 0.2% + NaCl 300 mM + casamino acids 0.05% + H2O2 10uM

14) M9 + glucose 0.2% + NaCl 300 mM + casamino acids 0.05% + H2O2 10uM + ethanol 3%

15) M9 + glucose 0.2% + NaCl 300 mM + H2O2 10uM + ethanol 3%

(b) A histogram of the correlation between the first principal component and the growth rate. (c) The first principal component of promoters – in red all positive correlated with growth rate first PCs, in blue – negative correlated first PCs.

(d) The second principal component of promoters – in red all positive correlated with growth rate first PCs, in blue – negative correlated second PCs.

# Table S1

A gene table with biological description is provided in an external excel file.

# Table S2

A table with the dynamics growth rate for all A,B,C,D condition combinations is provided in an external excel file.

# Table S3

A table with the weights of the linear superposition and errors for all promoters and A,B,C,D condition combinations is provided in an external excel file.

# Table S4

A table with the weights of the linear combination and errors for all promoters in the diauxic shift is provided in an external excel file.

# Promoter activity dynamics

In each figure there are 12 promoter gene activities in different conditions, thus for every combination there are 8 graphs (for the 96 well promoter plate).

Title signifies the promoter gene name

X axis is Generations

Y axis is the promoter acticity

Standard medium – M9 + Glucose 0.2%

## NaCl, Casamino acids, NaCl + Casamino acids

Red – Standard medium + NaCl 300mM

Green – Standard medium + Casamino acids 0.05%

Blue – Standard medium + NaCl 300mM + Casamino acids 0.05%

Black – best fit linear superposition


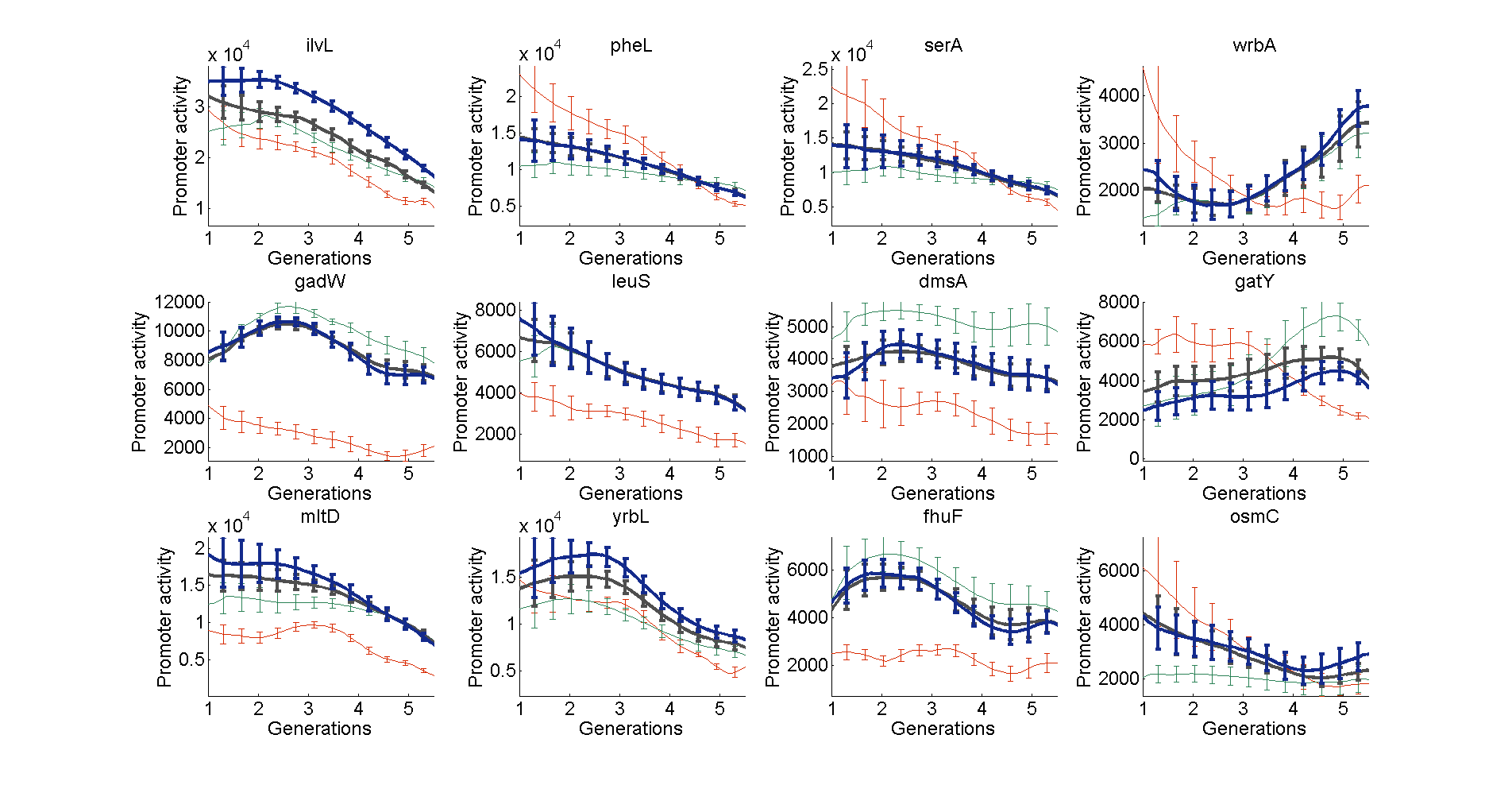


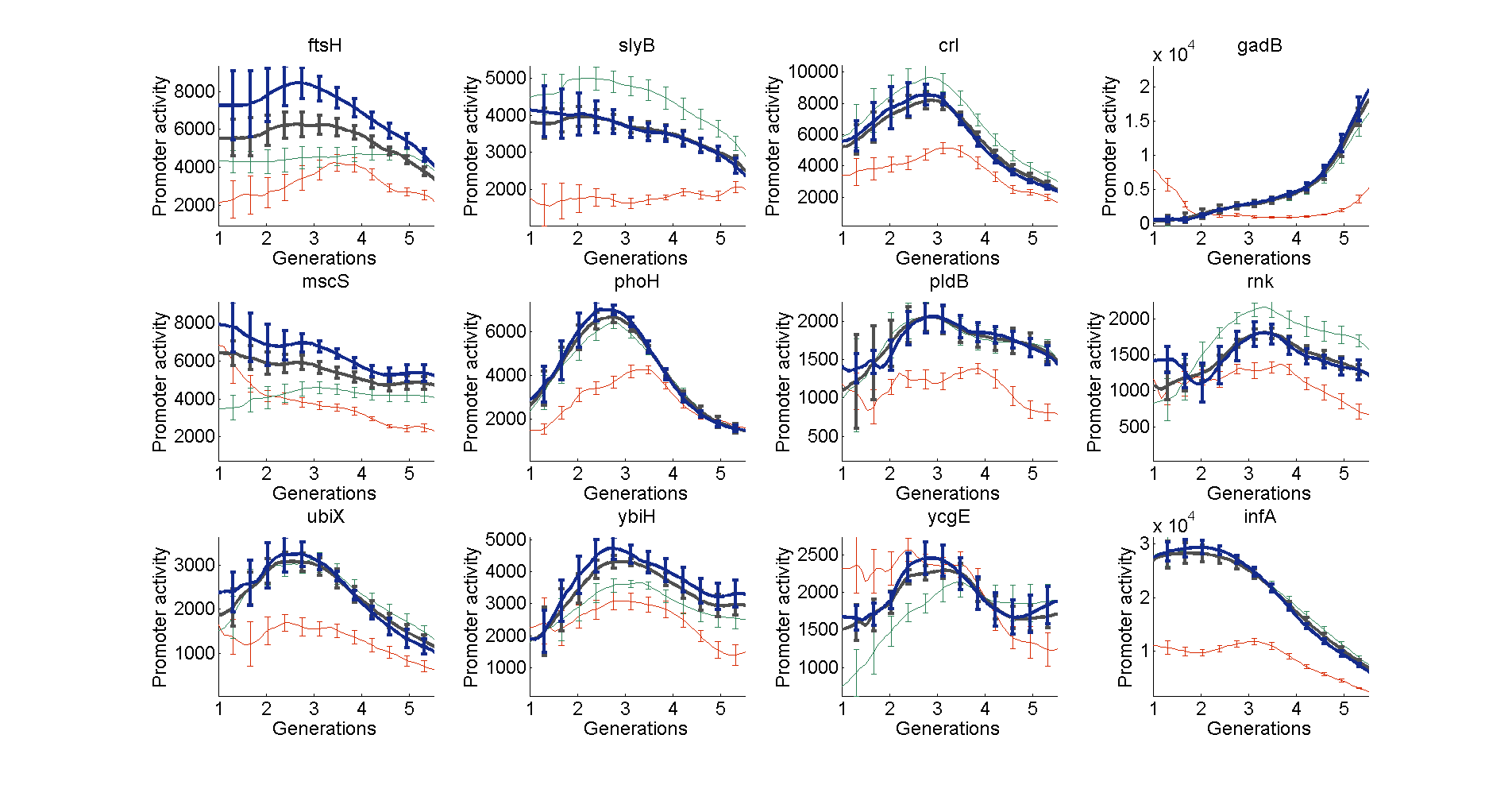


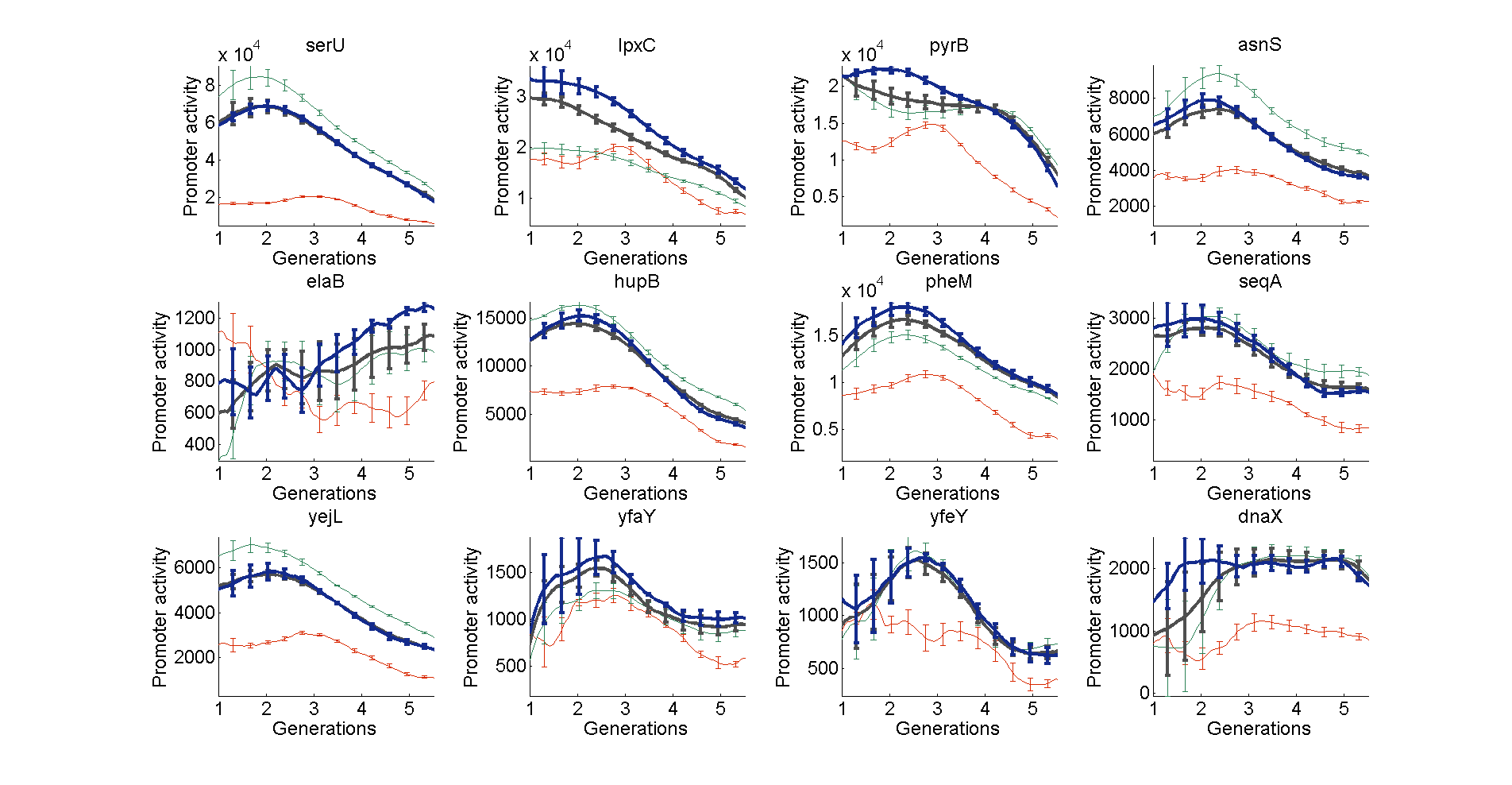


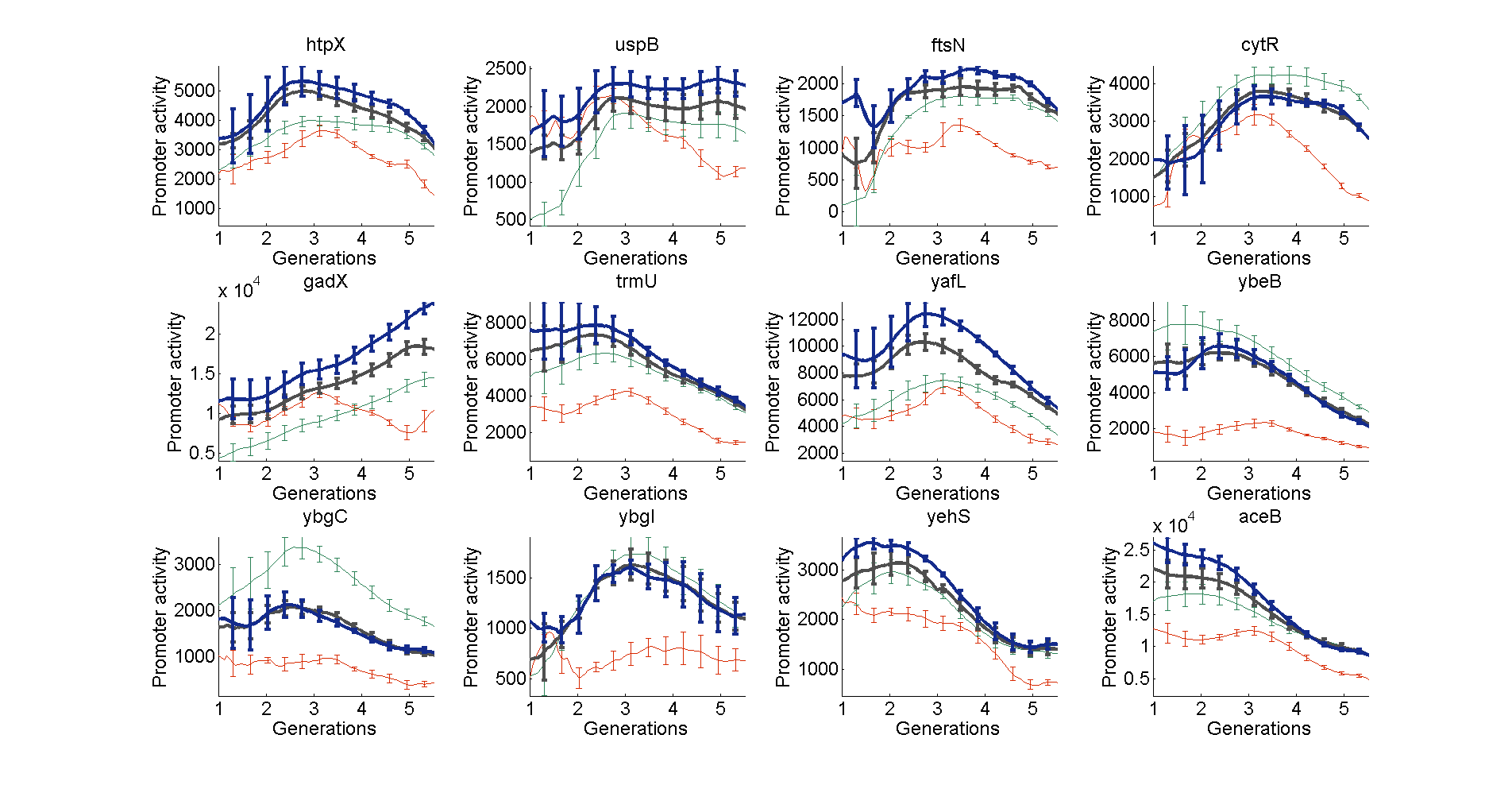


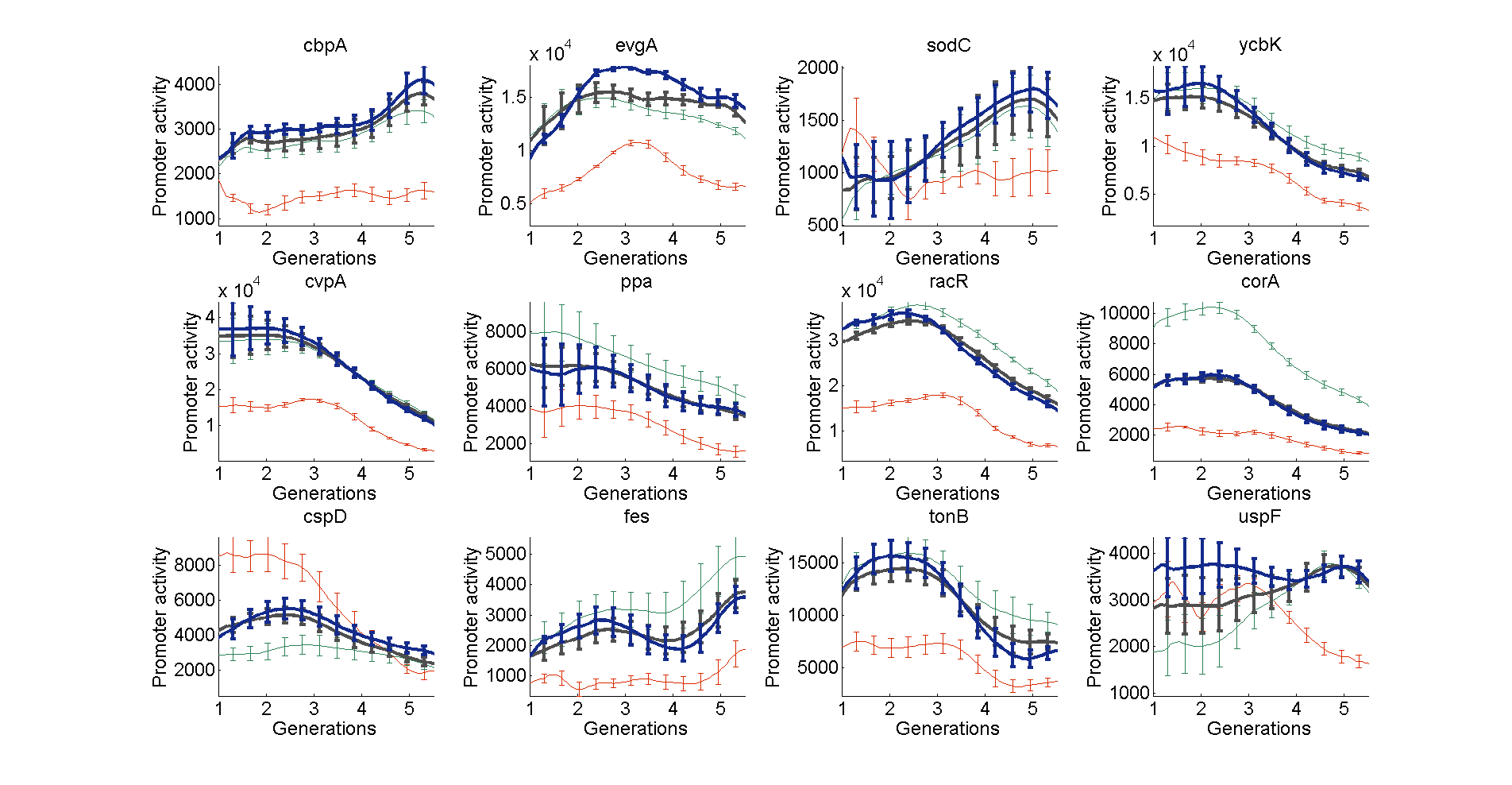


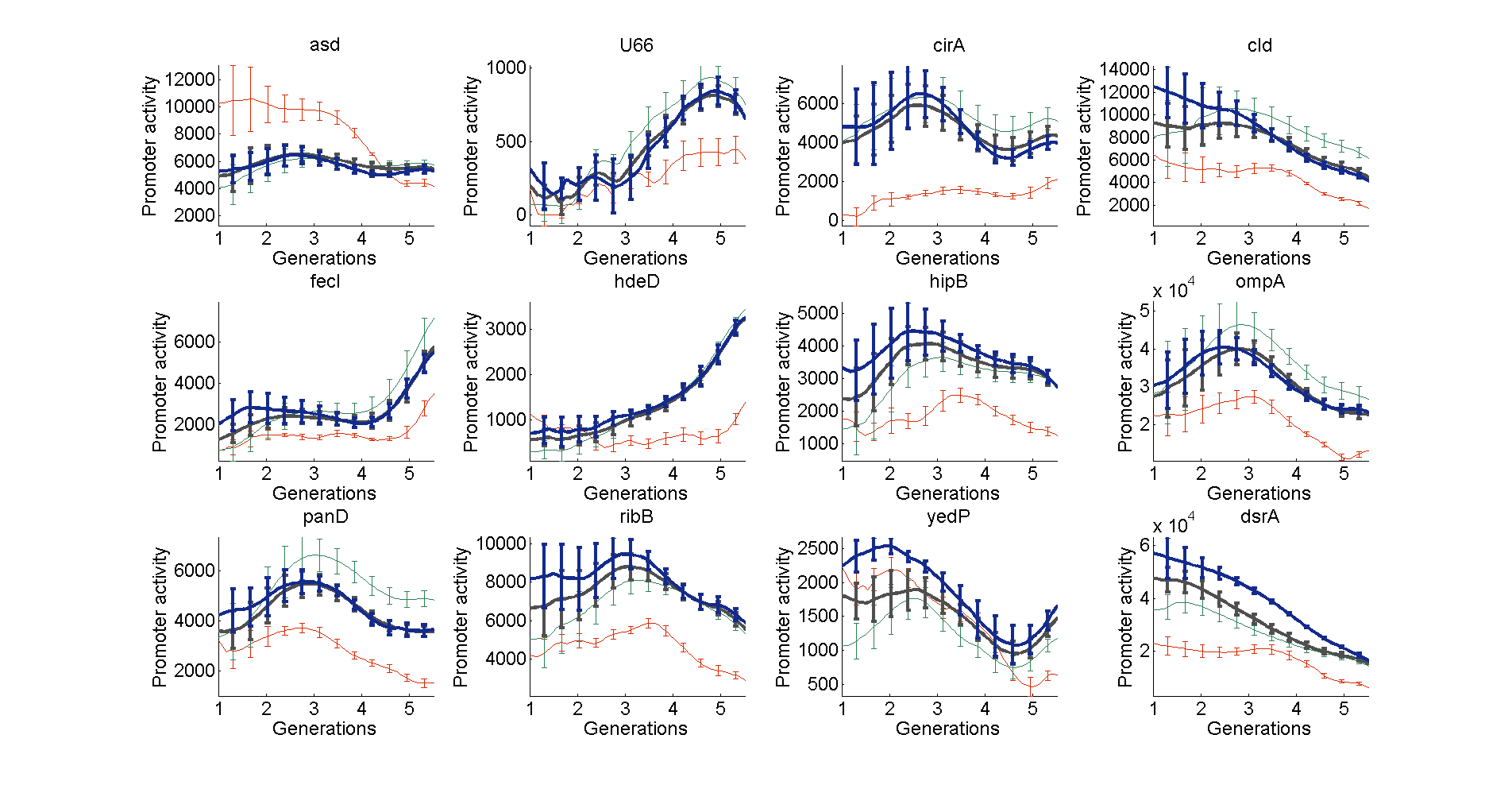


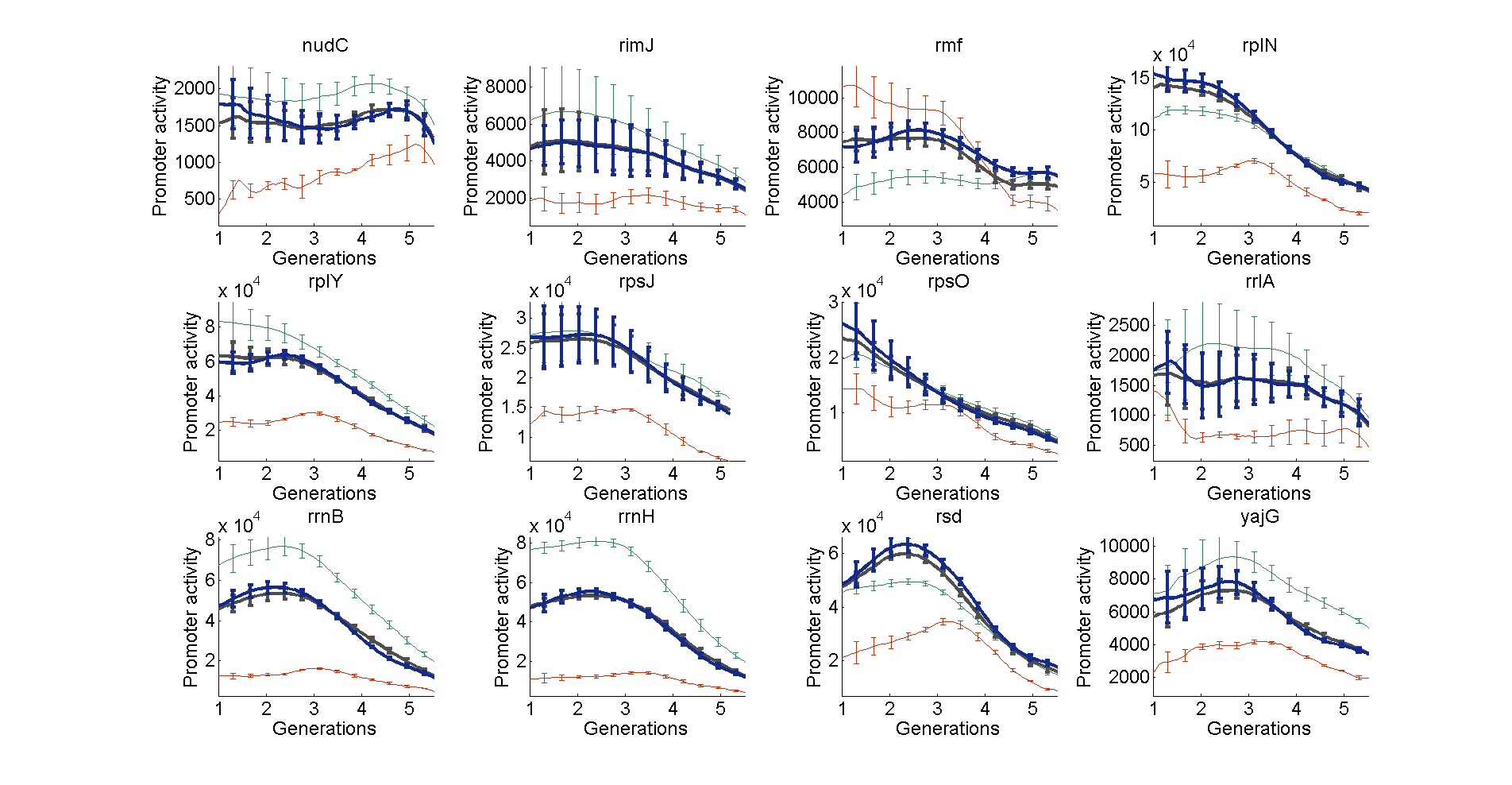


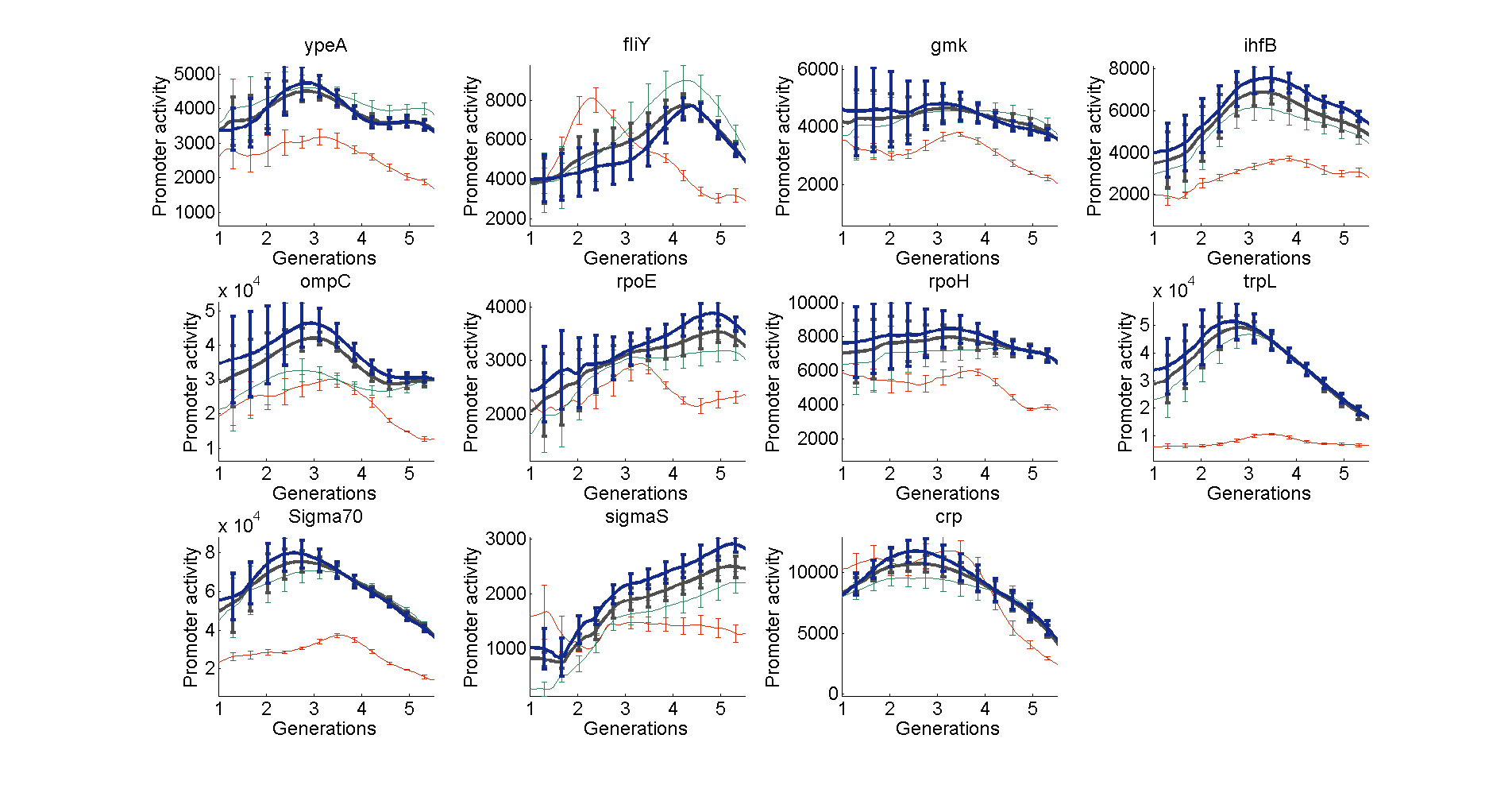


## NaCl, H_2_O_2_, NaCl + H_2_O_2_

Red – Standard medium + NaCl 300mM

Green – Standard medium + H_2_O_2_ 10µM

Blue – Standard medium + NaCl 300mM + H_2_O_2_ 10µM

Black – best fit linear superposition


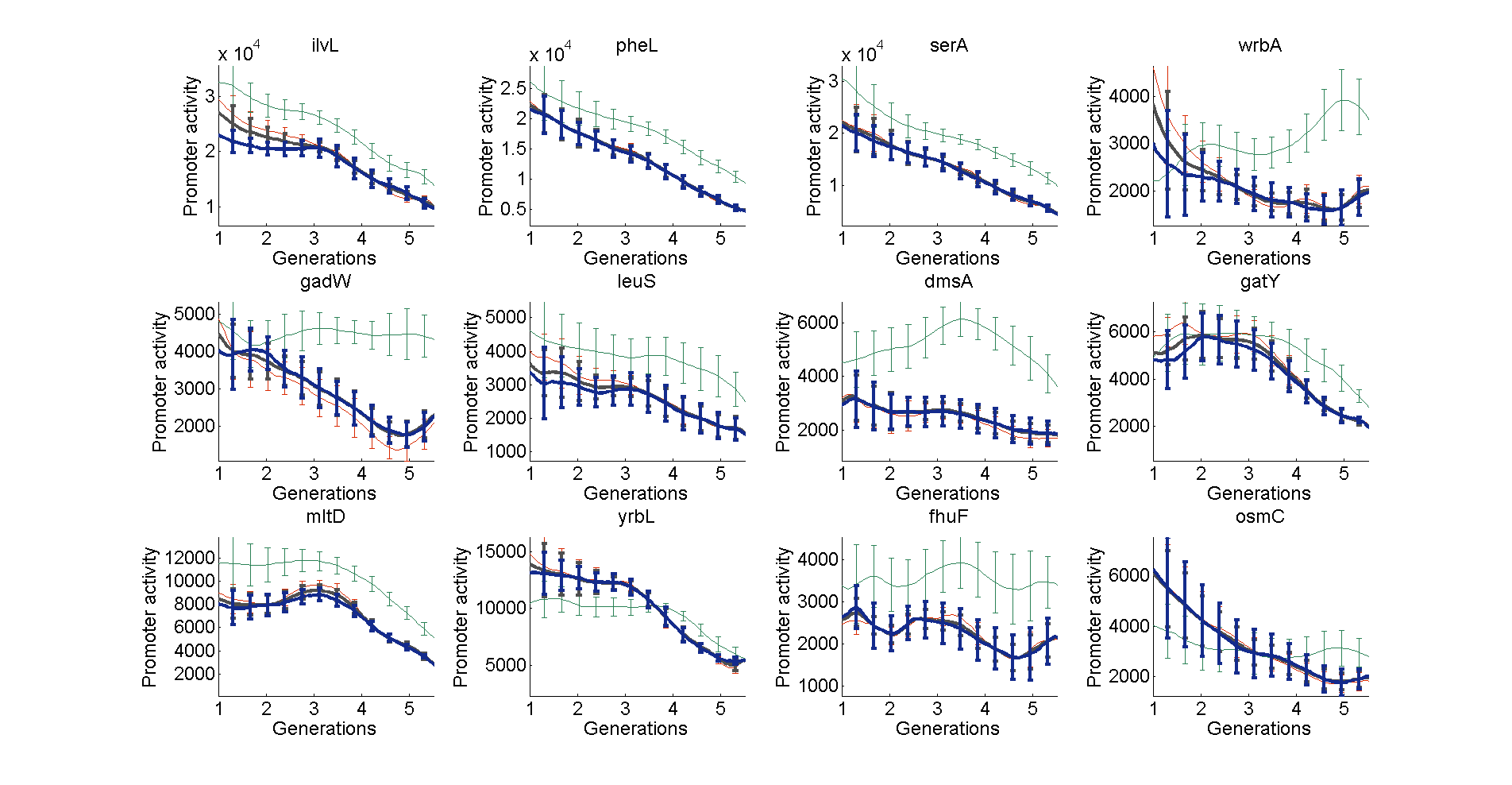


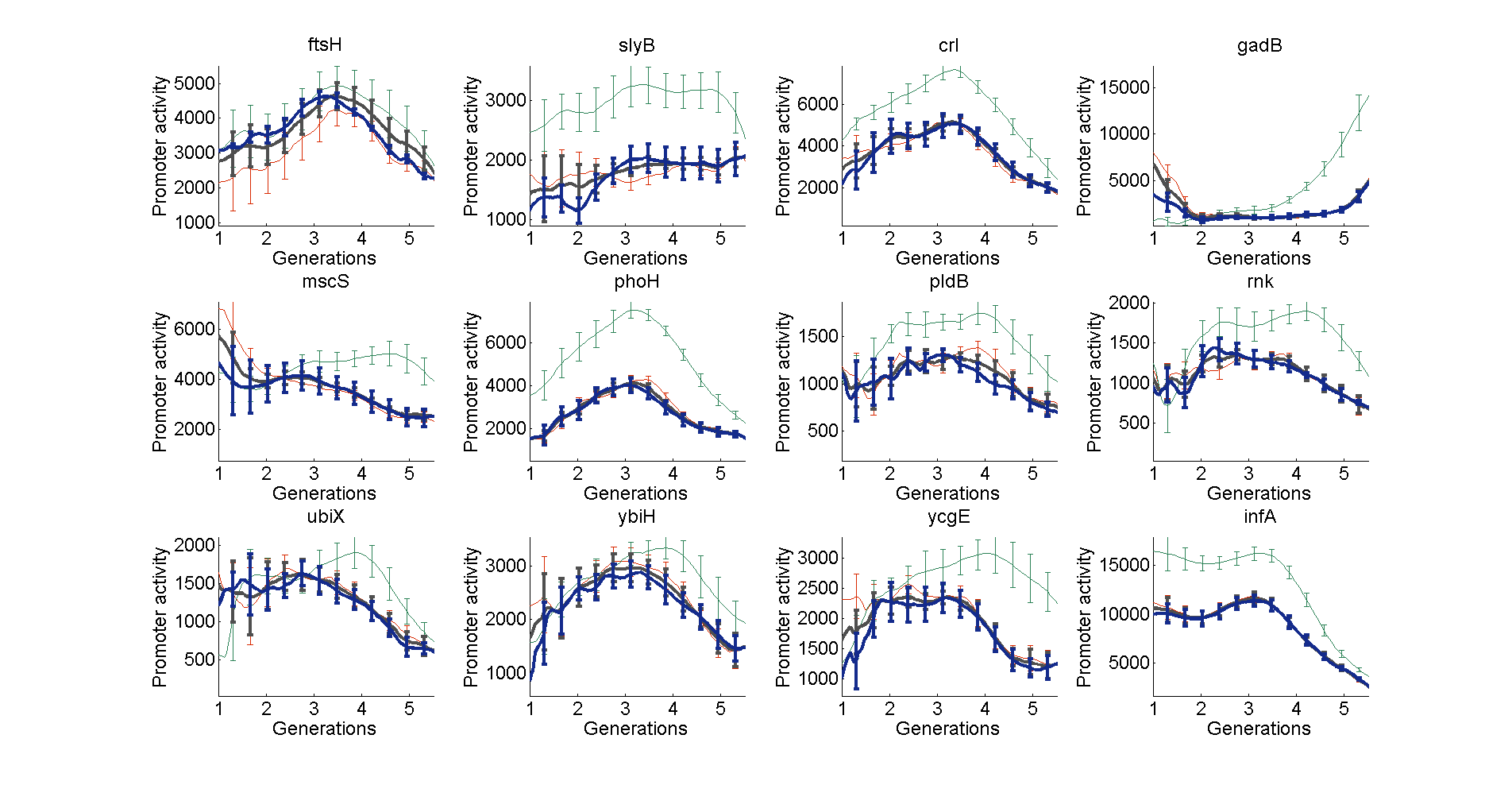


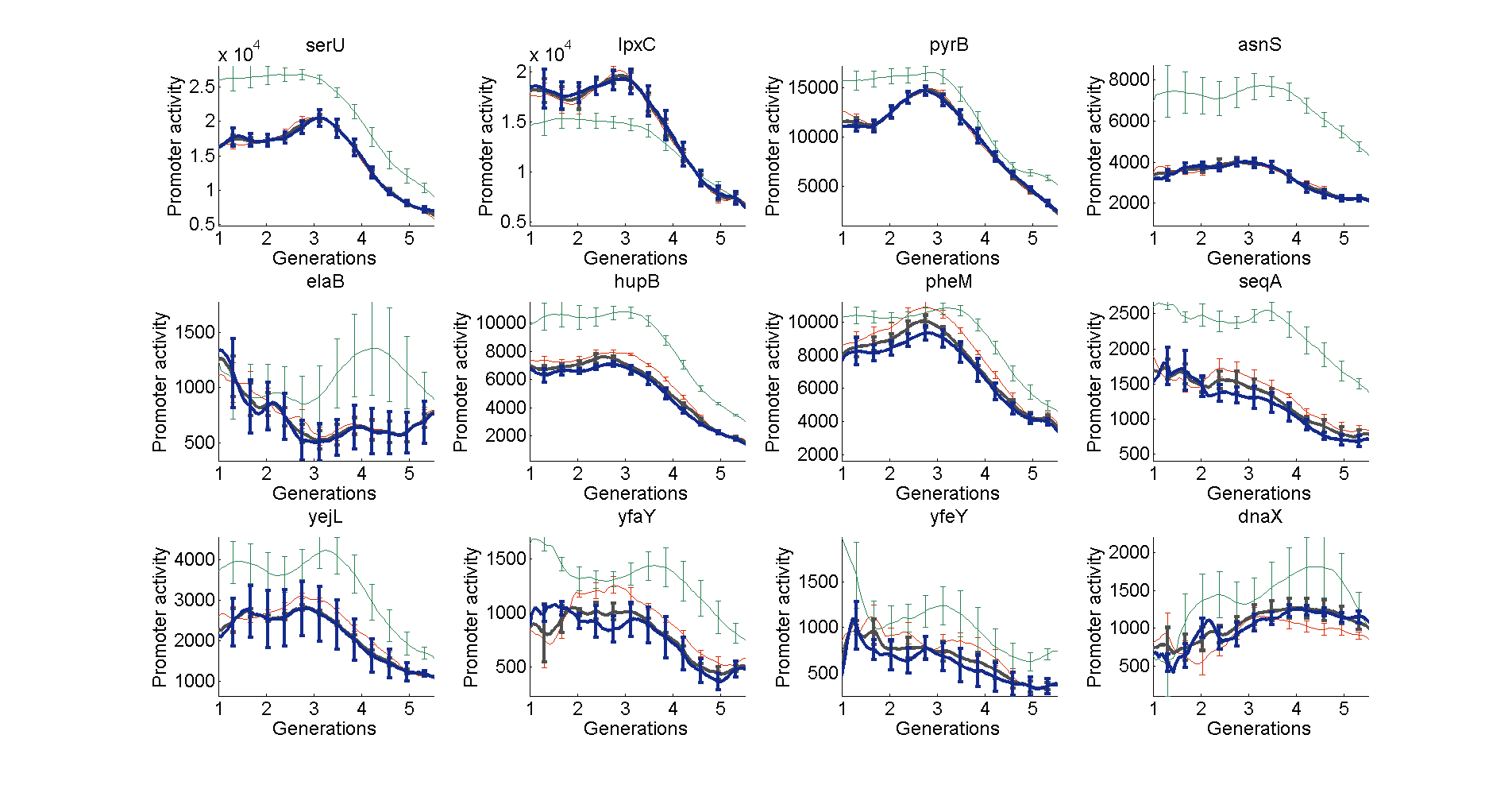


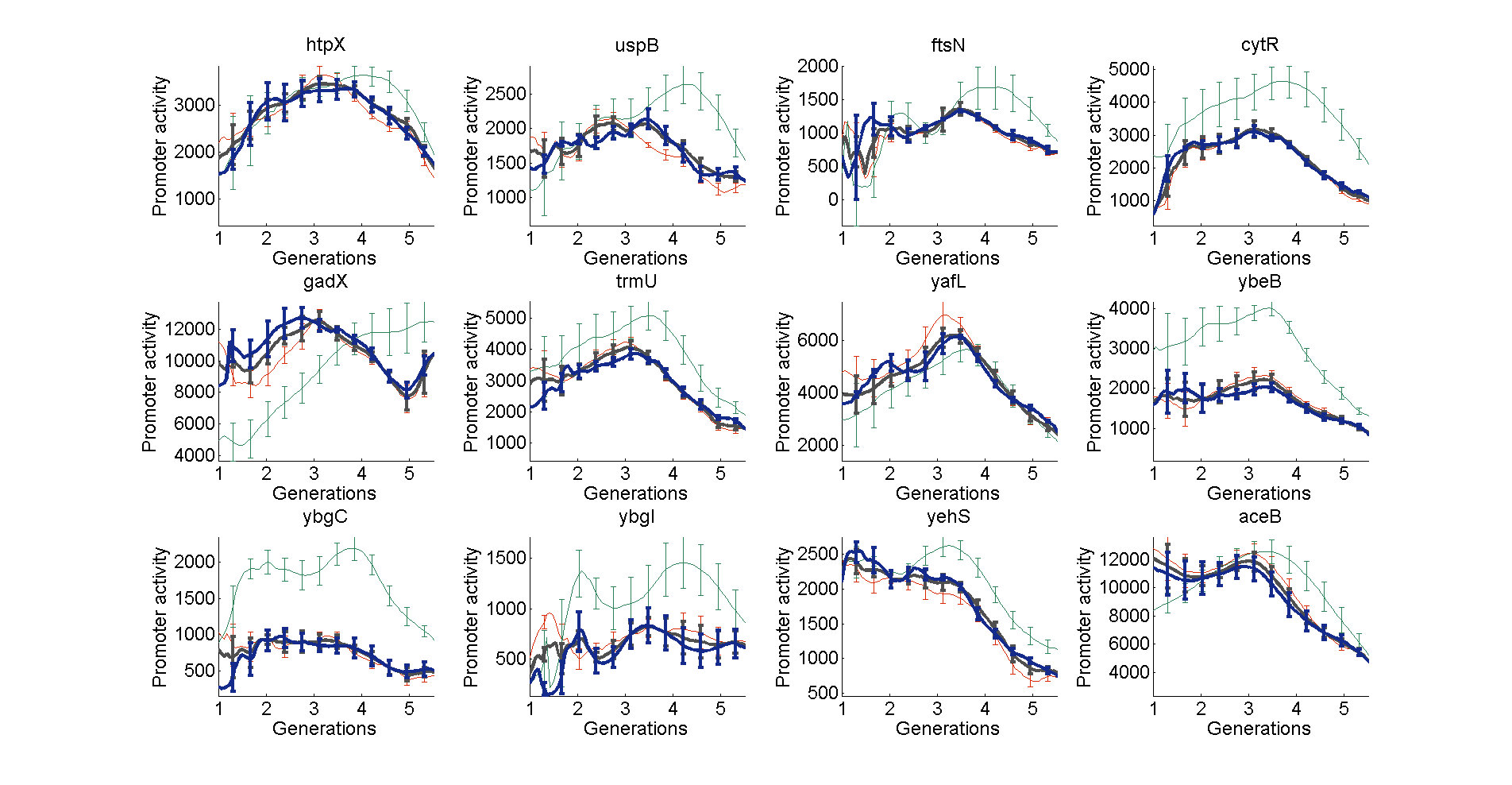


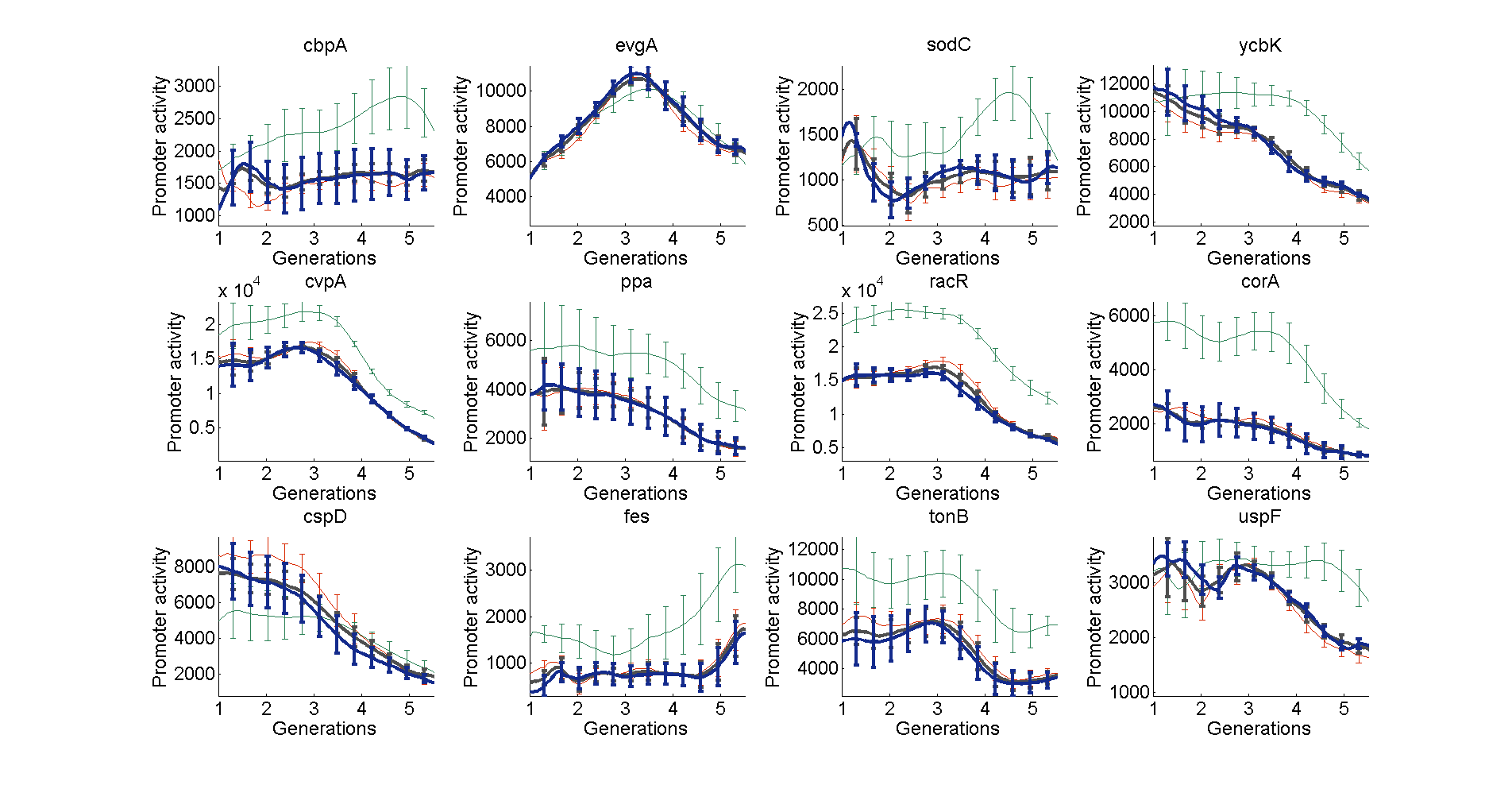


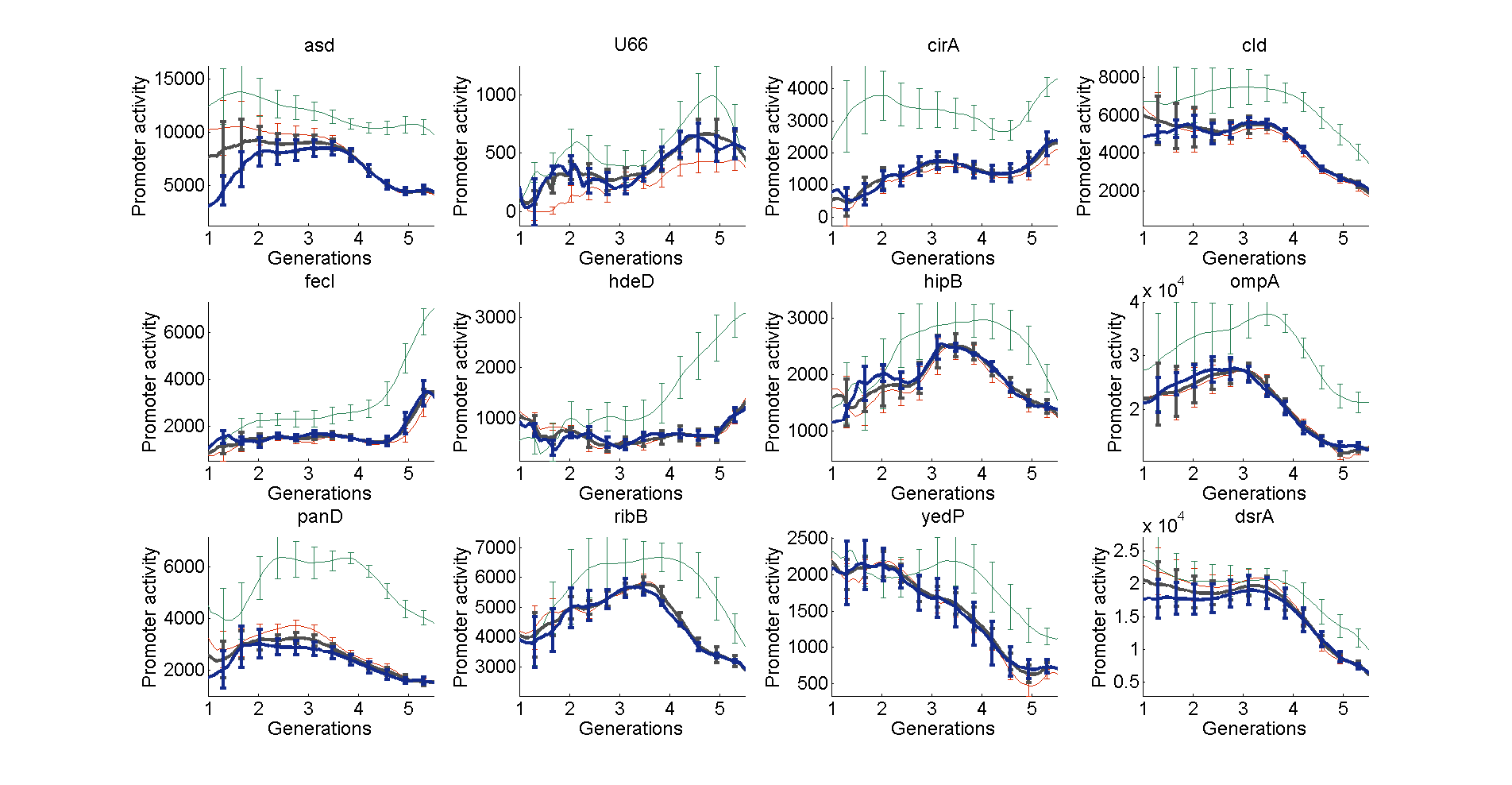


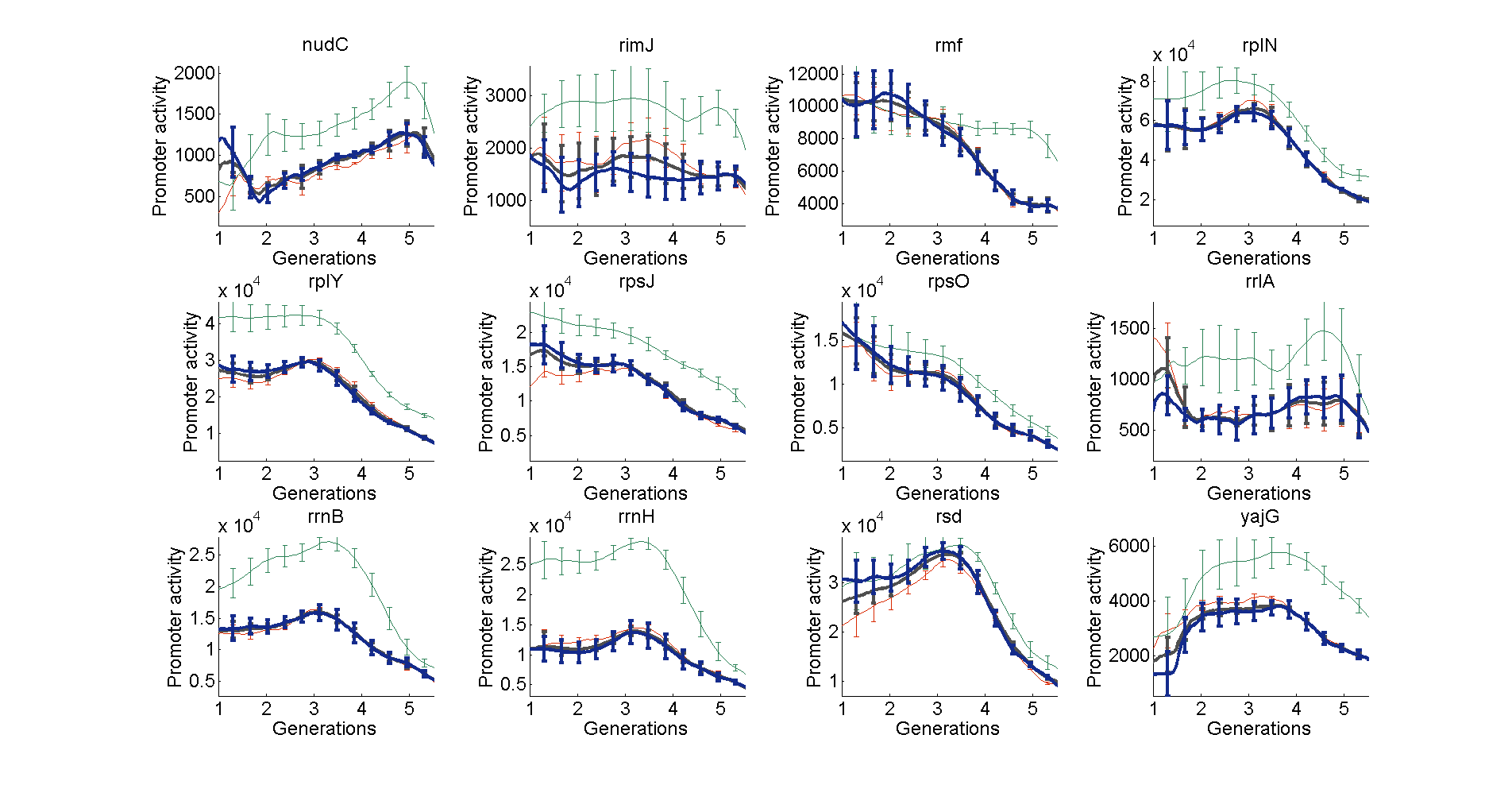


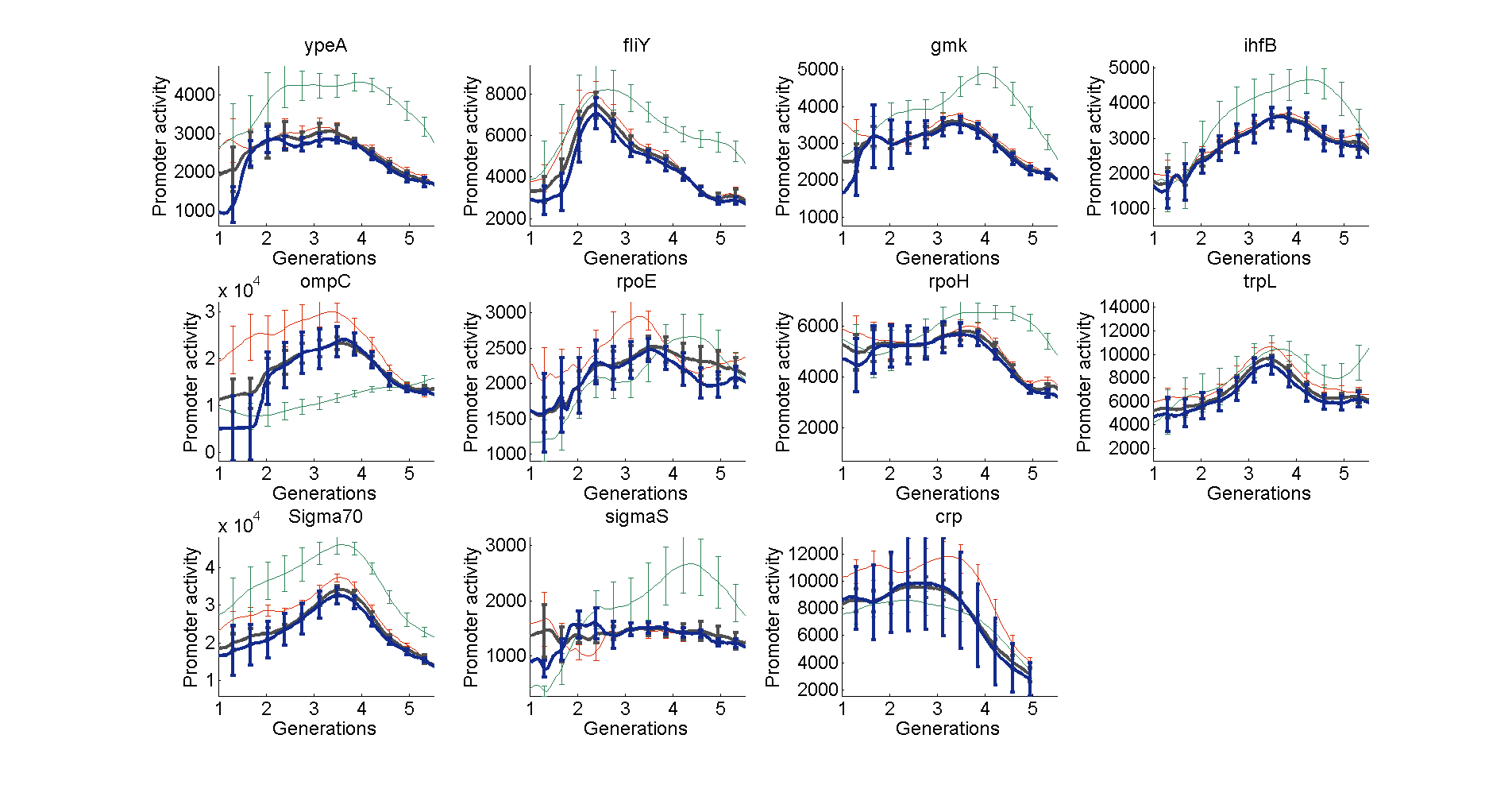


## NaCl, Ethanol, NaCl + Ethanol

Red – Standard medium + NaCl 300mM

Green – Standard medium + Ethanol 3%

Blue – Standard medium + NaCl 300mM + Ethanol 3%

Black – best fit linear superposition


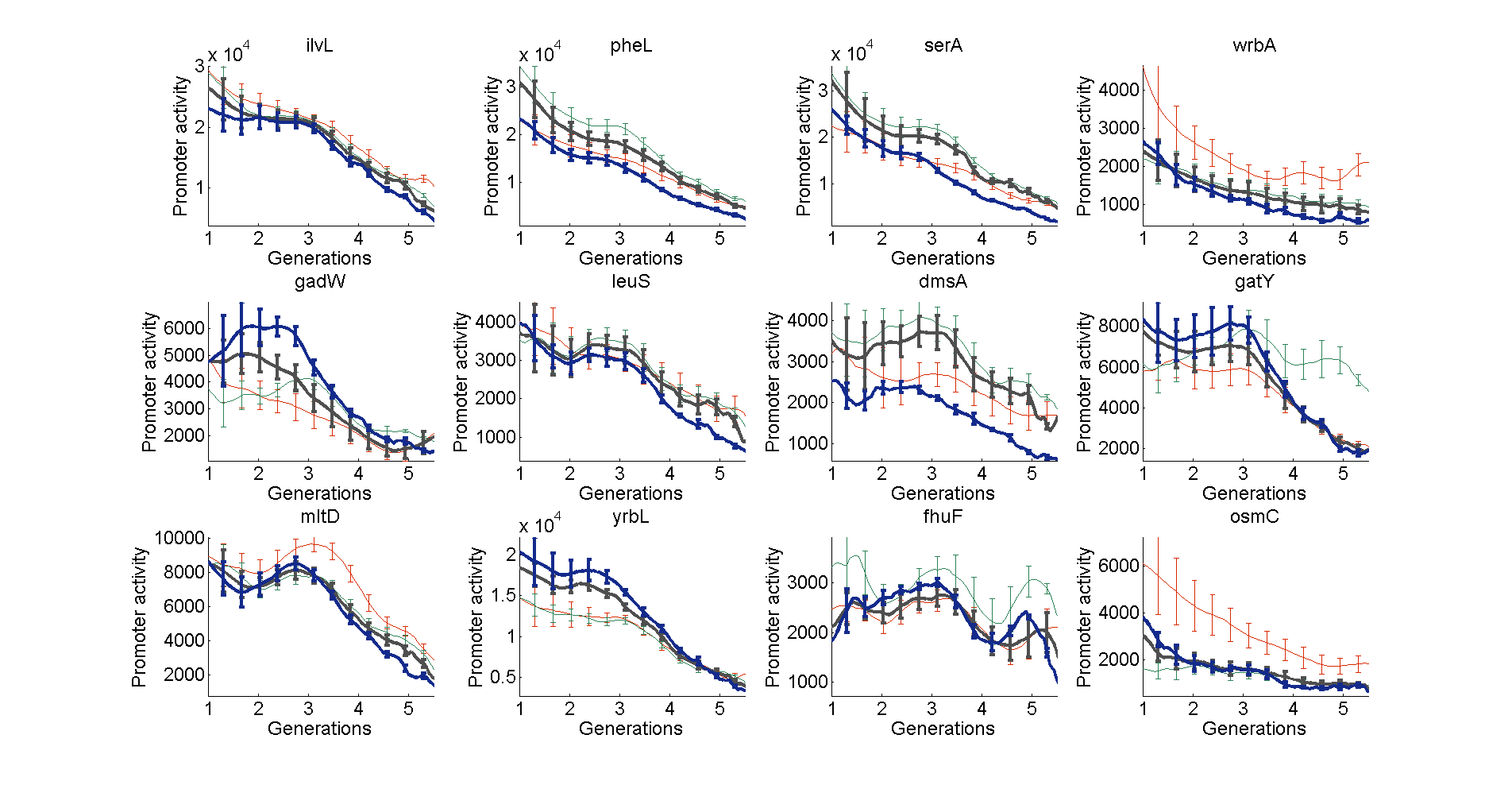


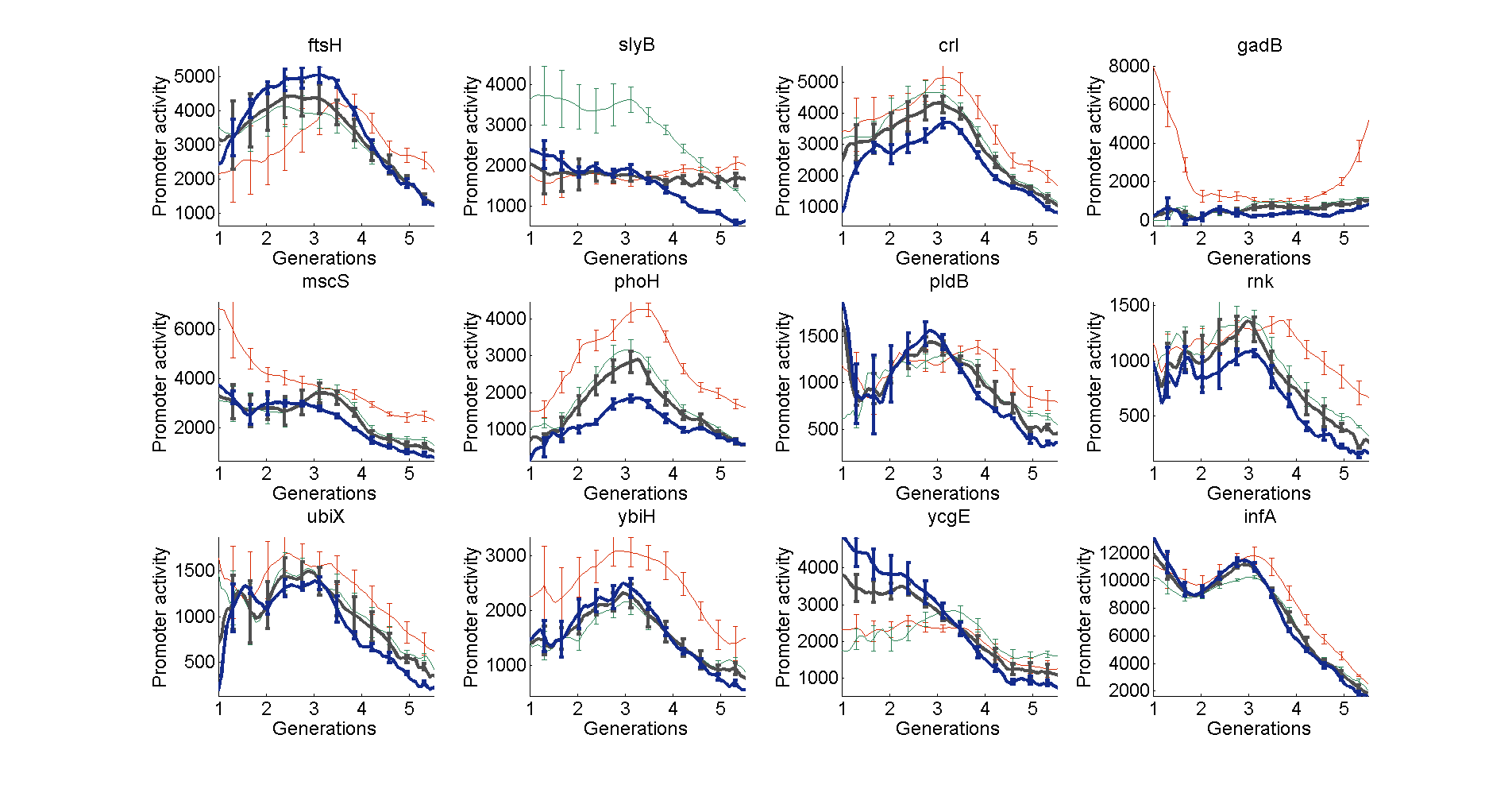


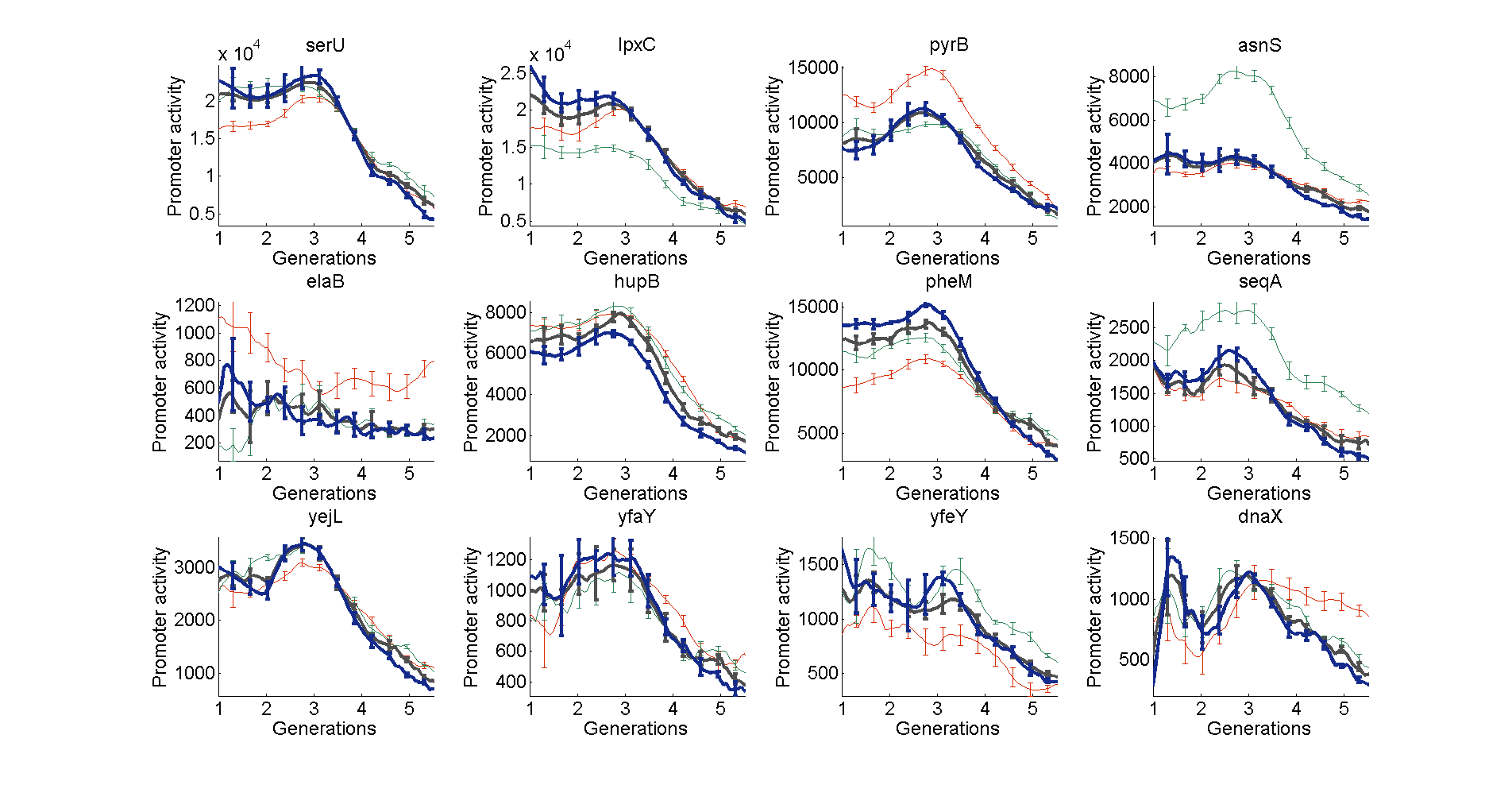


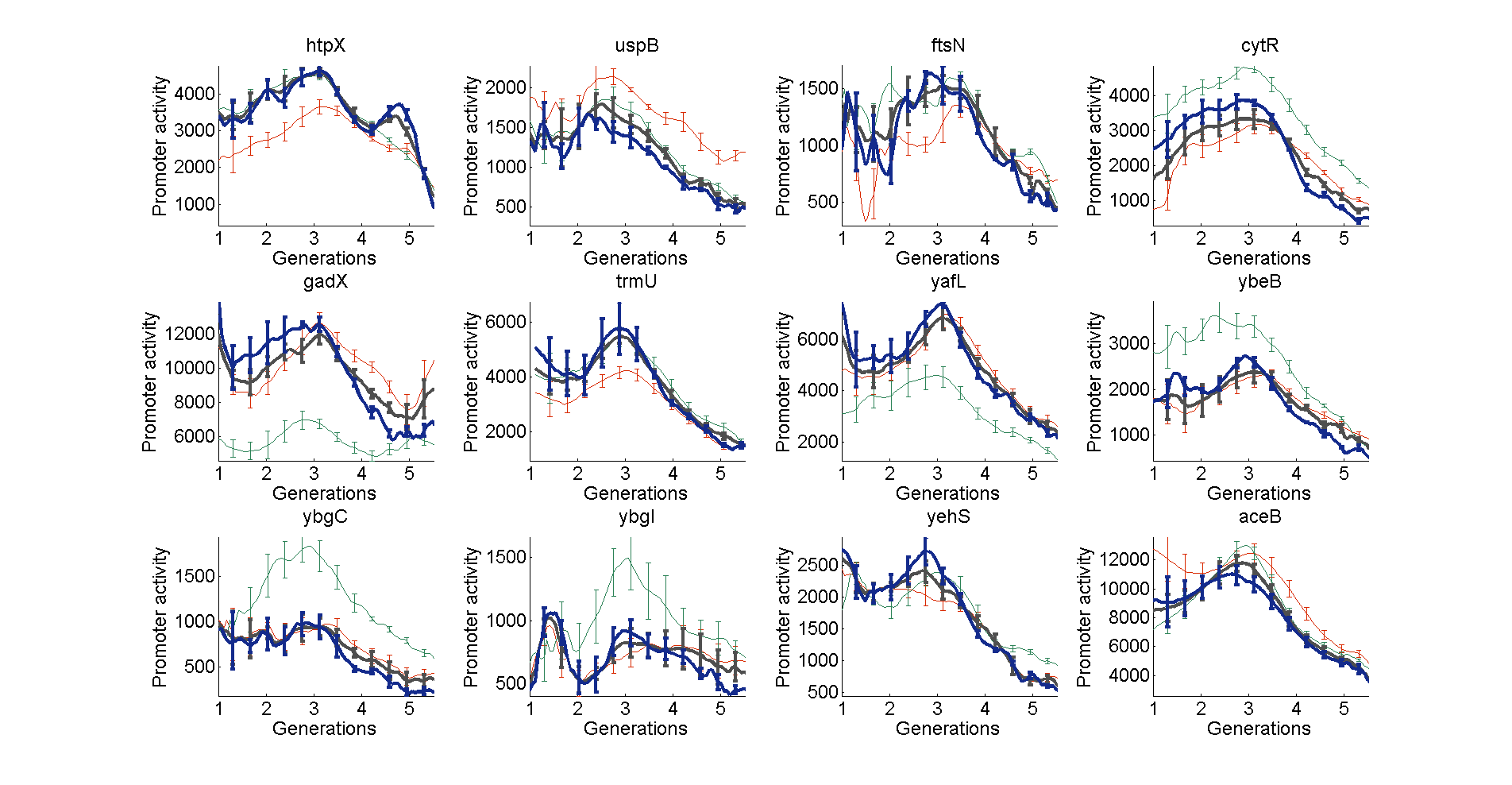


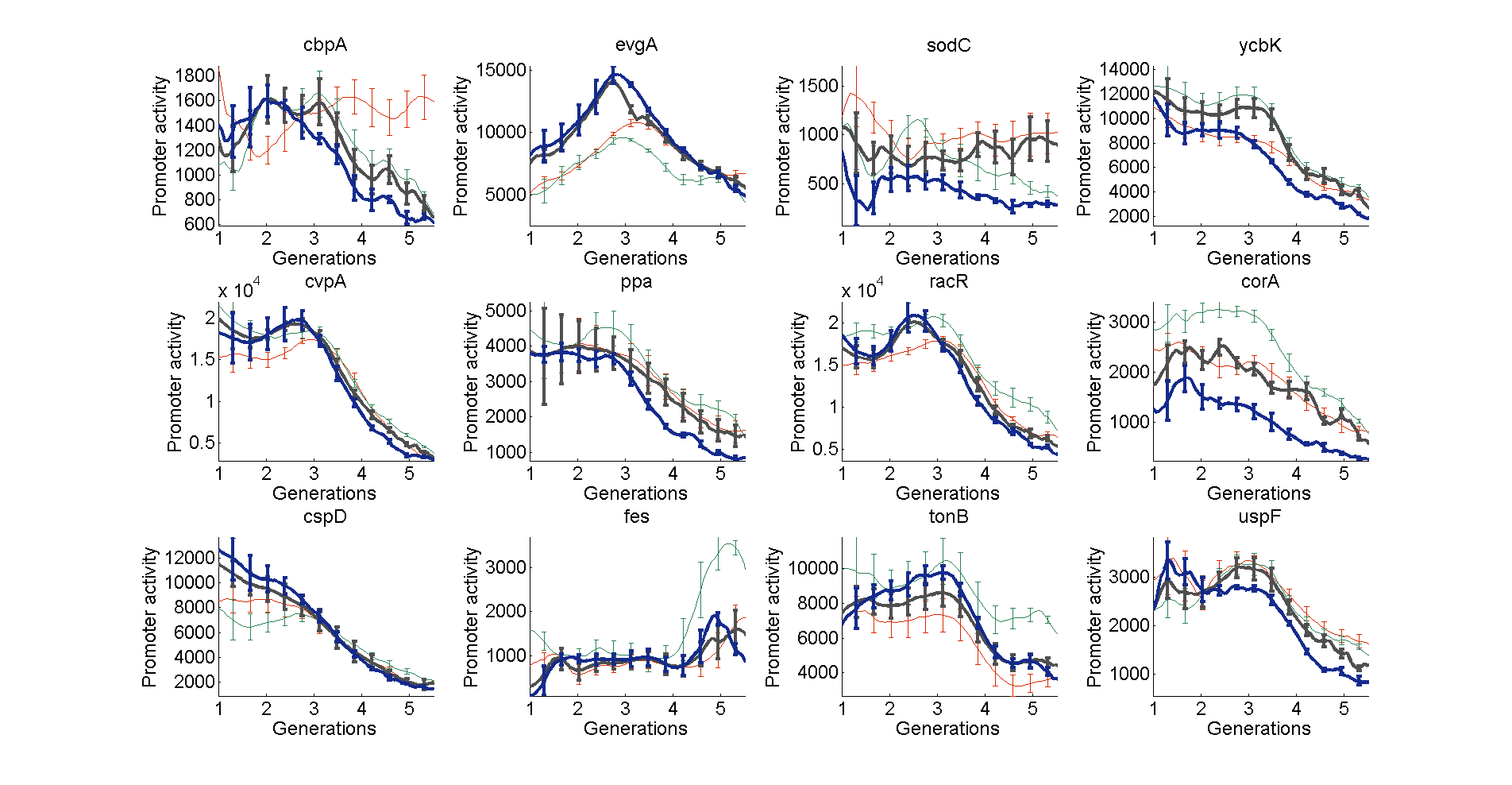


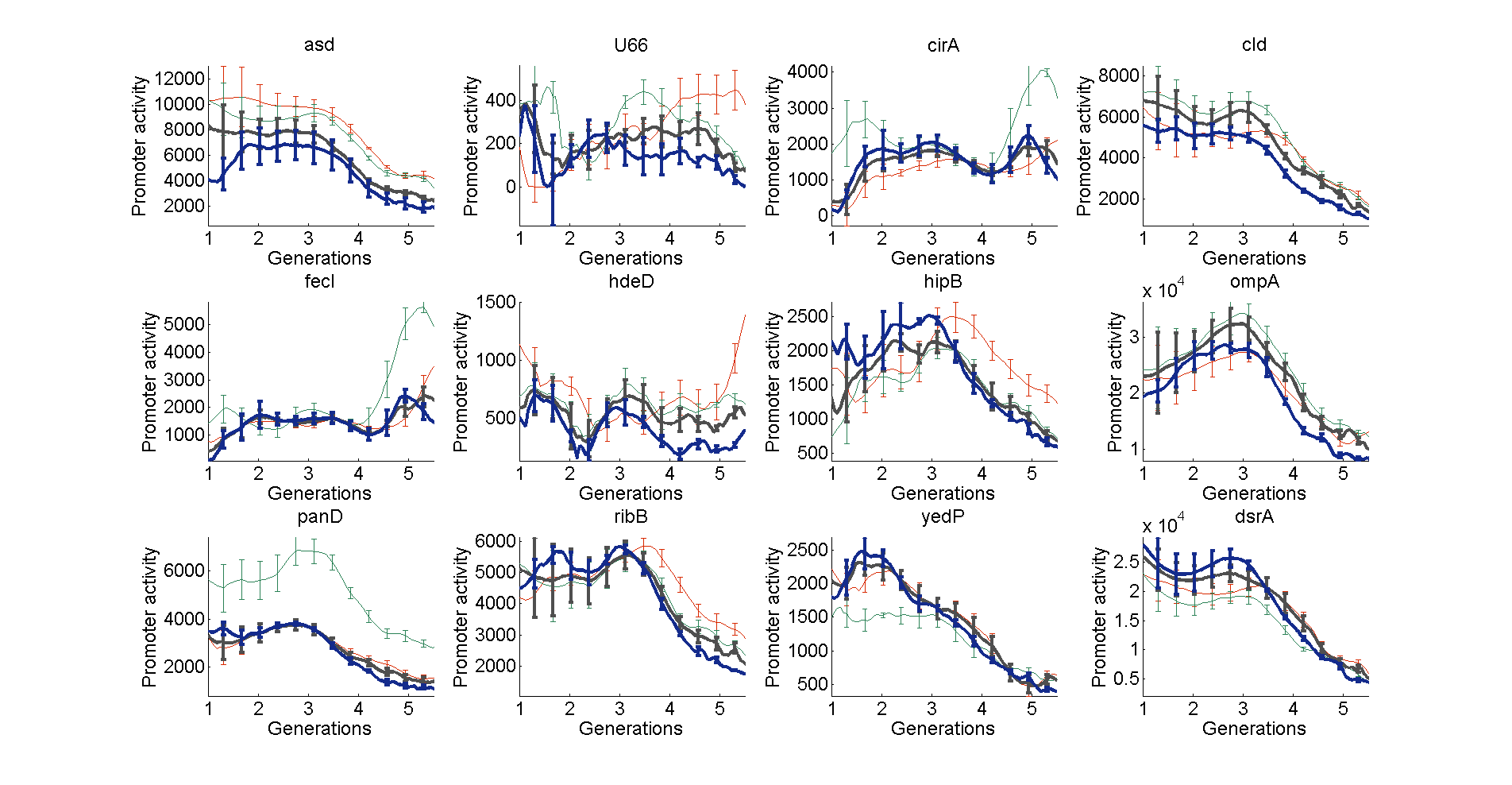


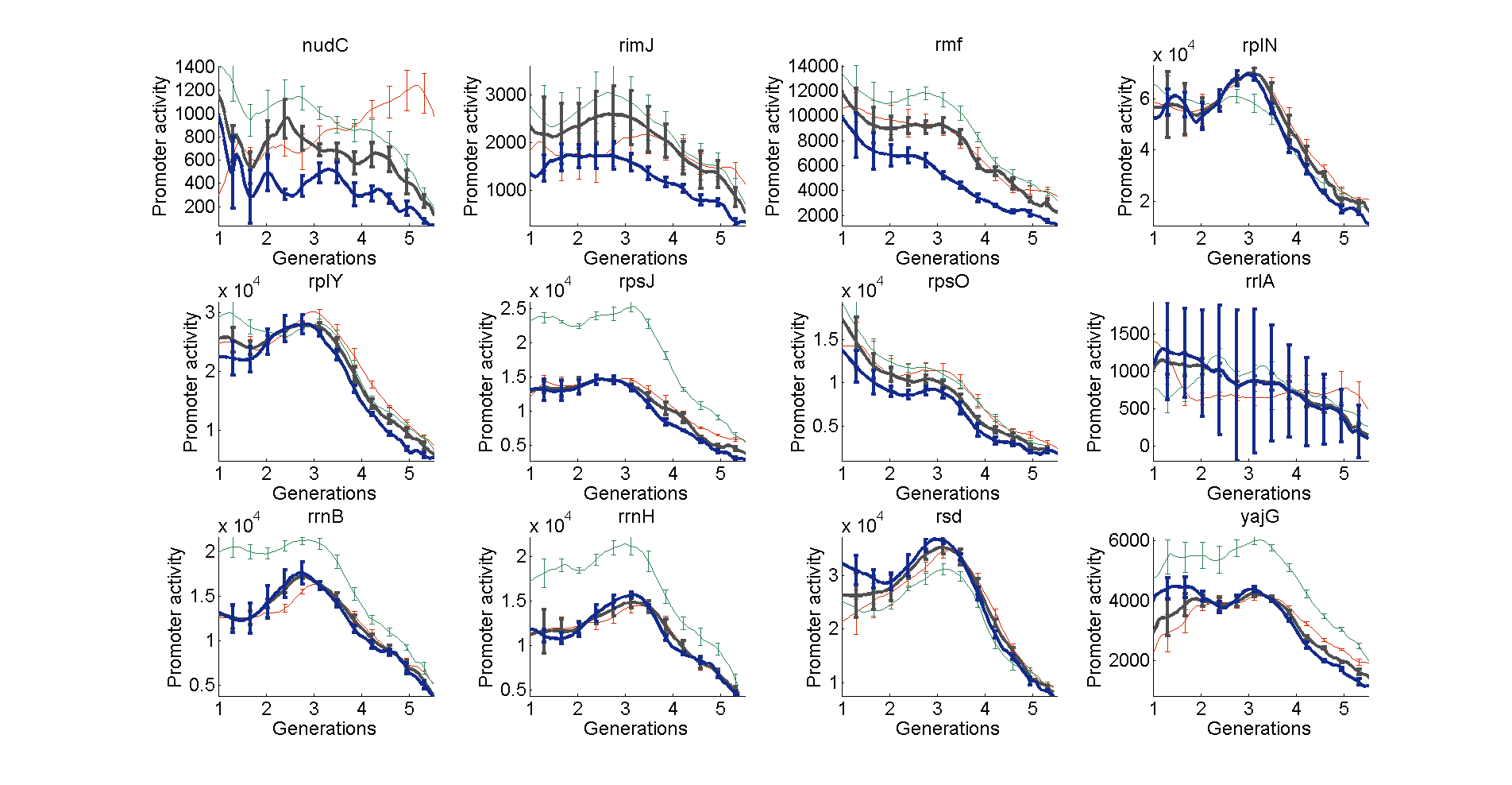


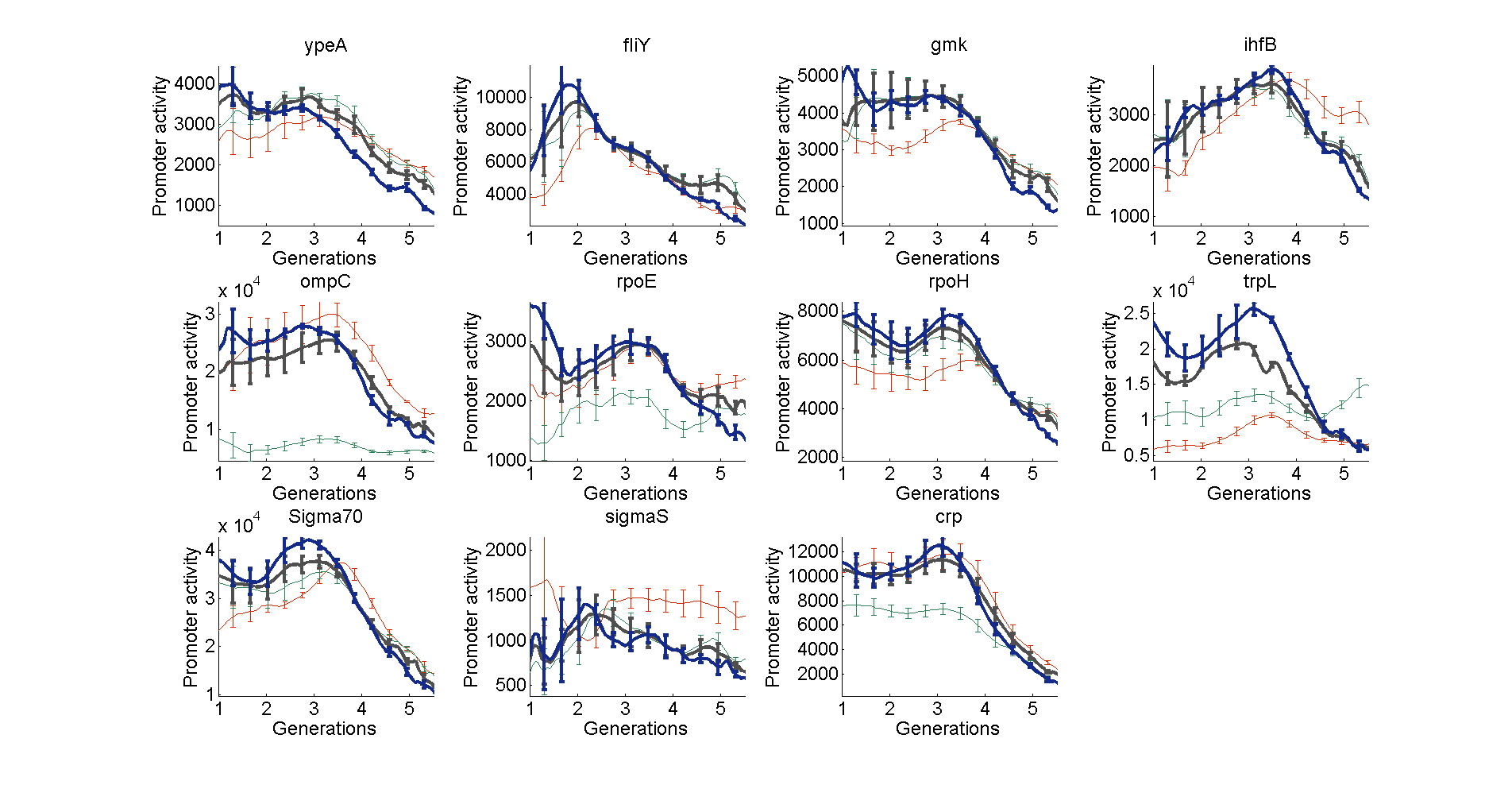


## Casamino acids, H_2_O_2_, Casamino acids + H_2_O_2_

Red – Standard medium + Casamino acids 0.05%

Green – Standard medium + H_2_O_2_ 10µM

Blue – Standard medium + Casamino acids 0.05% + H_2_O_2_ 10µM

Black – best fit linear superposition


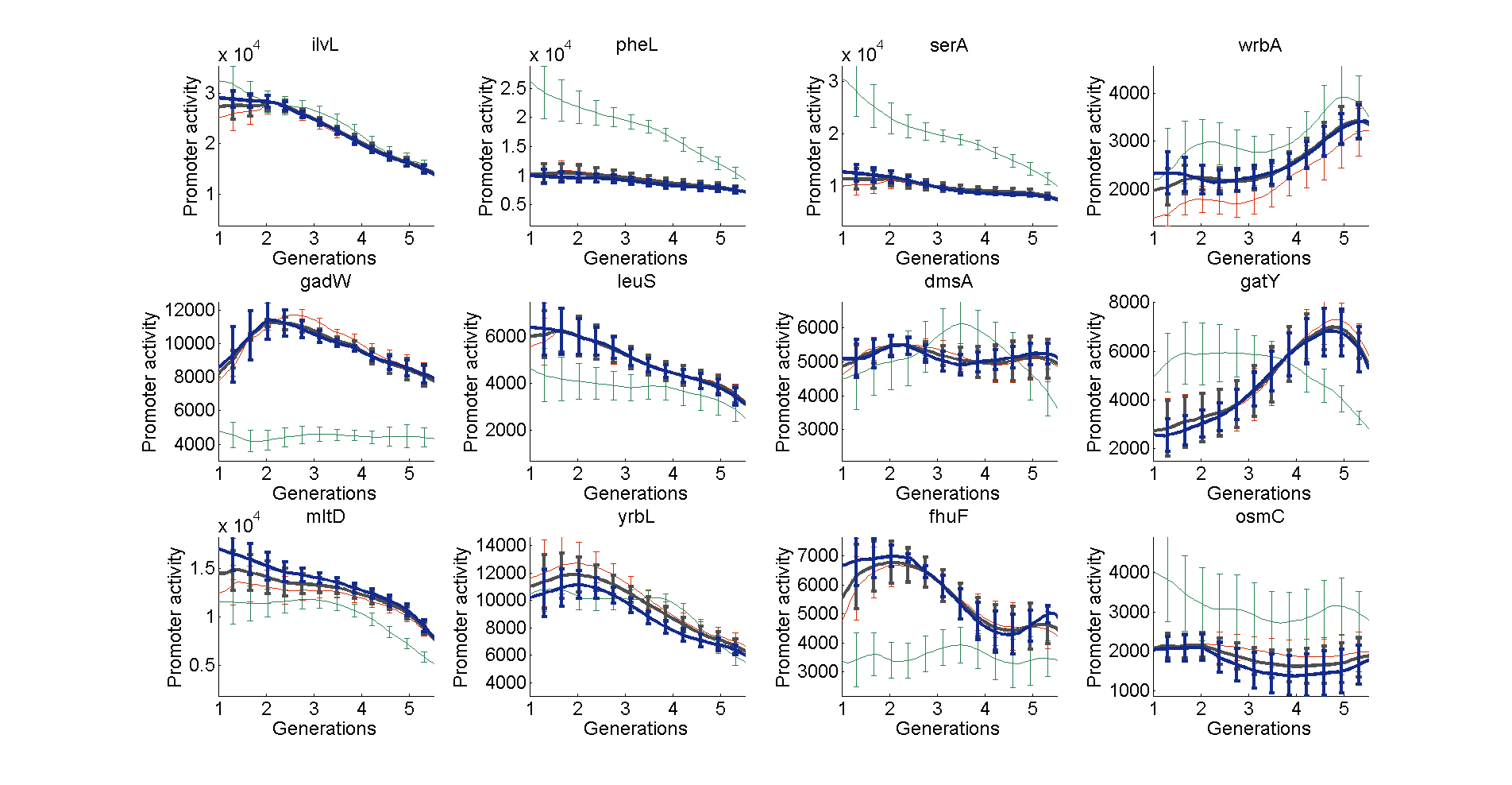


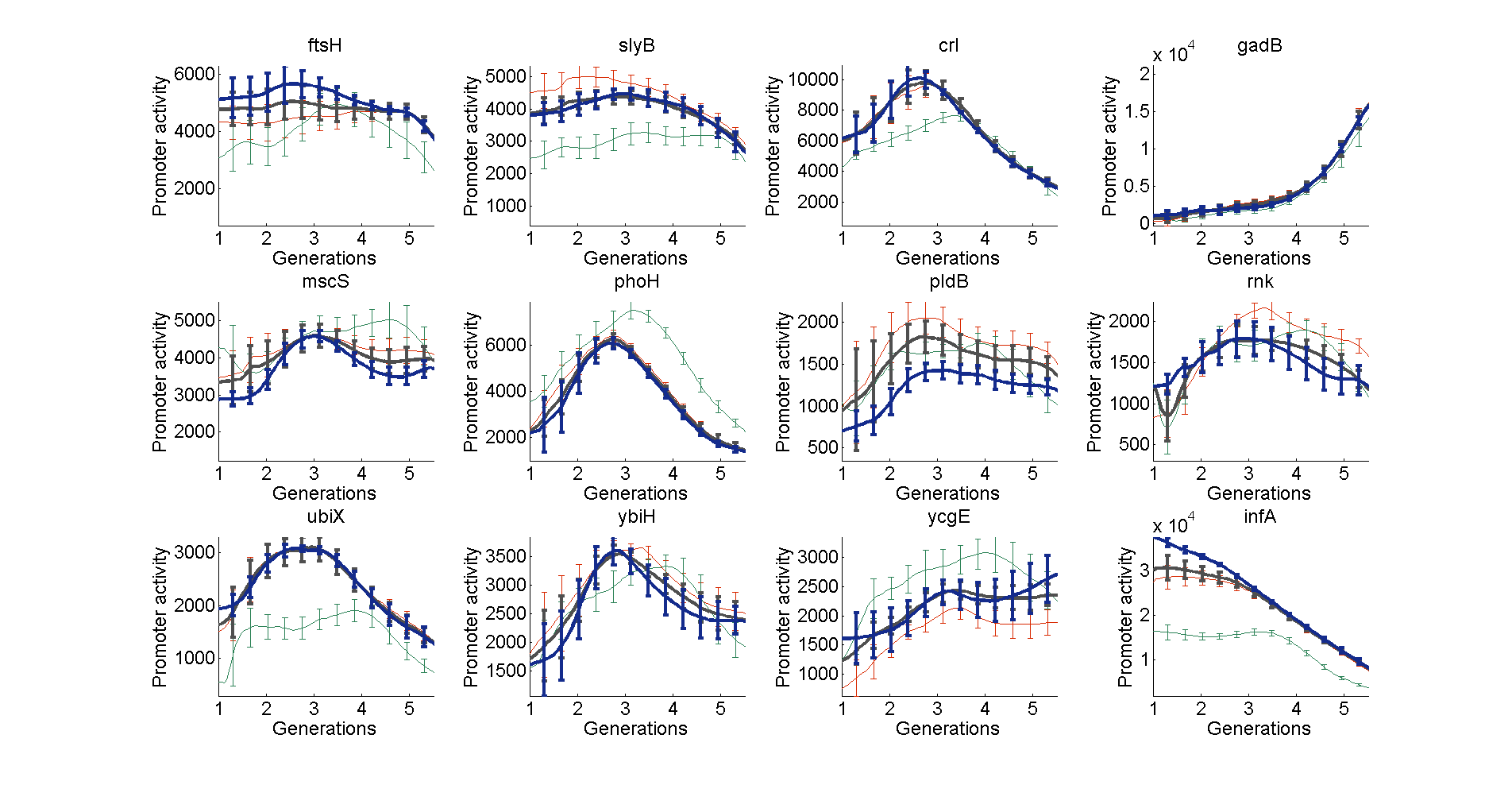


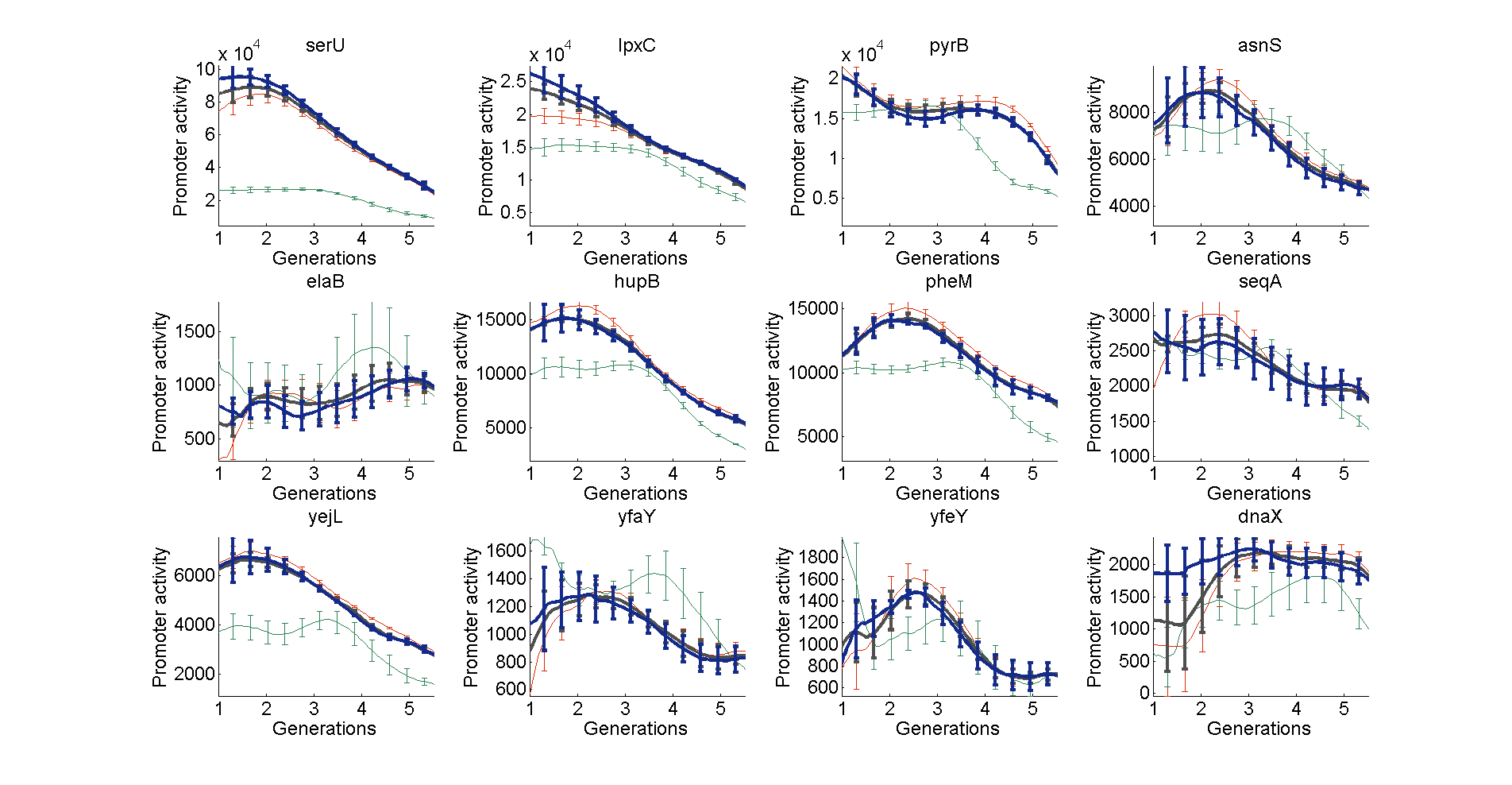


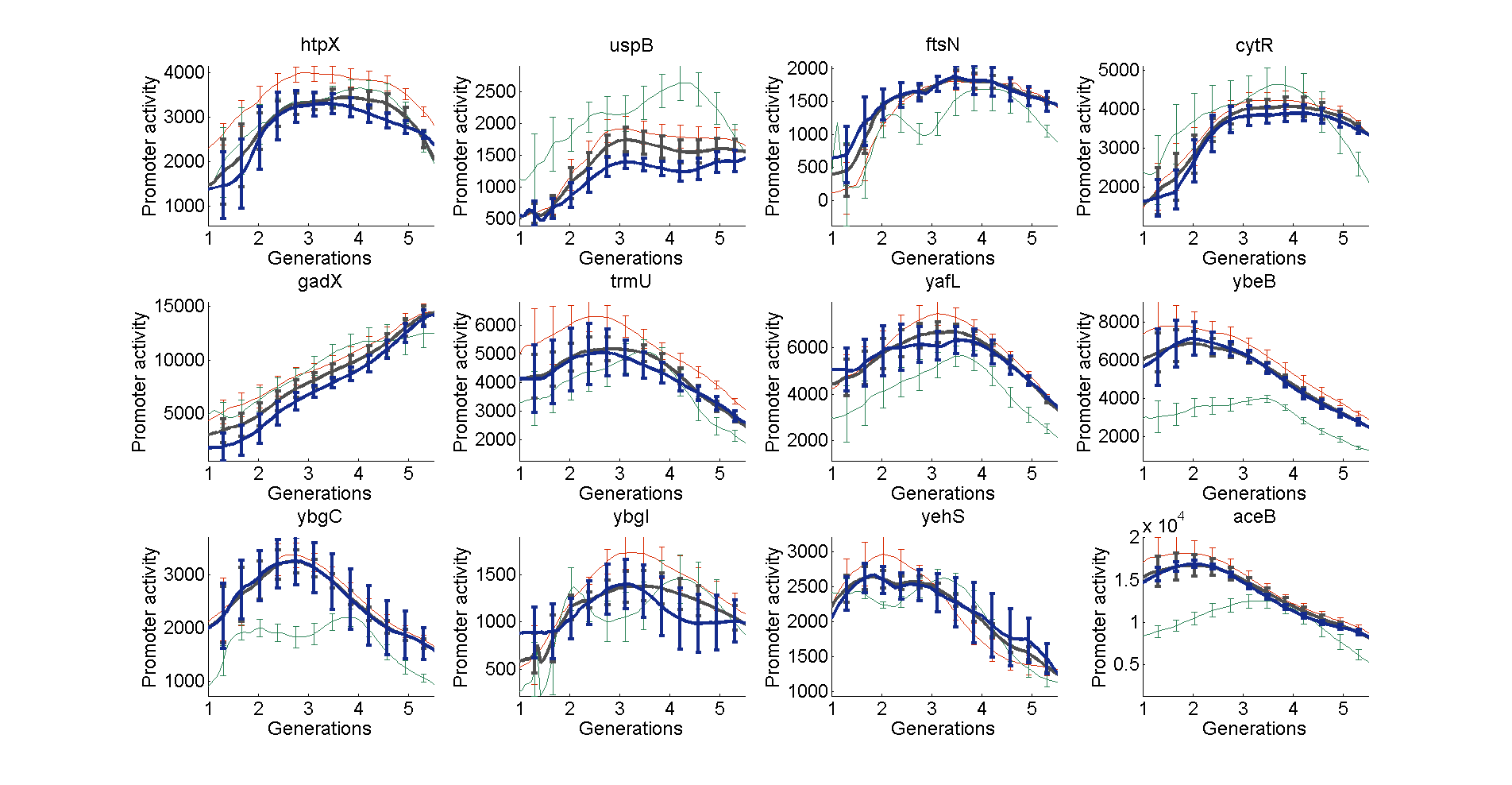


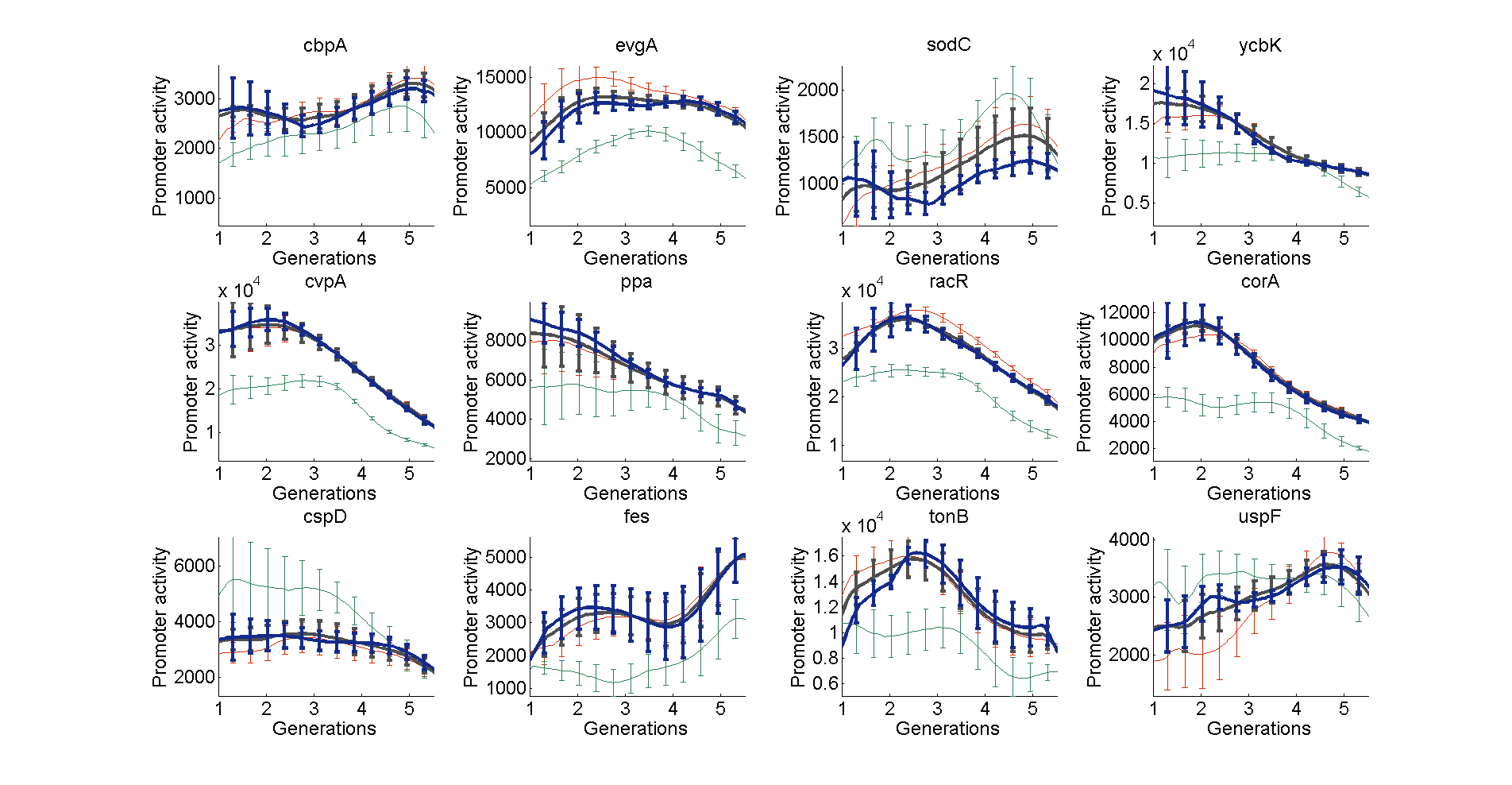


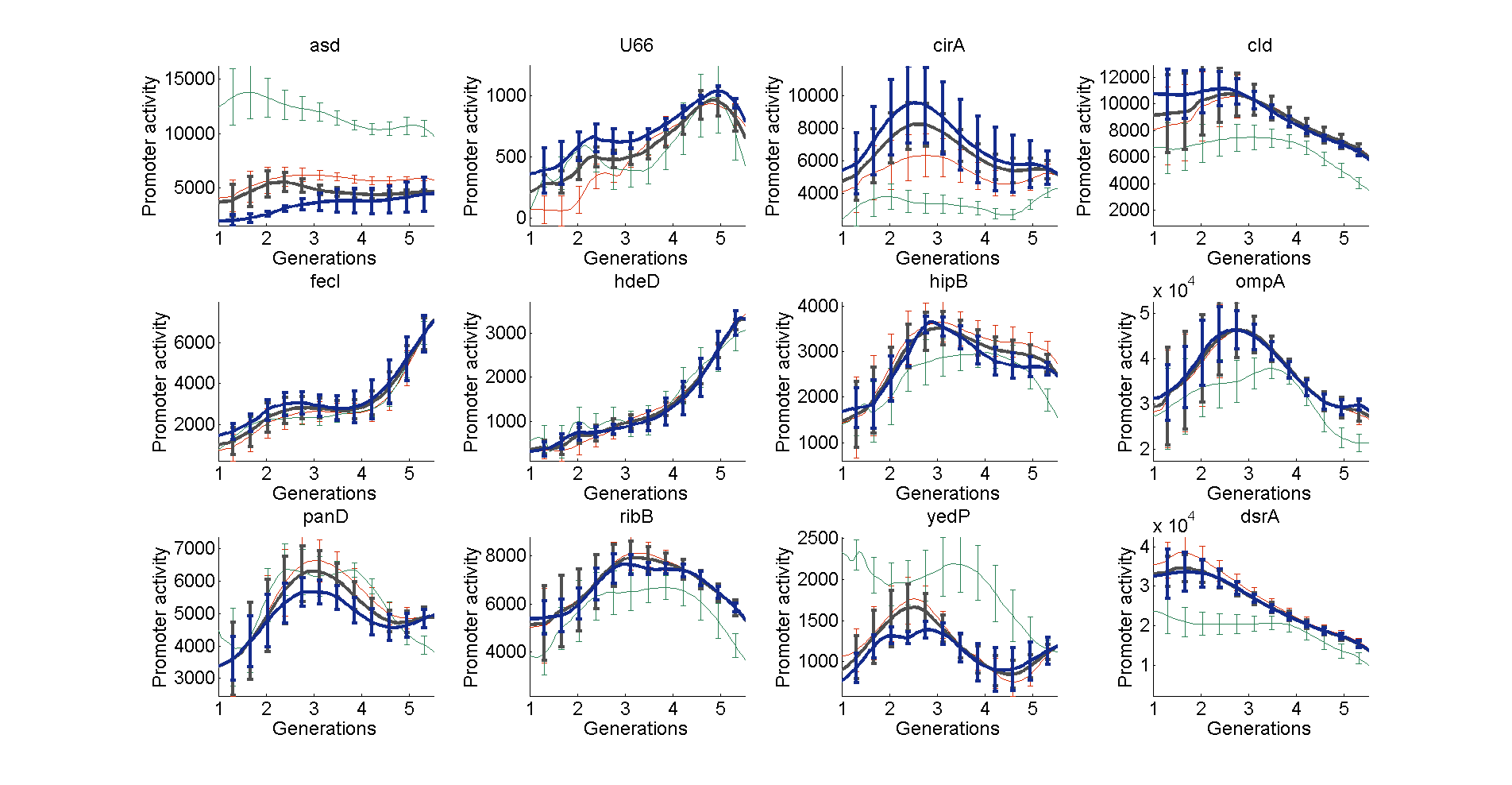


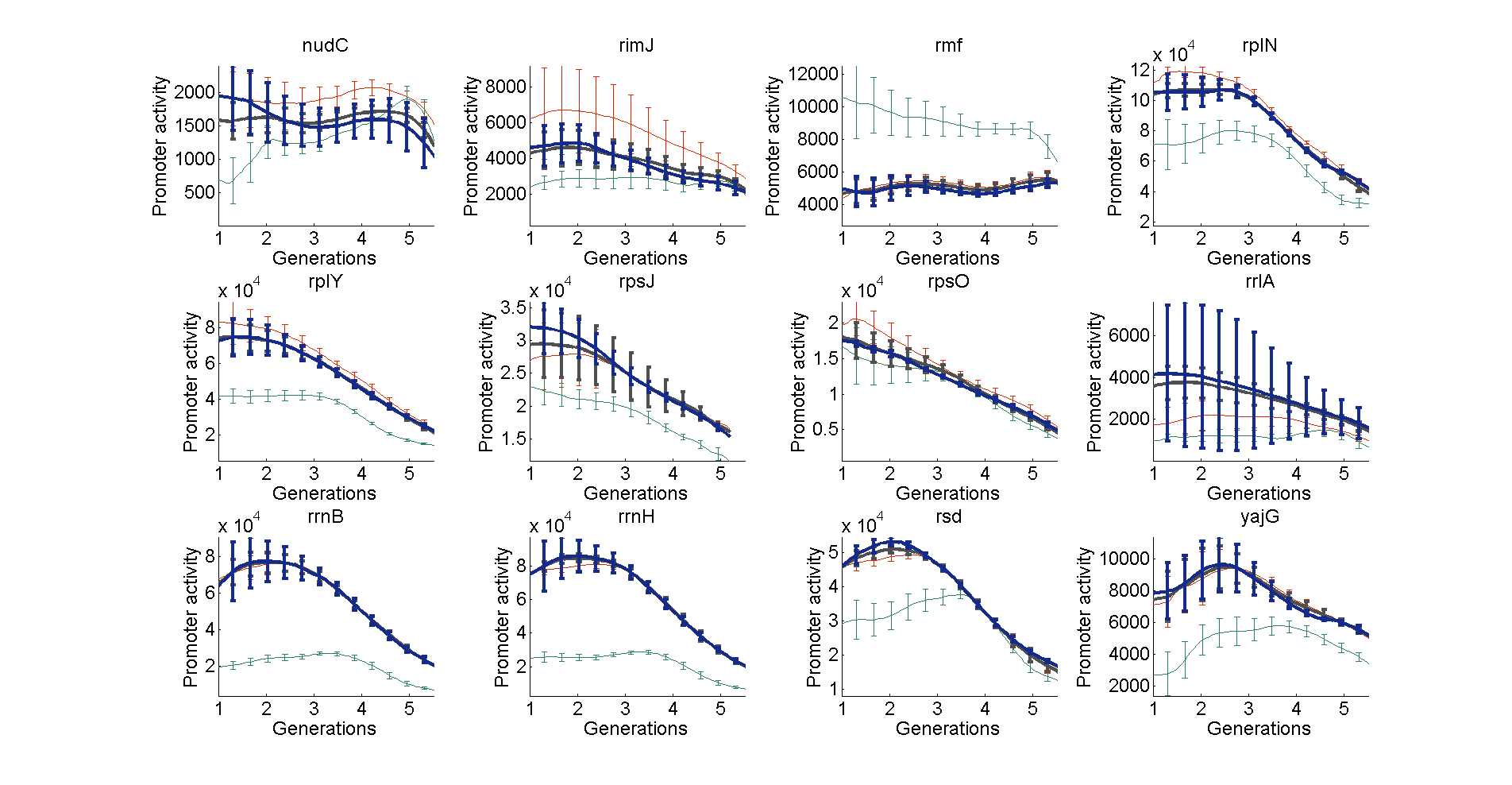


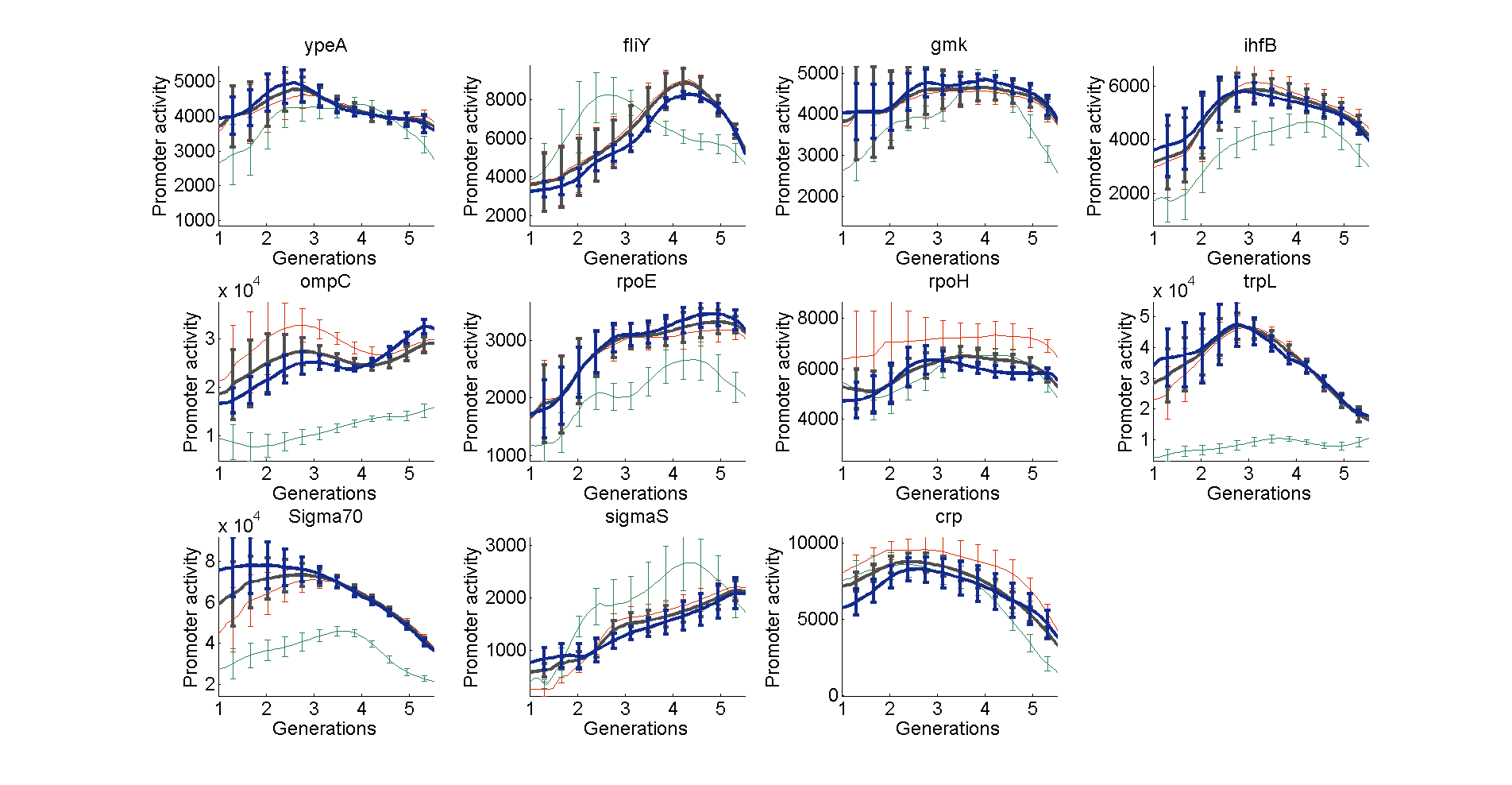


## Casamino acids, Ethanol, Casamino acids + Ethanol

Red – Standard medium + Casamino acids 0.05%

Green – Standard medium + Ethanol 3%

Blue – Standard medium + Casamino acids 0.05% + Ethanol 3%

Black – best fit linear superposition


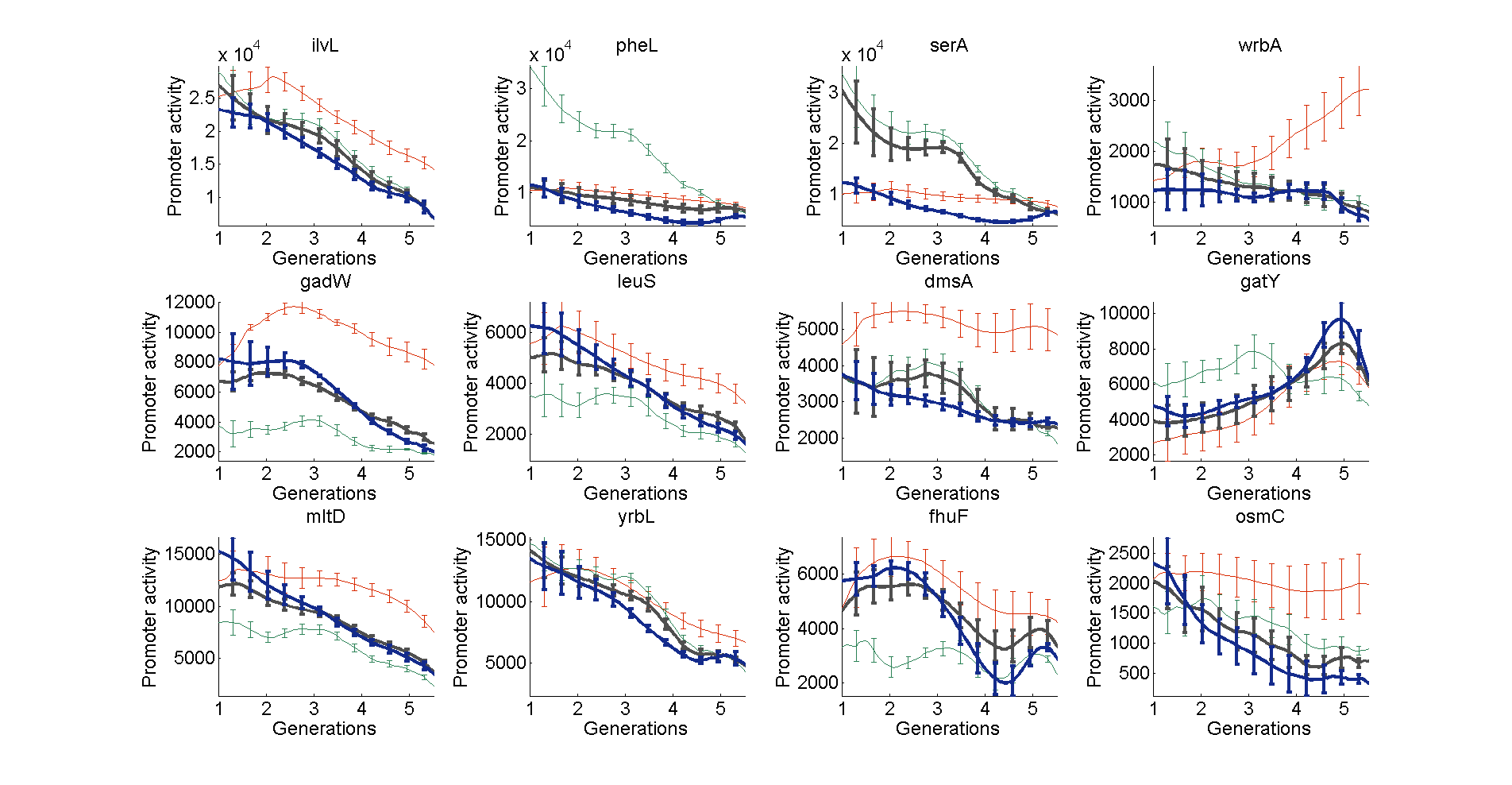


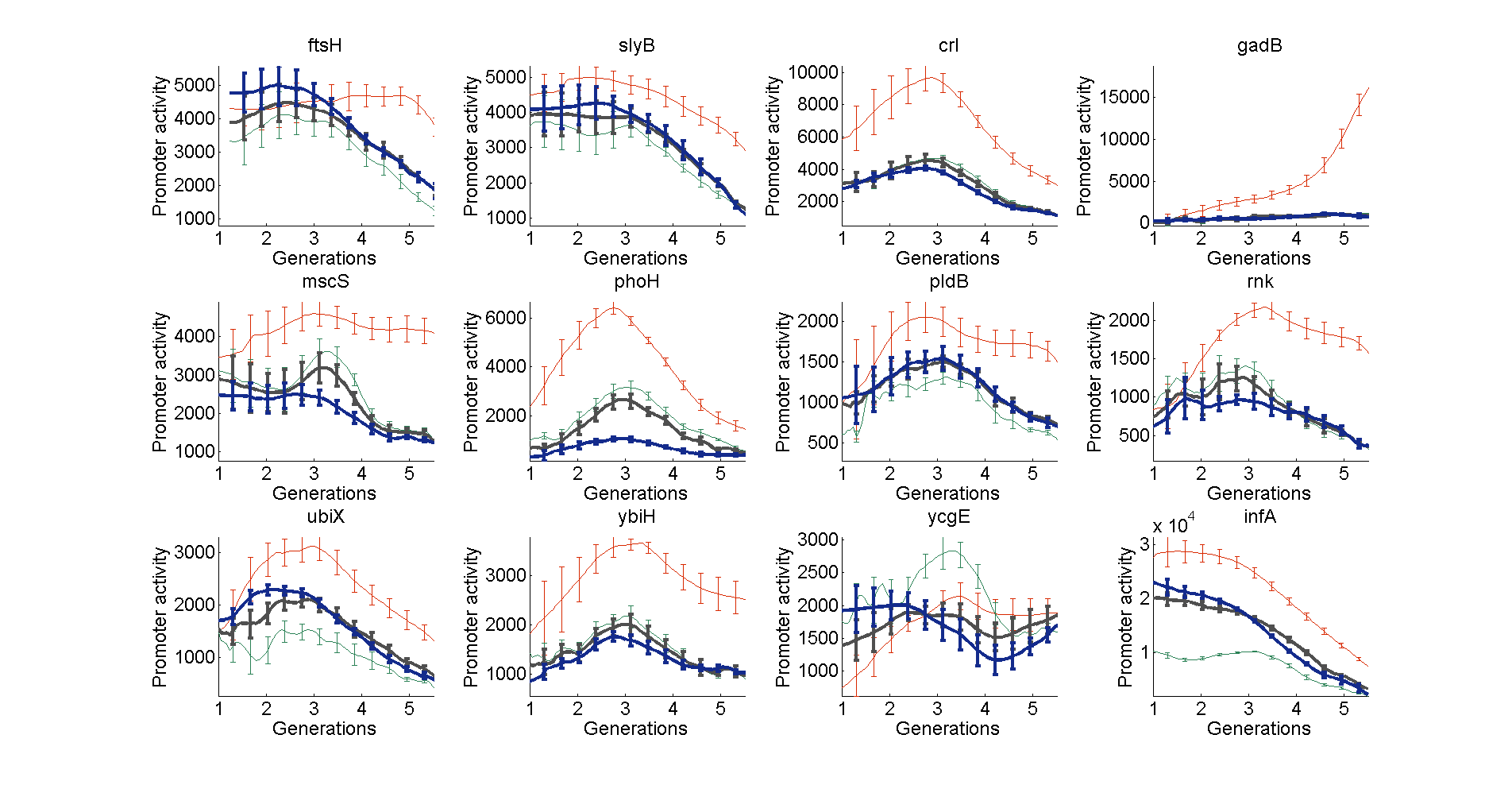


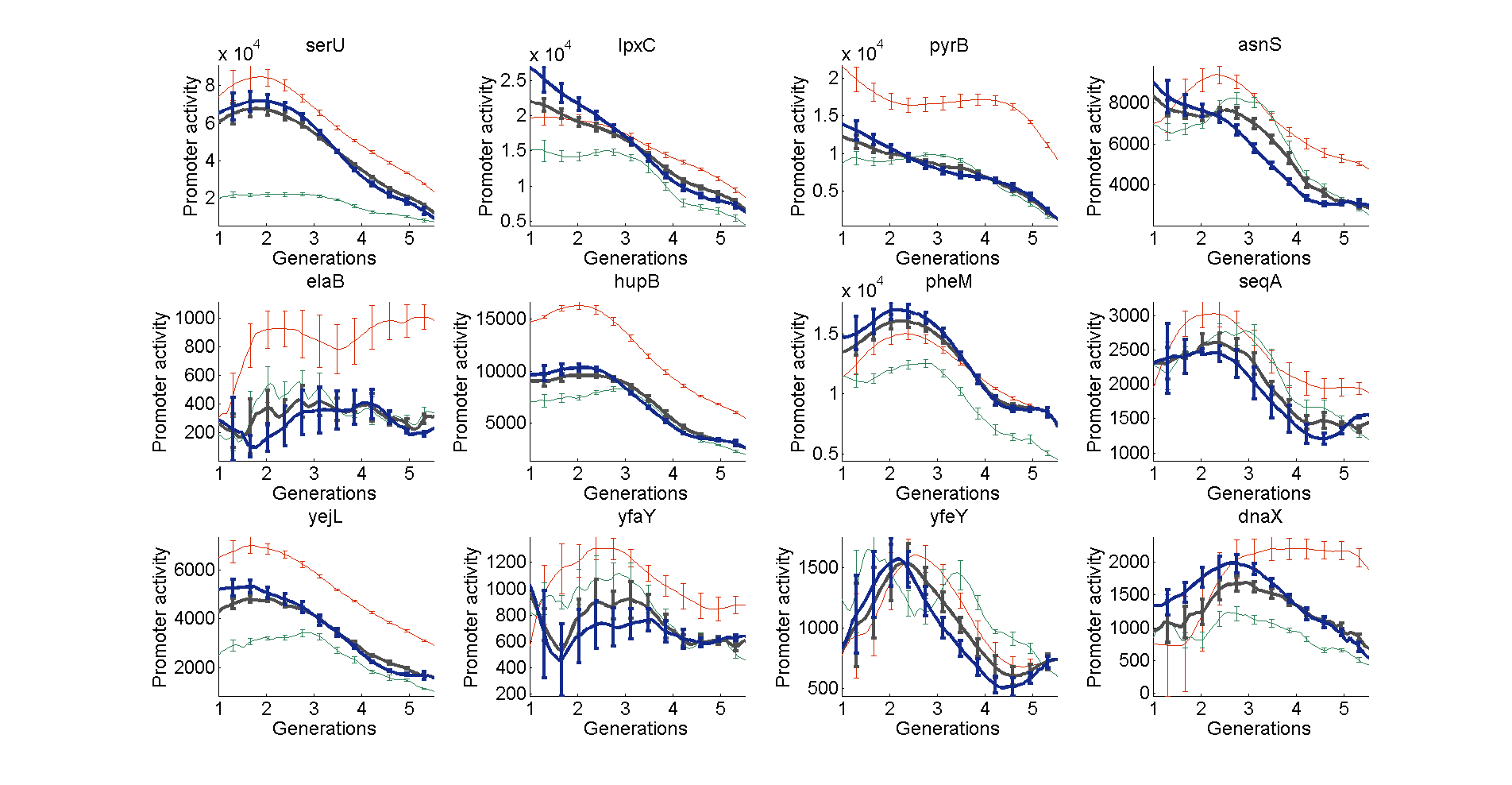


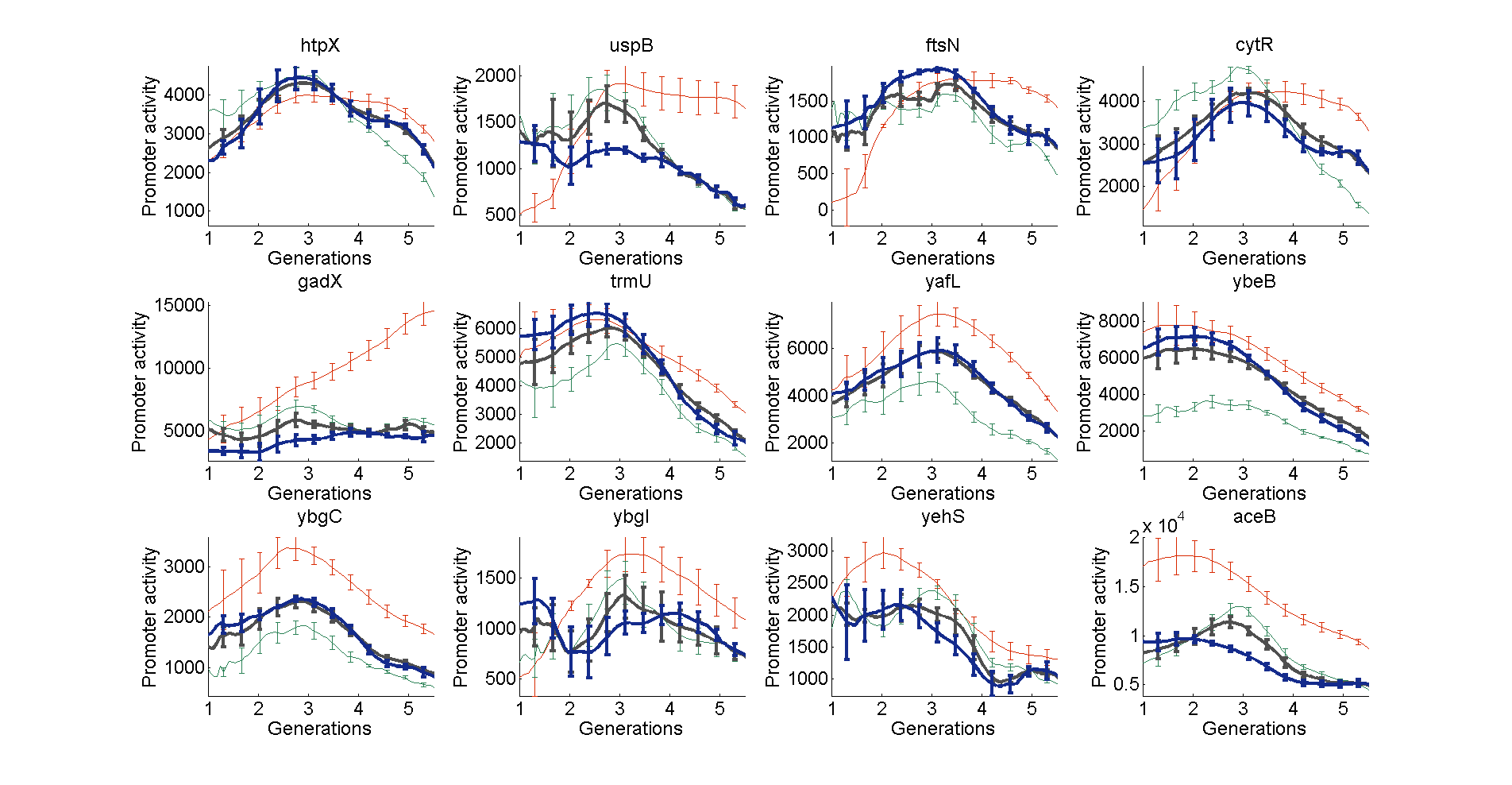


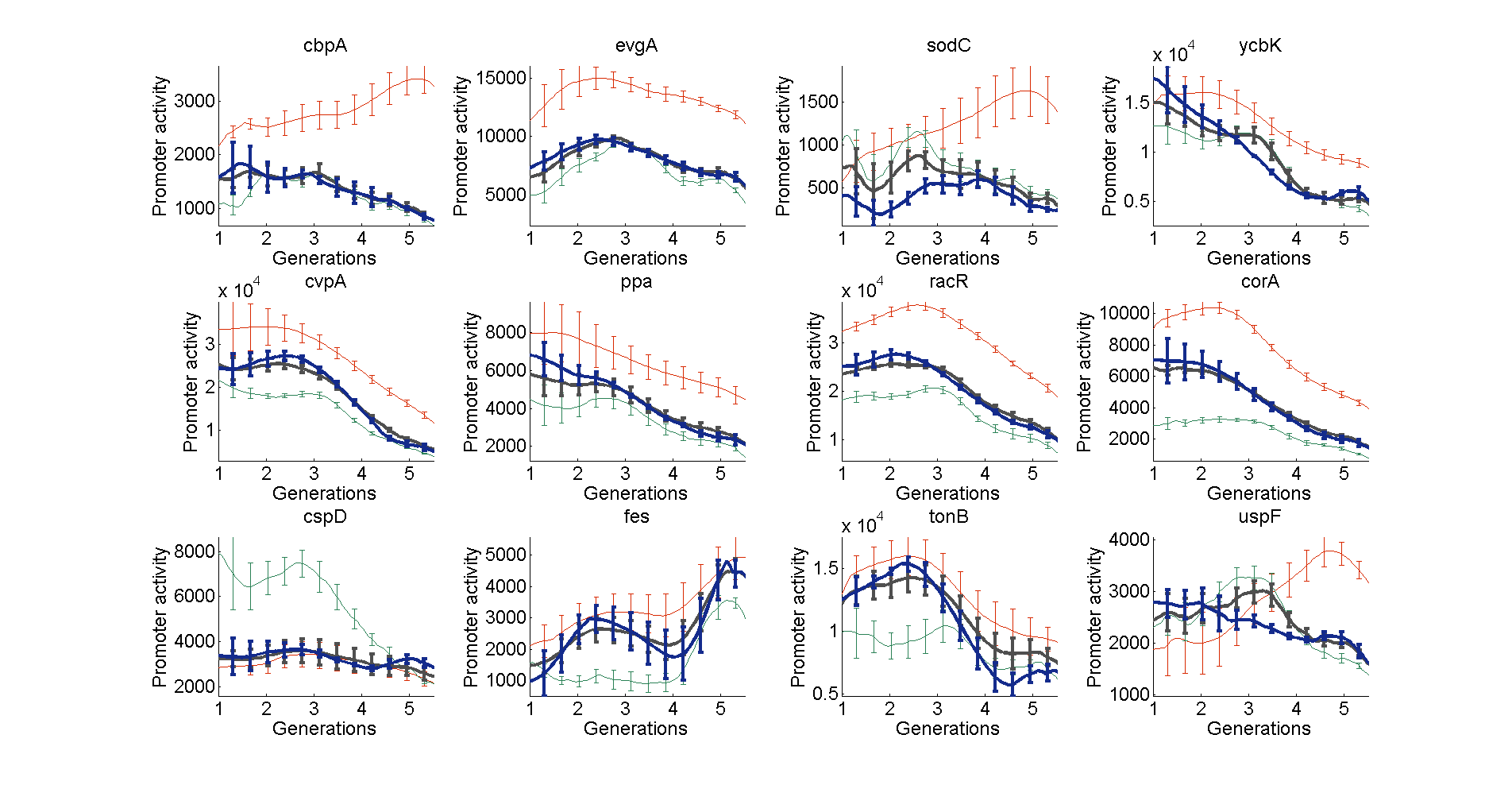


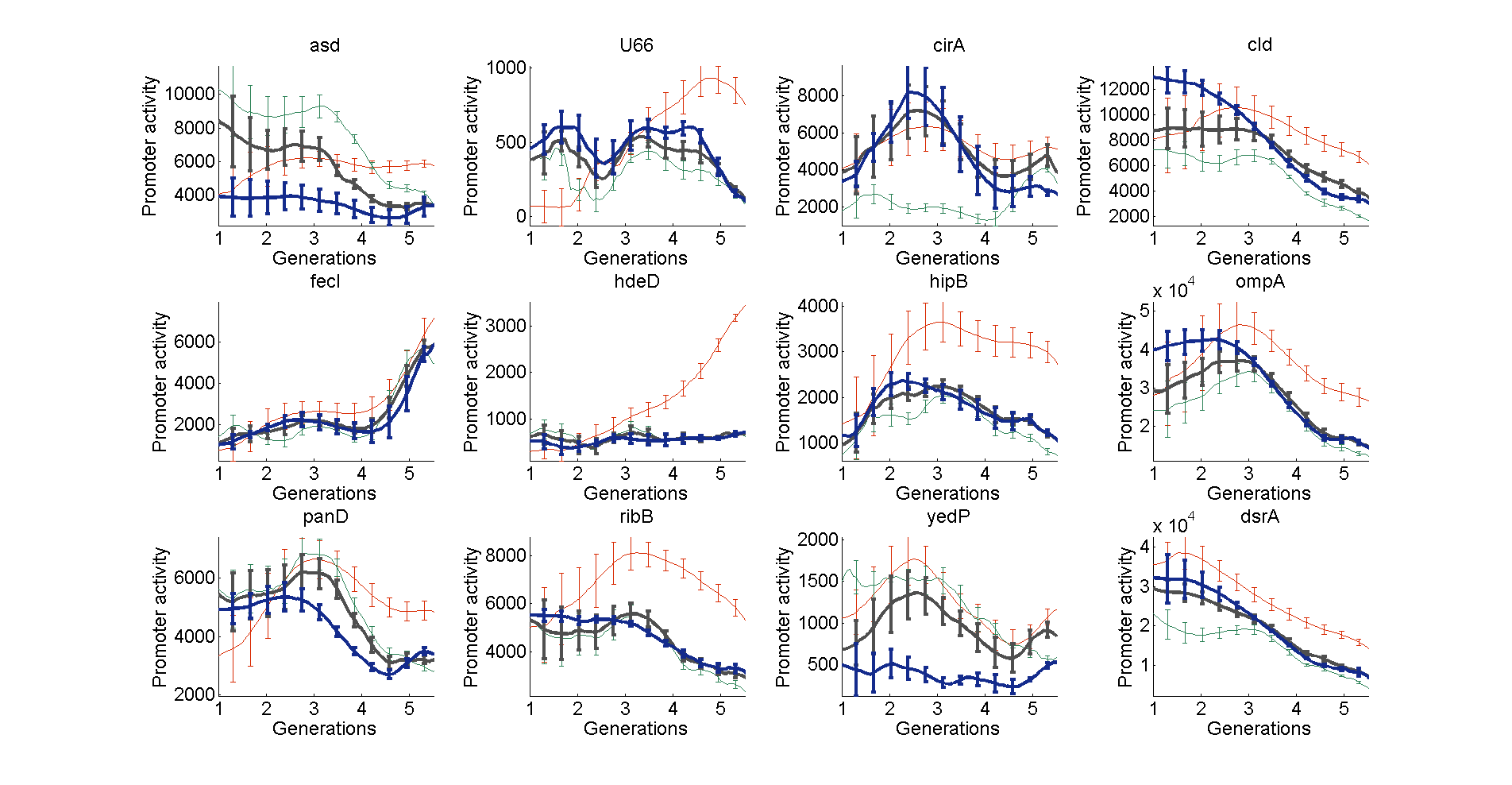


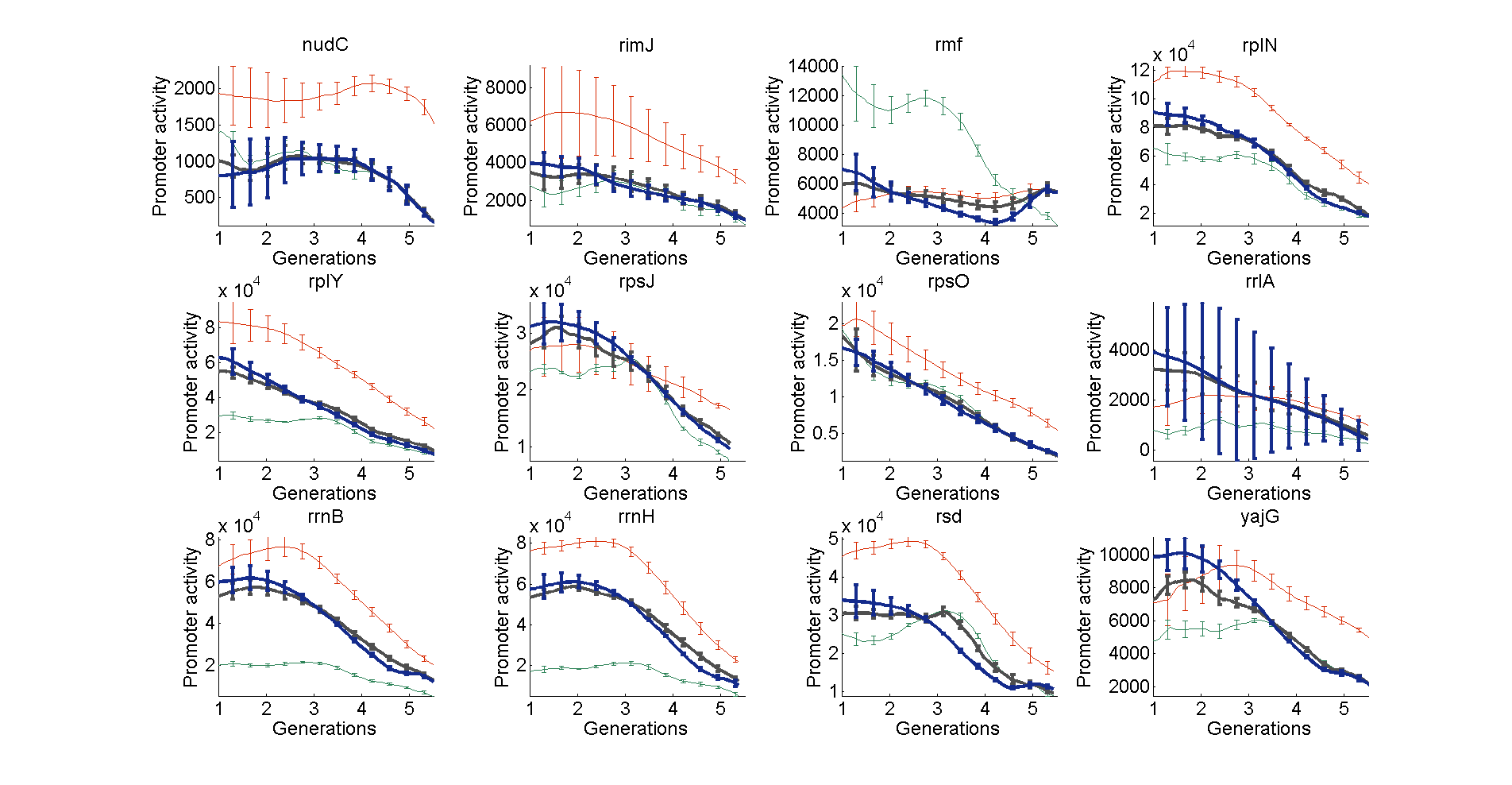


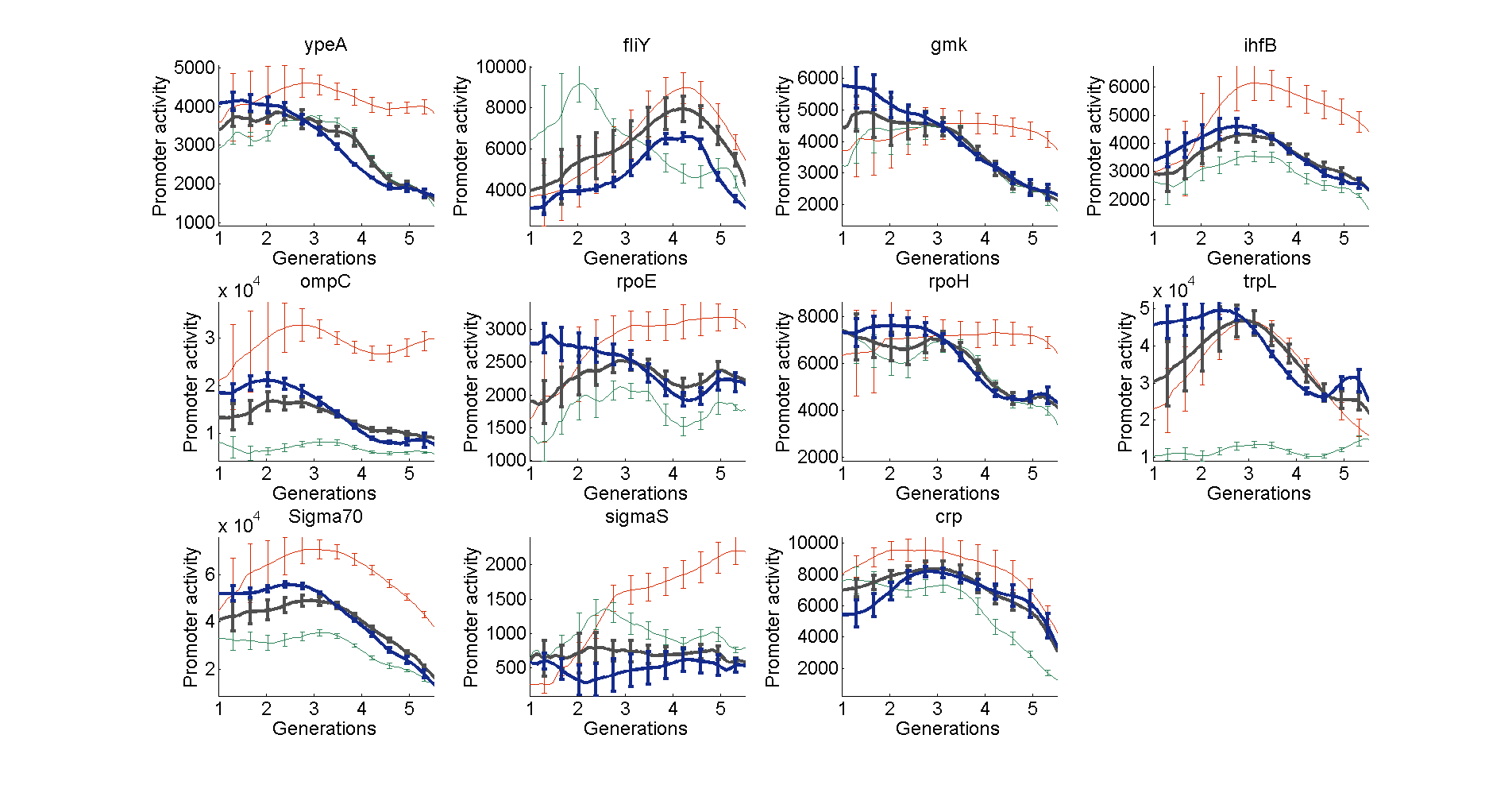


## H_2_O_2_, Ethanol, H_2_O_2_ + Ethanol

Red – Standard medium + H_2_O_2_ 10µM

Green – Standard medium + Ethanol 3%

Blue – Standard medium + H_2_O_2_ 10µM + Ethanol 3%

Black – best fit linear superposition


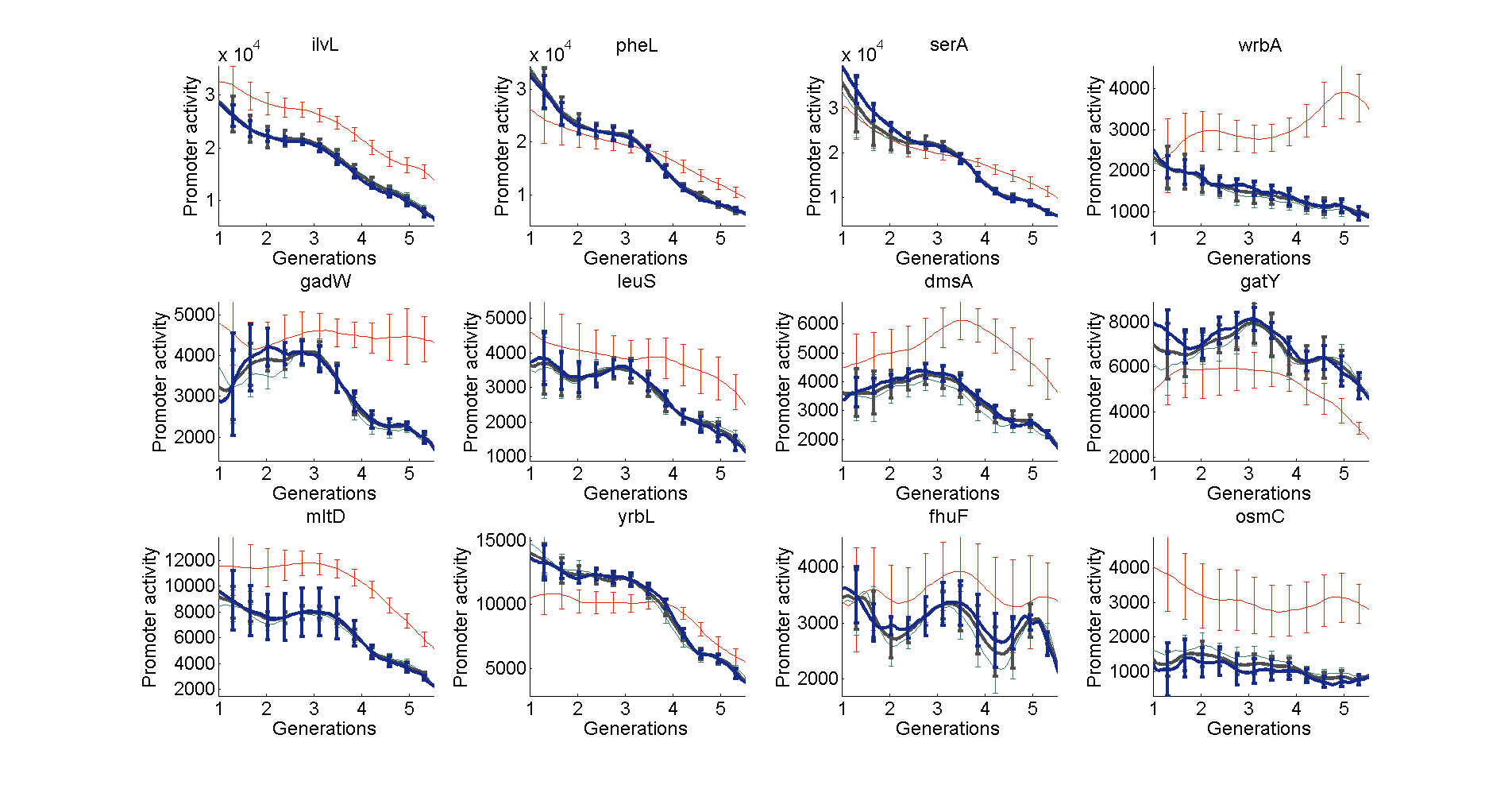


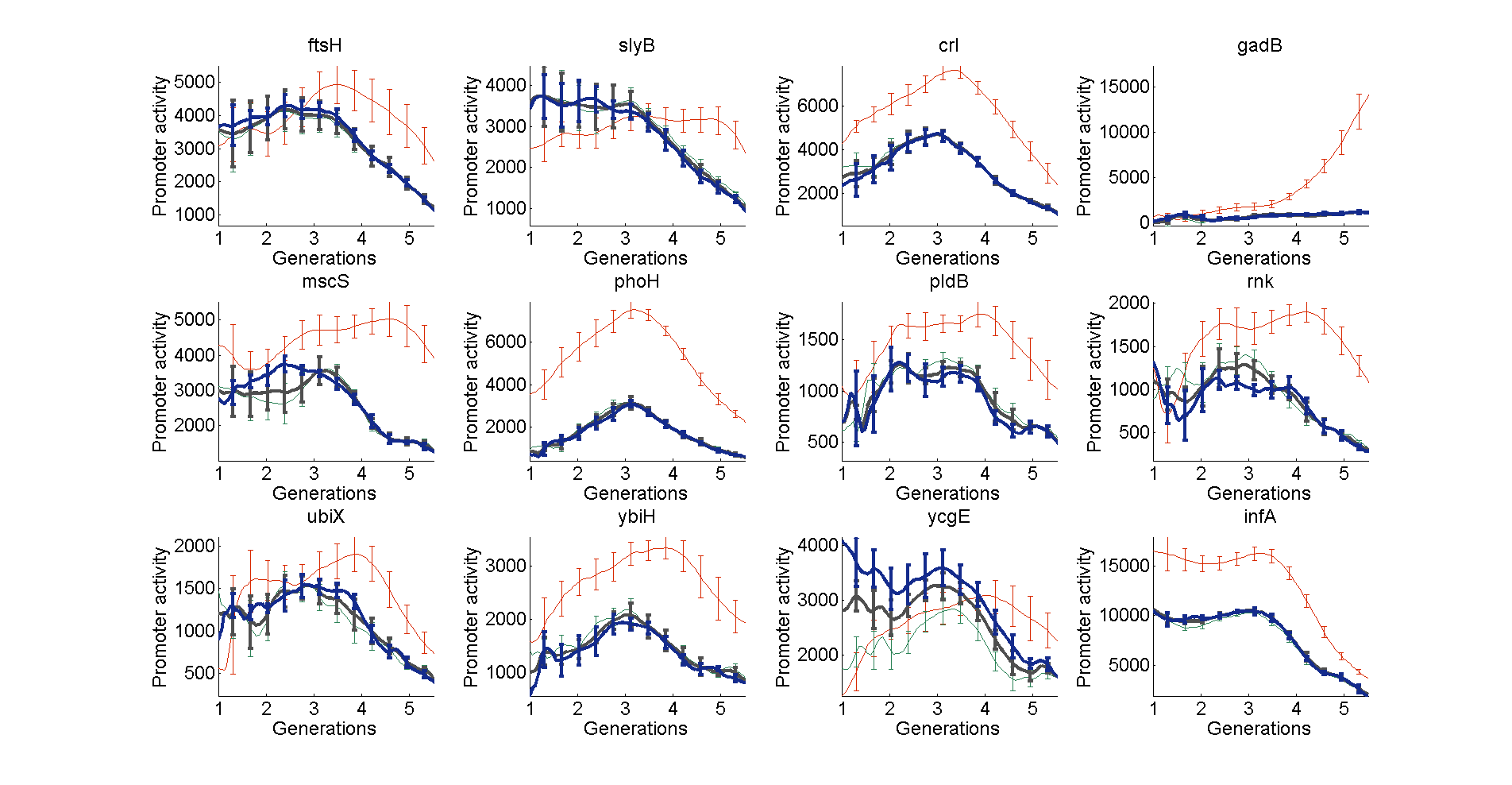


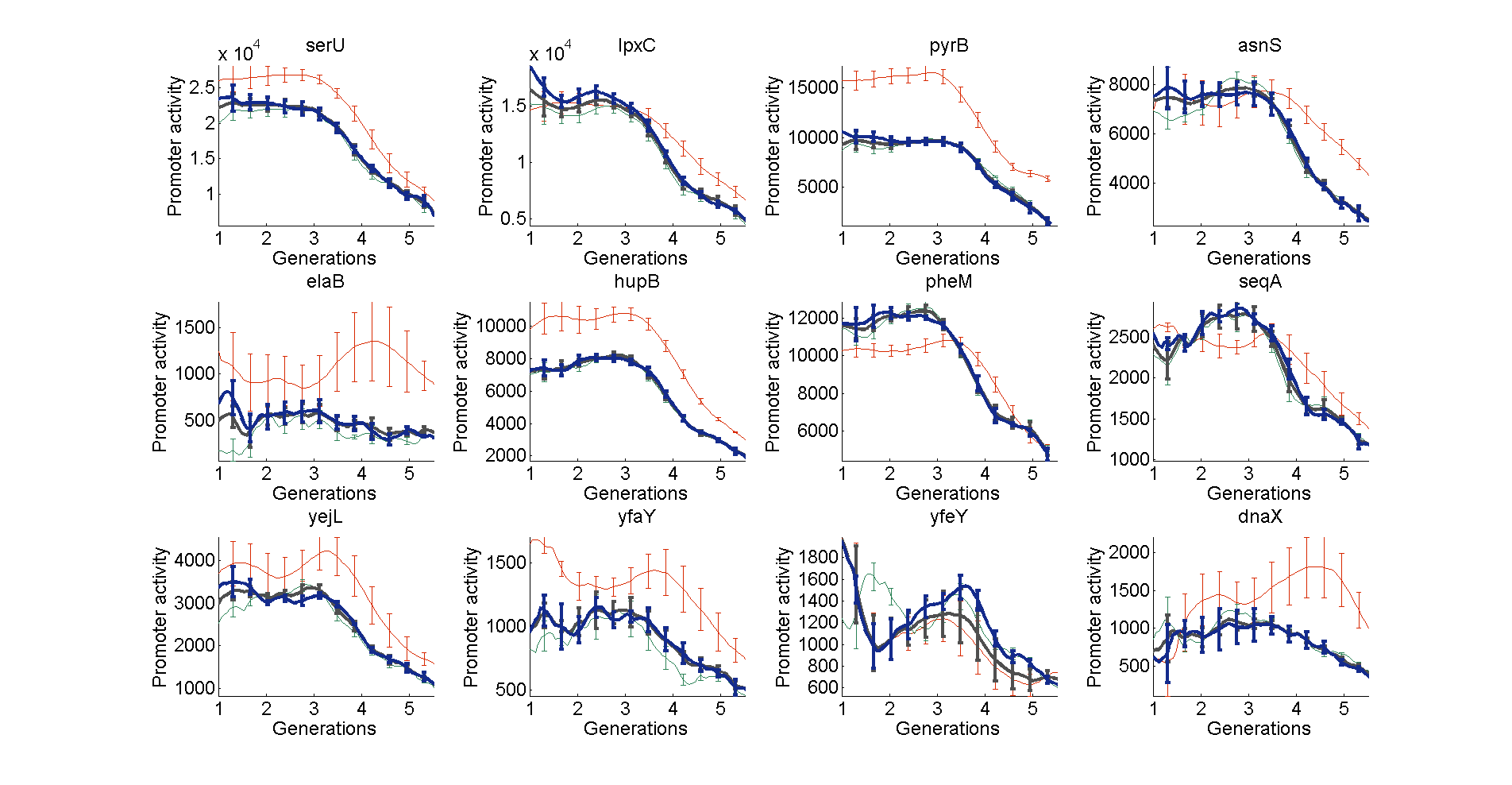


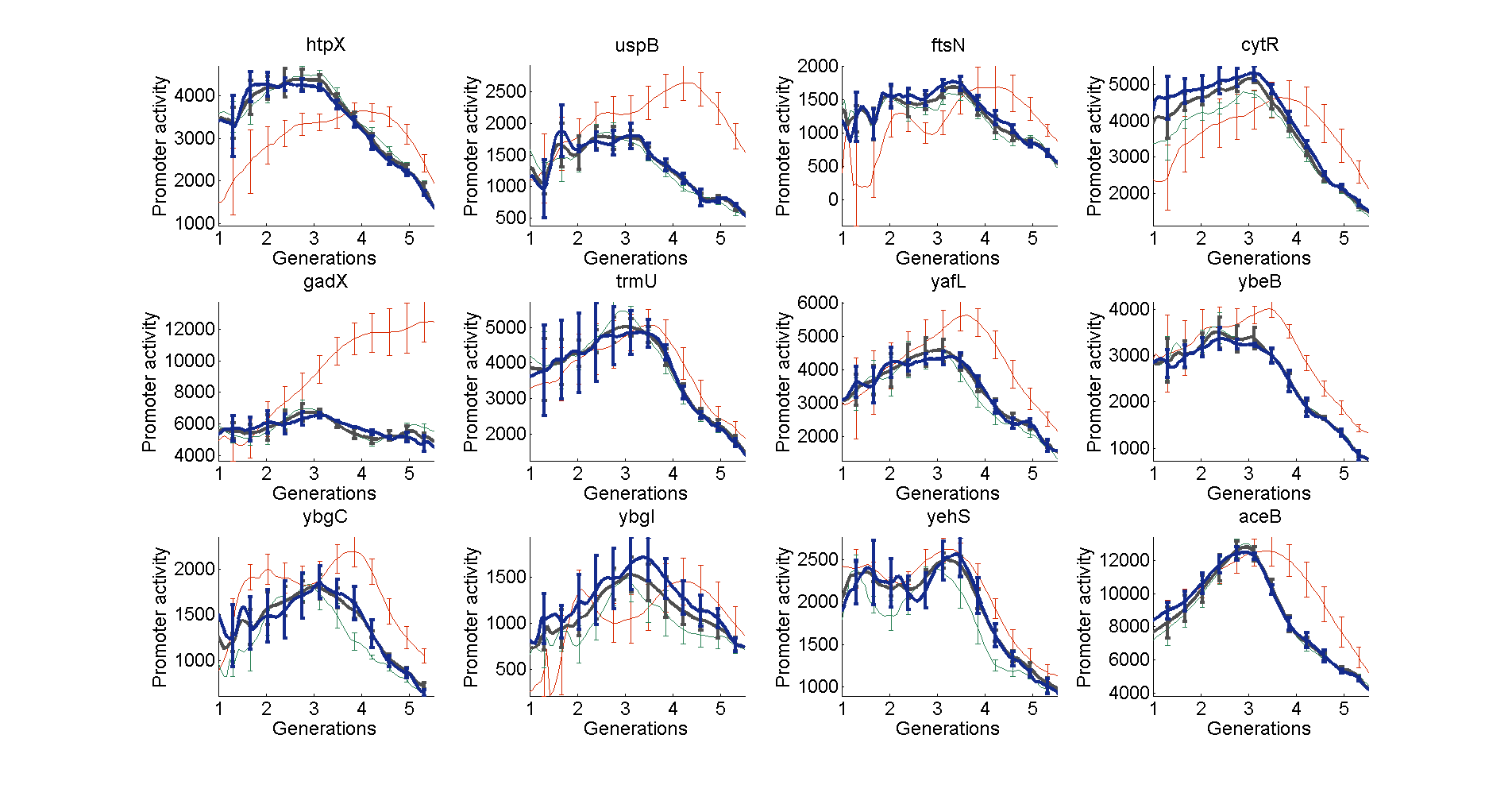


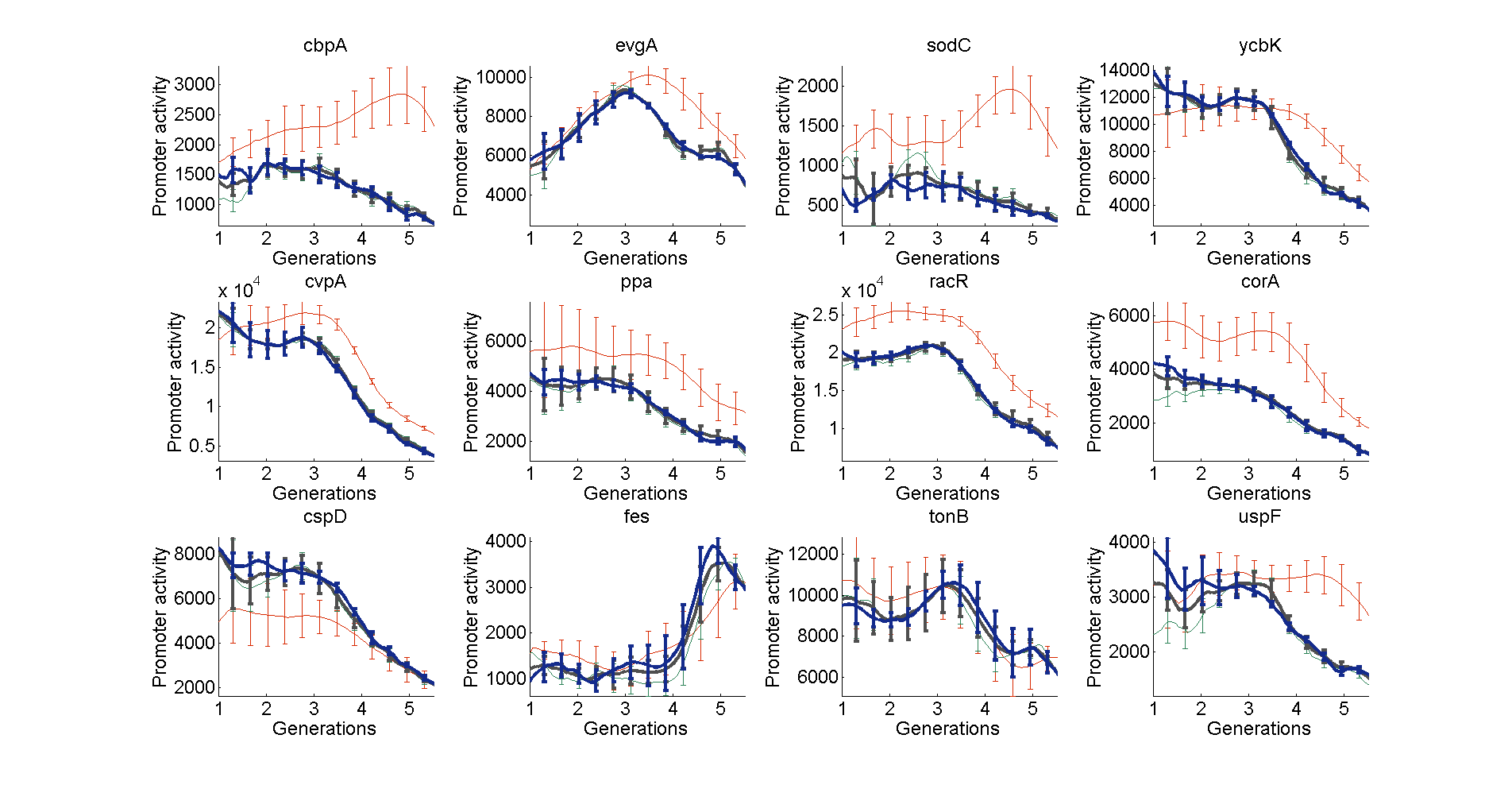


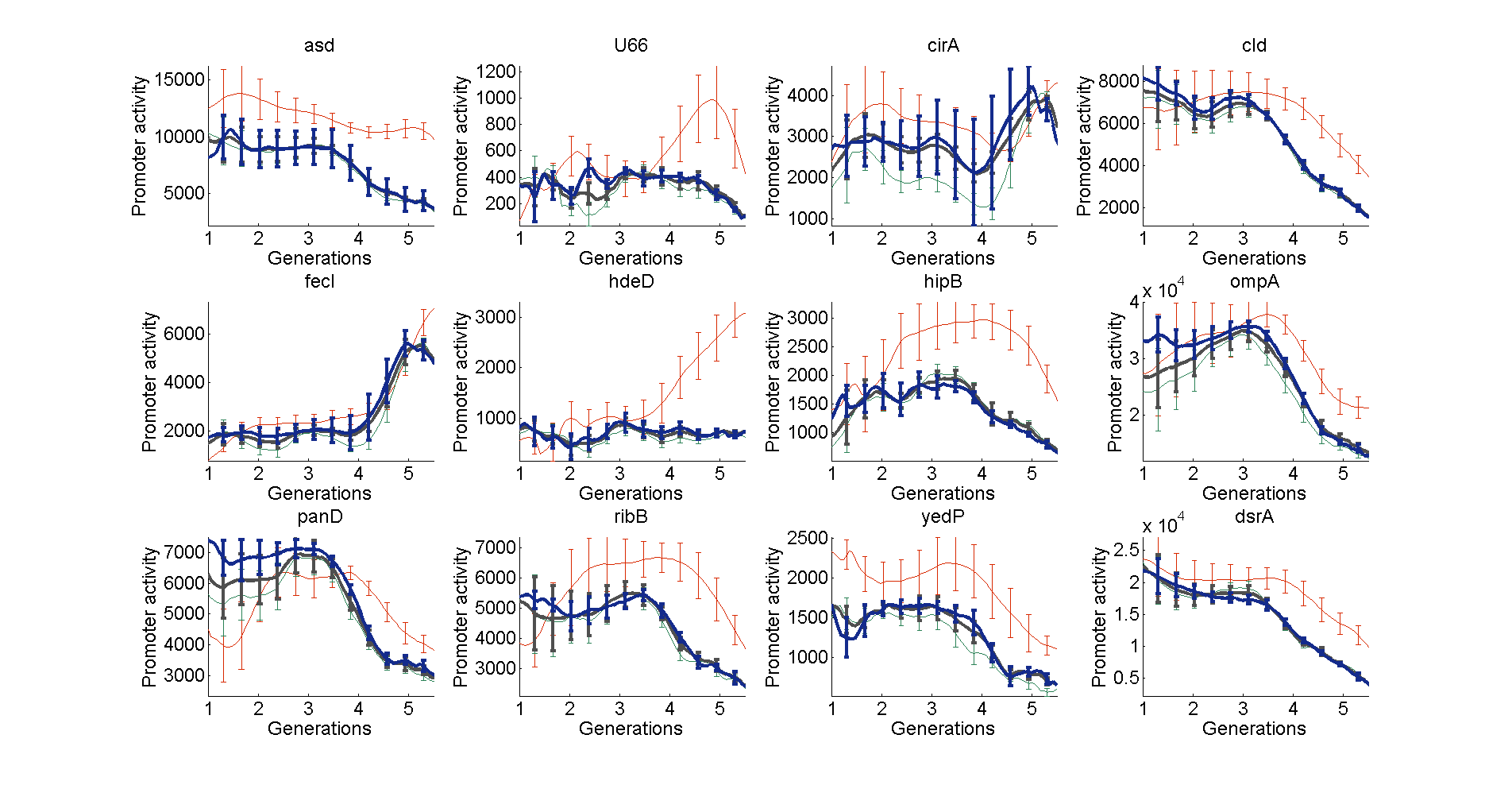


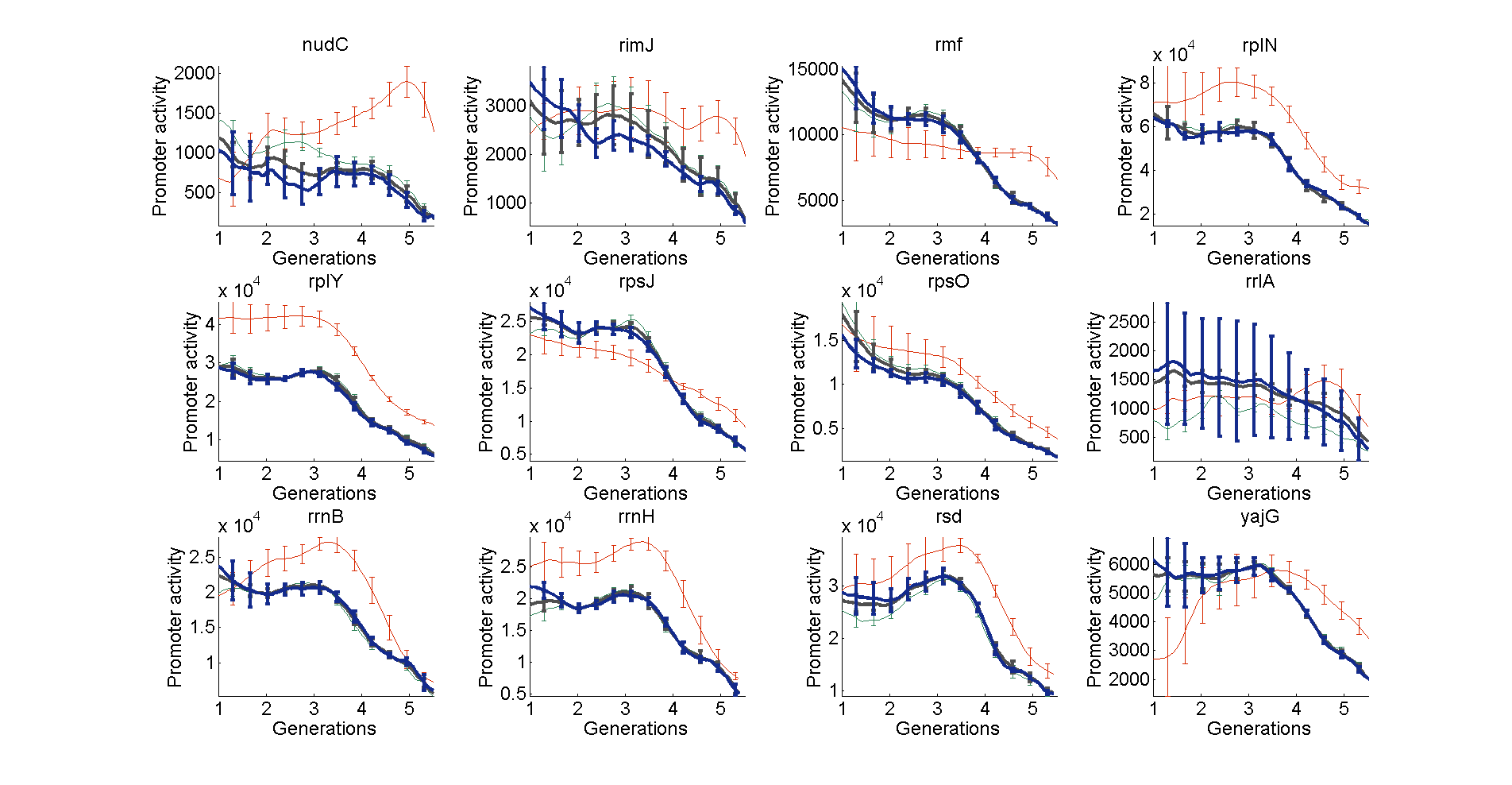


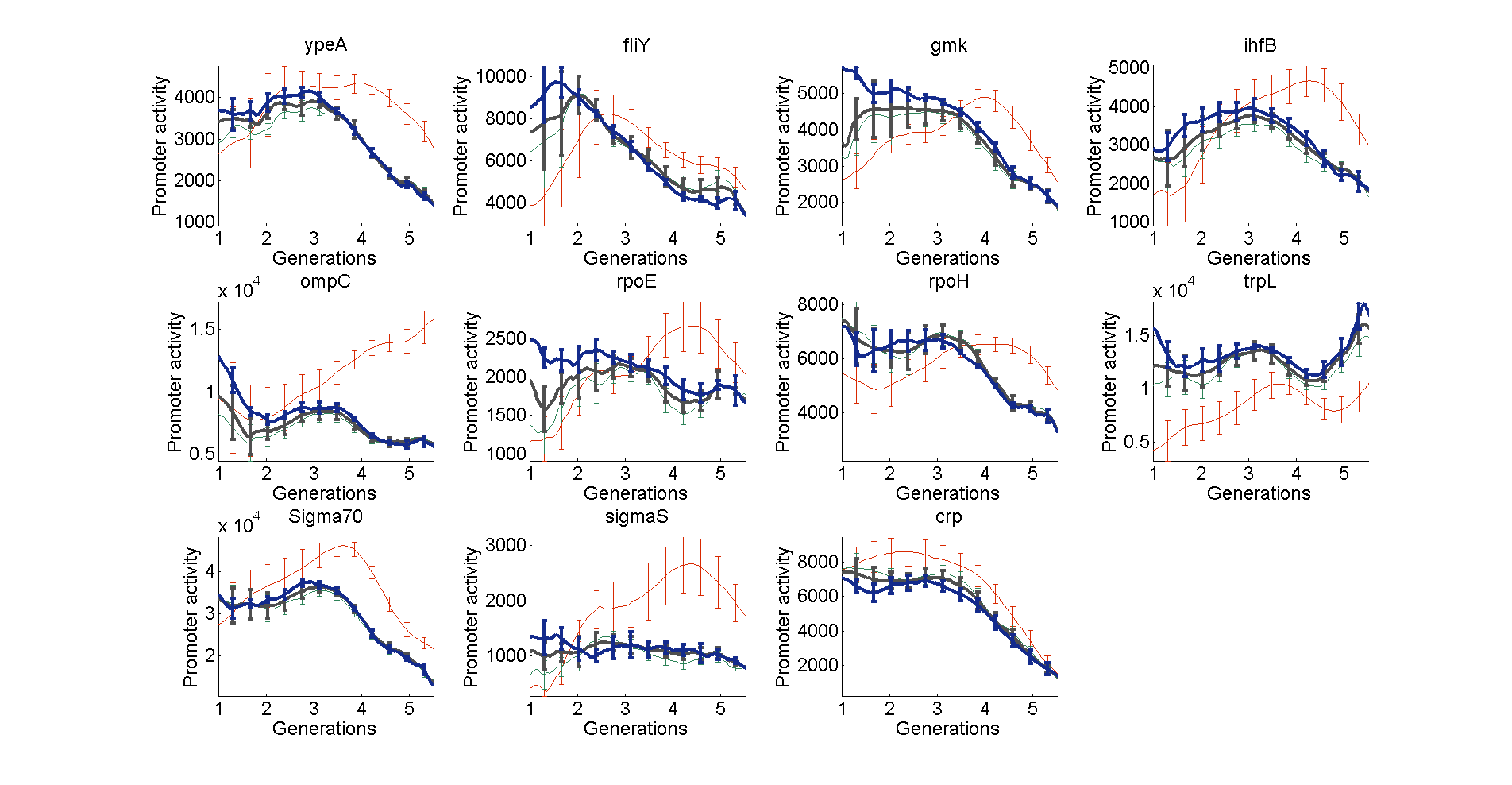


## Glucose, Casamino acids, Glucose + Casamino acids

Red – M9 only (not Standard medium) + Glucose 0.2%

Green – M9 only (not Standard medium) + Casamino acids 0.05%

Blue – M9 only (not Standard medium) + Glucose 0.2% + Casamino acids 0.05%

Black – best fit linear superposition


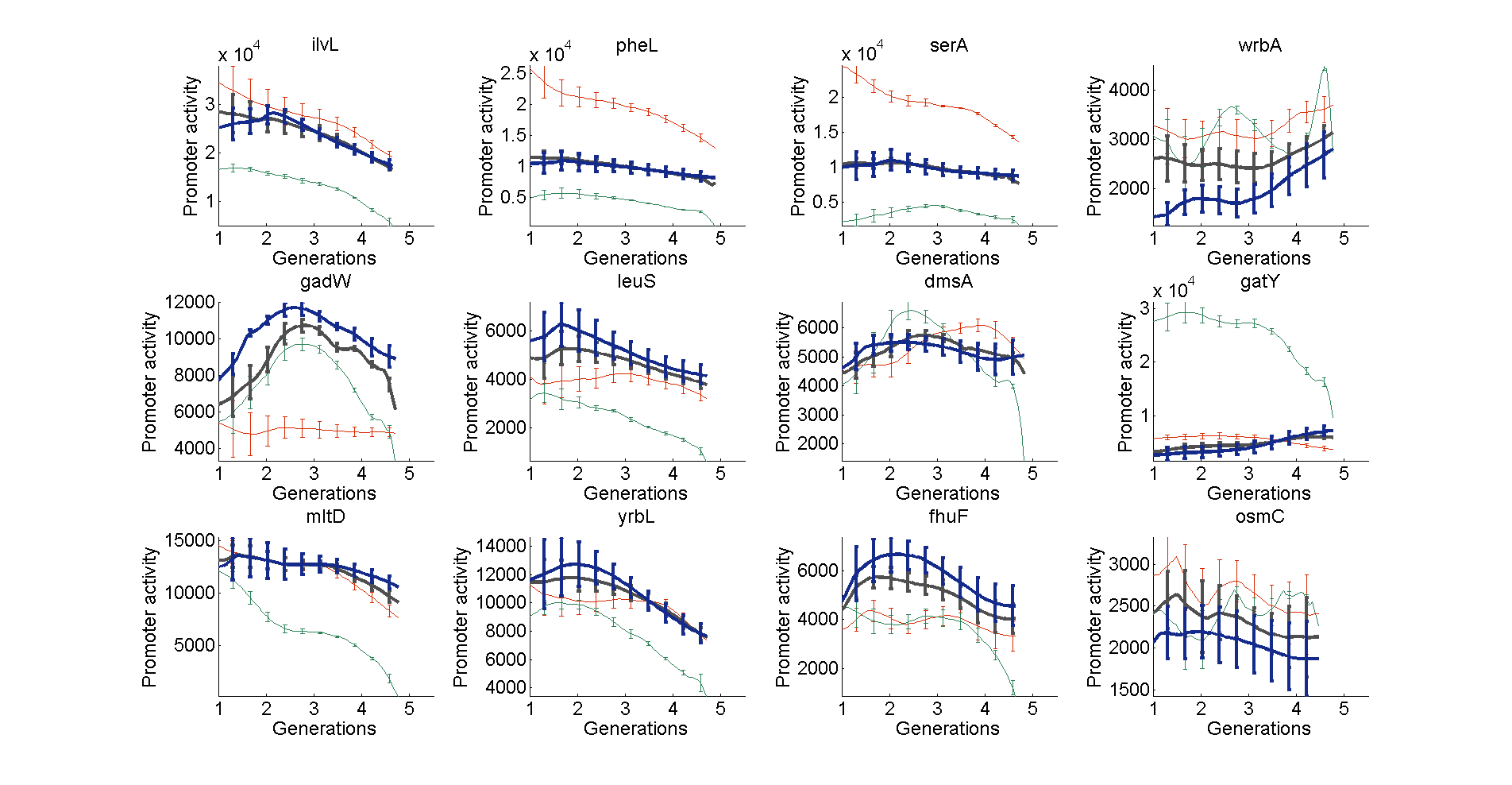


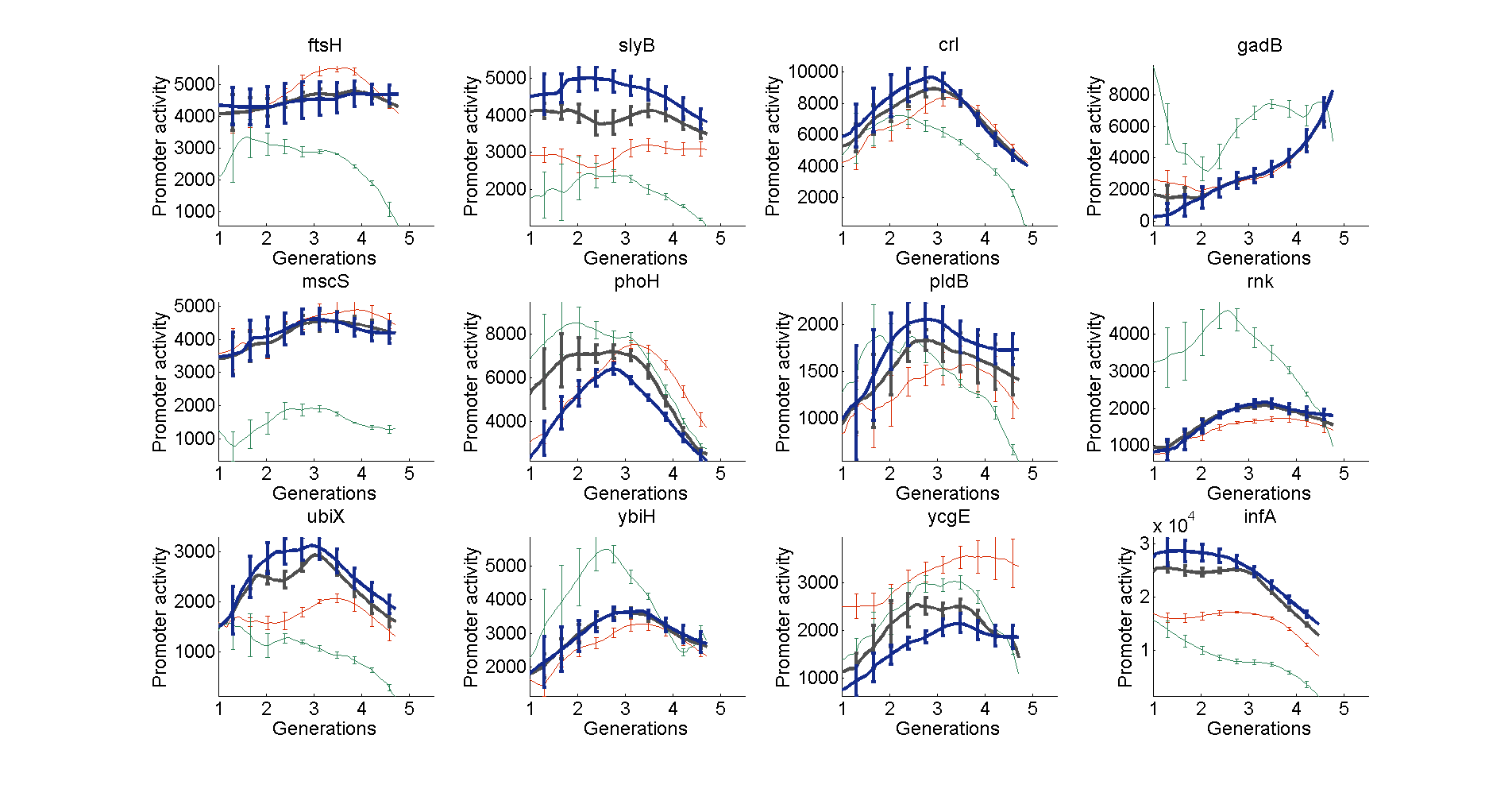


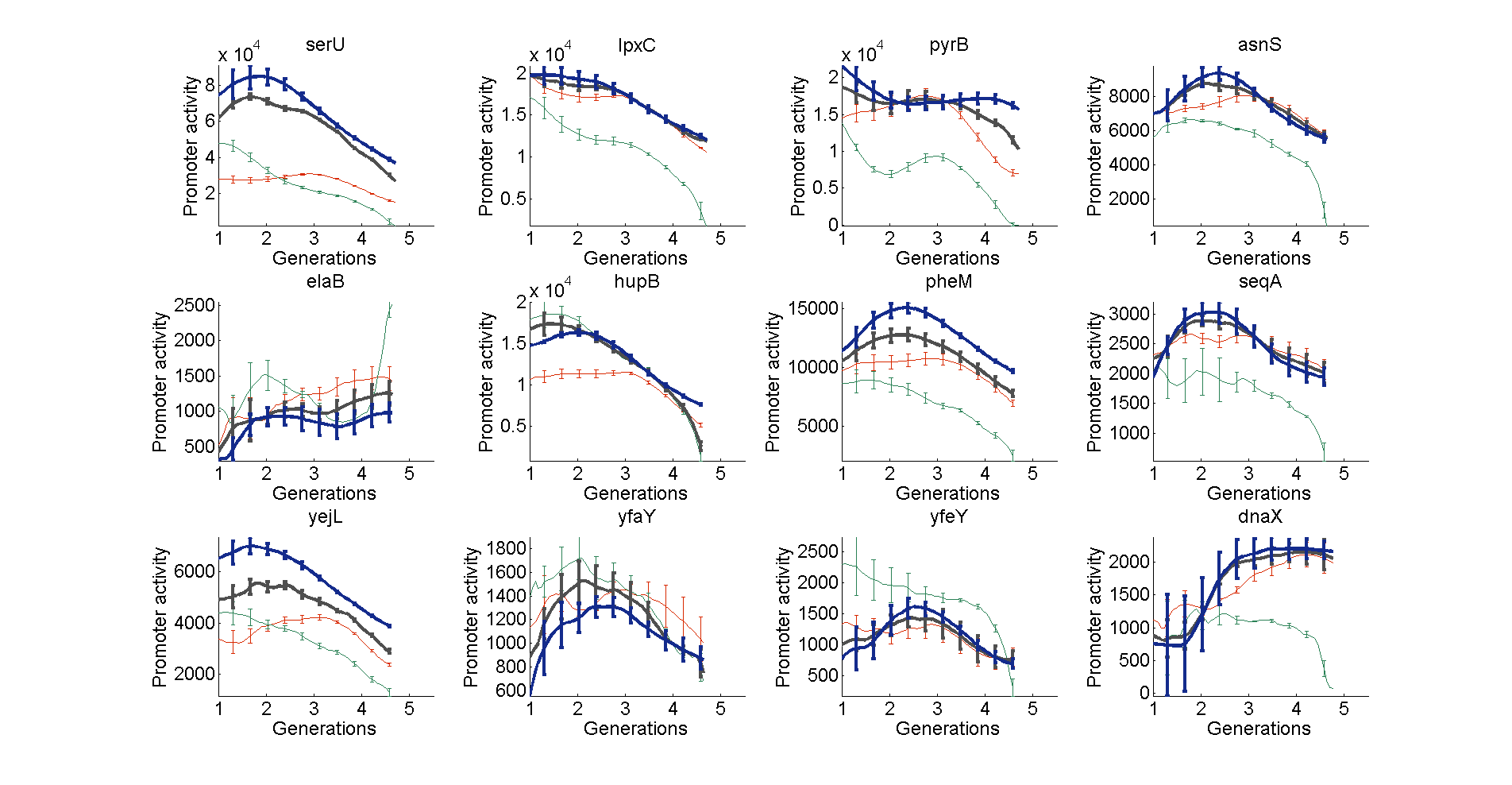


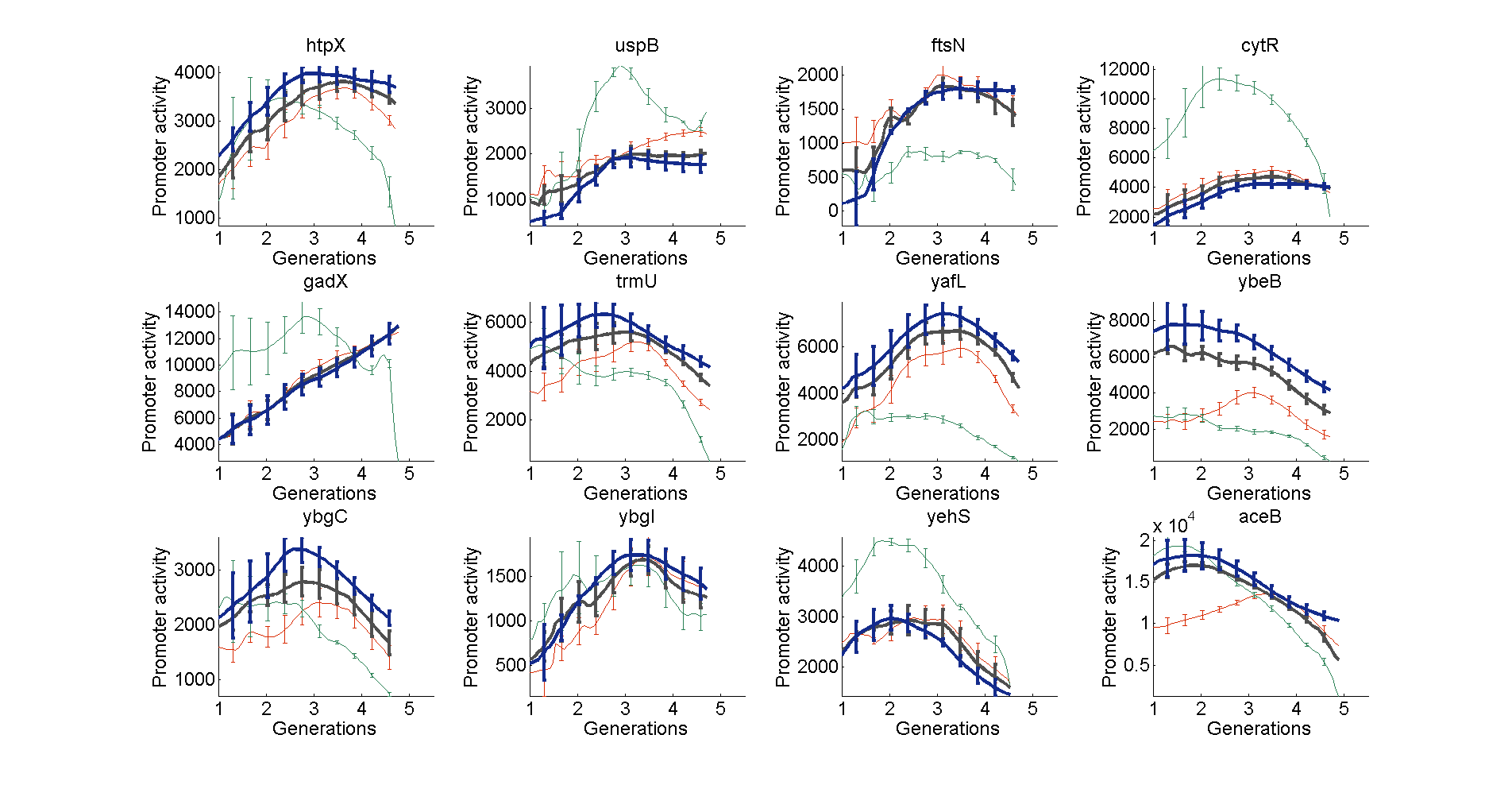


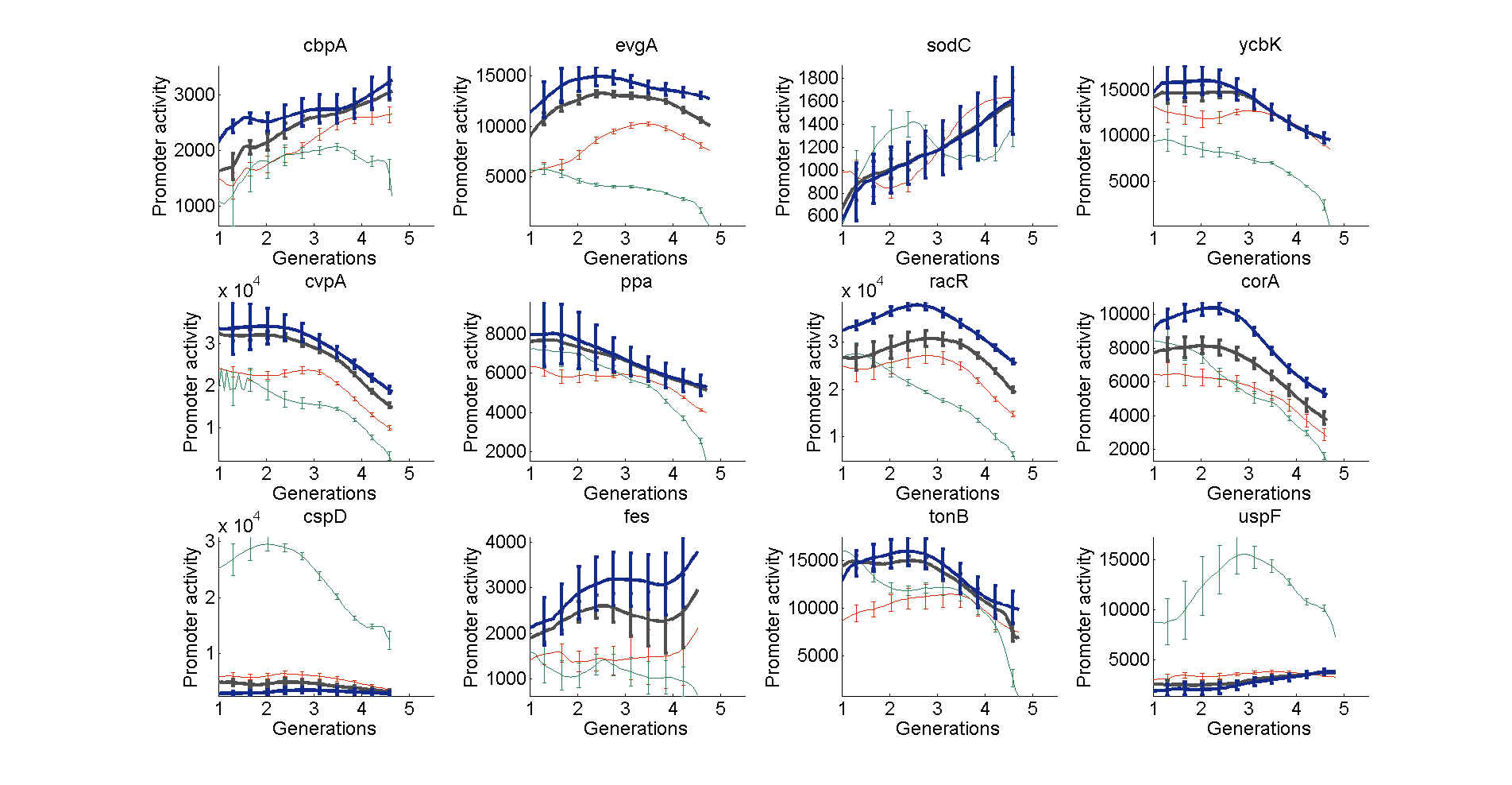


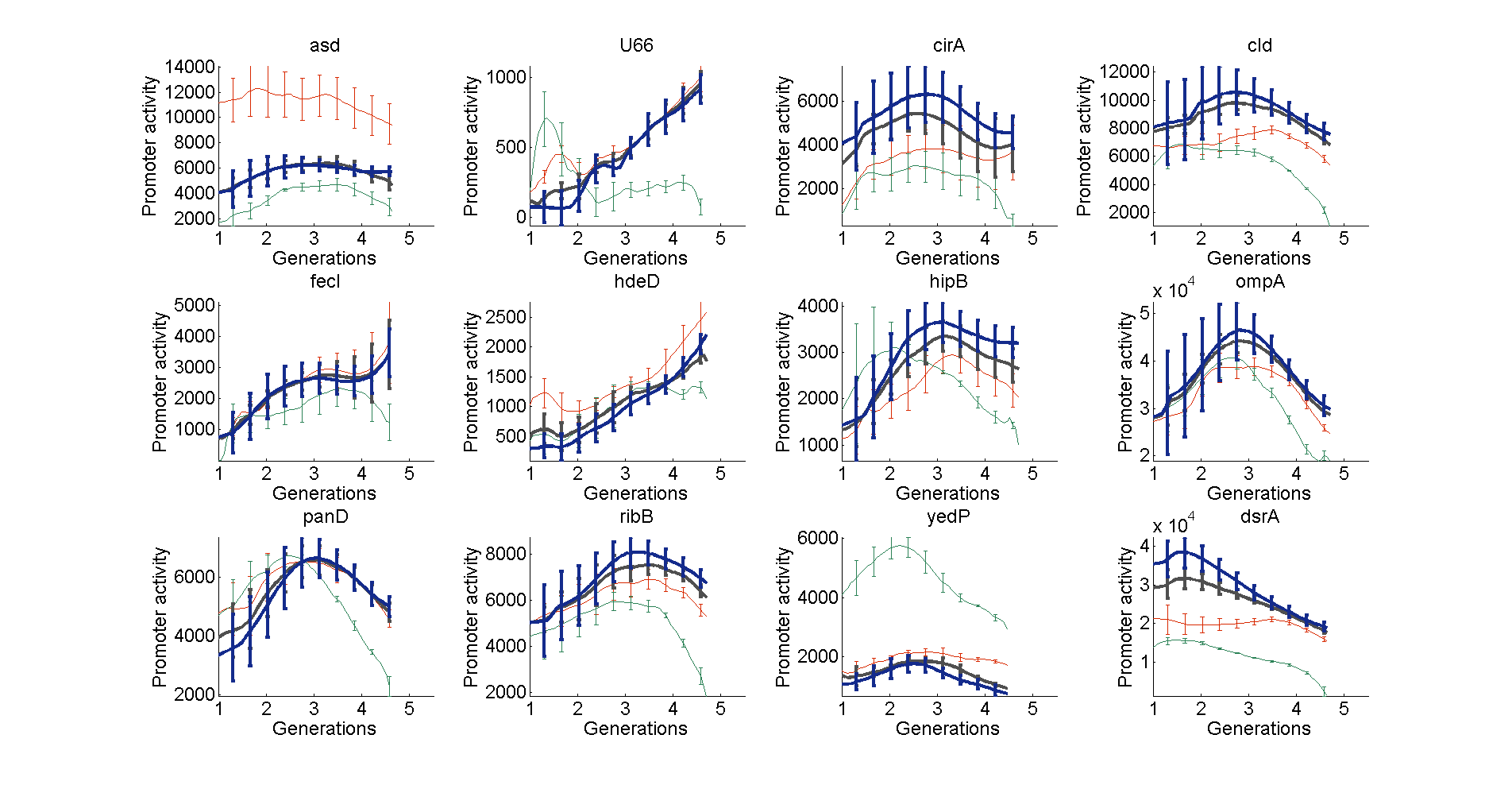


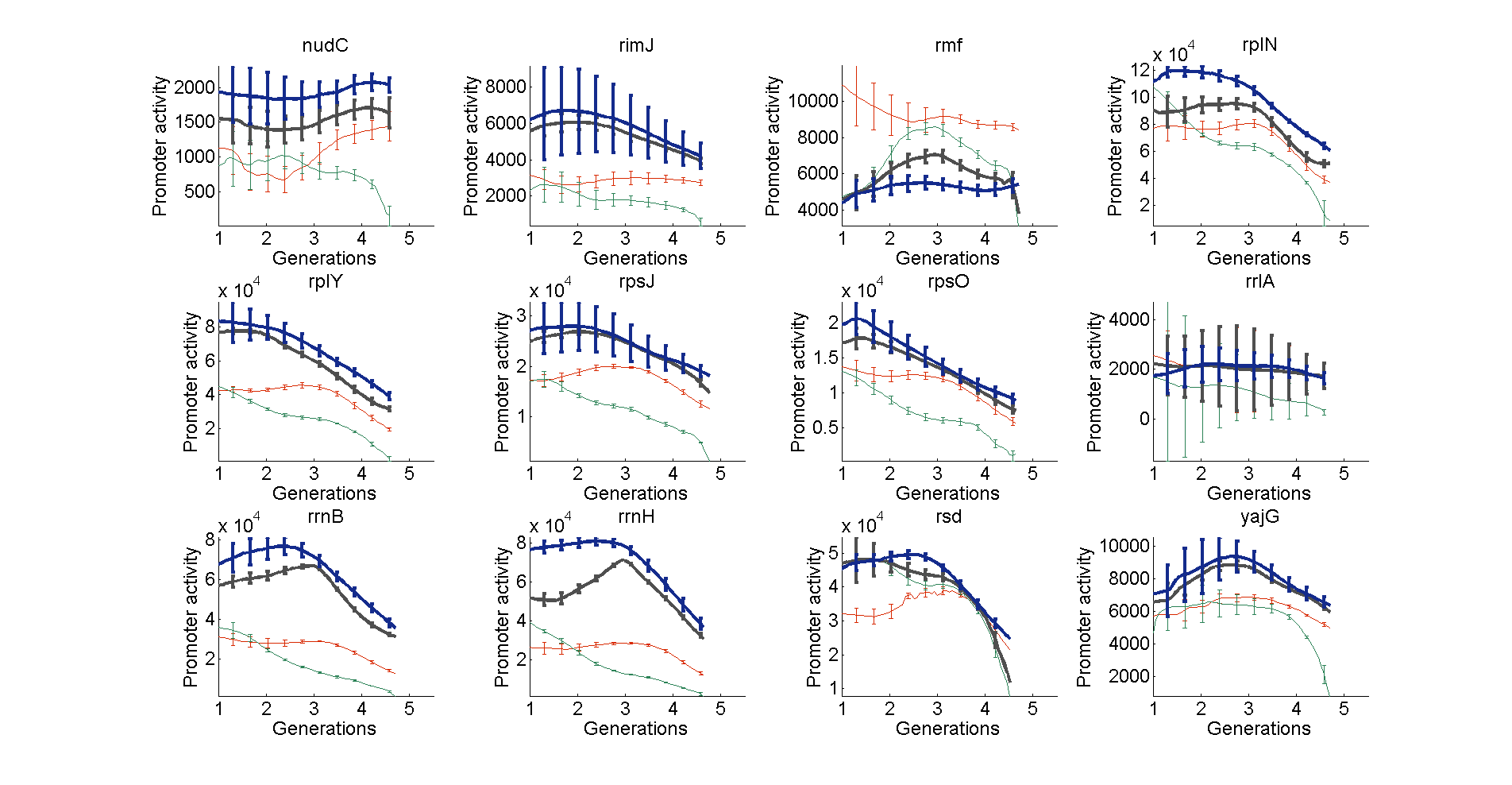


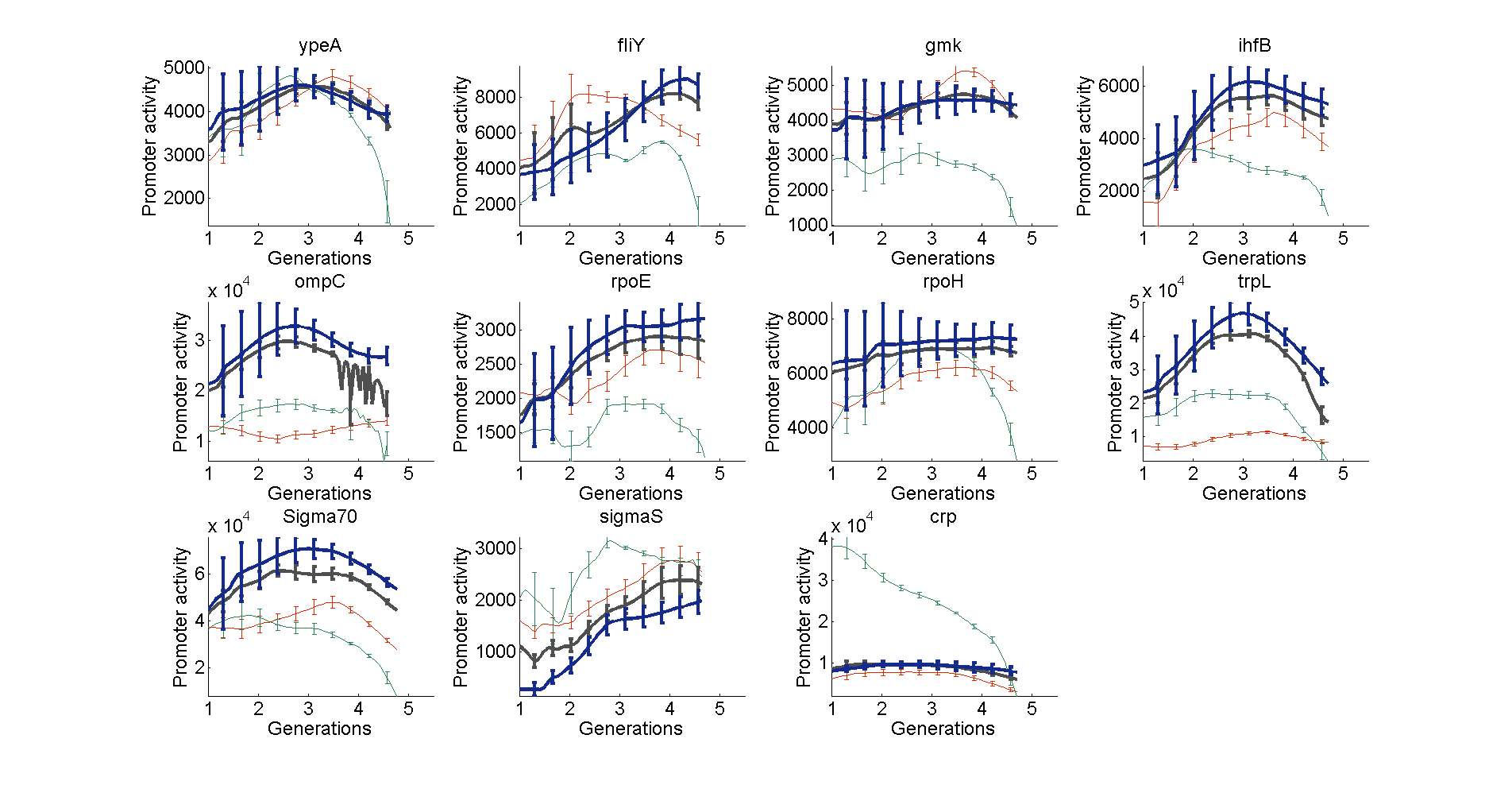


## NaCl, Ethanol, Casamino acids, NaCl + Ethanol + Casamino acids

Red – Standard medium + NaCl 300mM

Green – Standard medium + Ethanol 3%

Pink – Standard medium + Casamino acids 0.05%

Blue – Standard medium + NaCl 300mM + Ethanol 3% + Casamino acids 0.05%

Black – Best fit linear superposition

Orange – Predicion of NaCl 300mM + Ethanol 3% + Casamino acids 0.05%


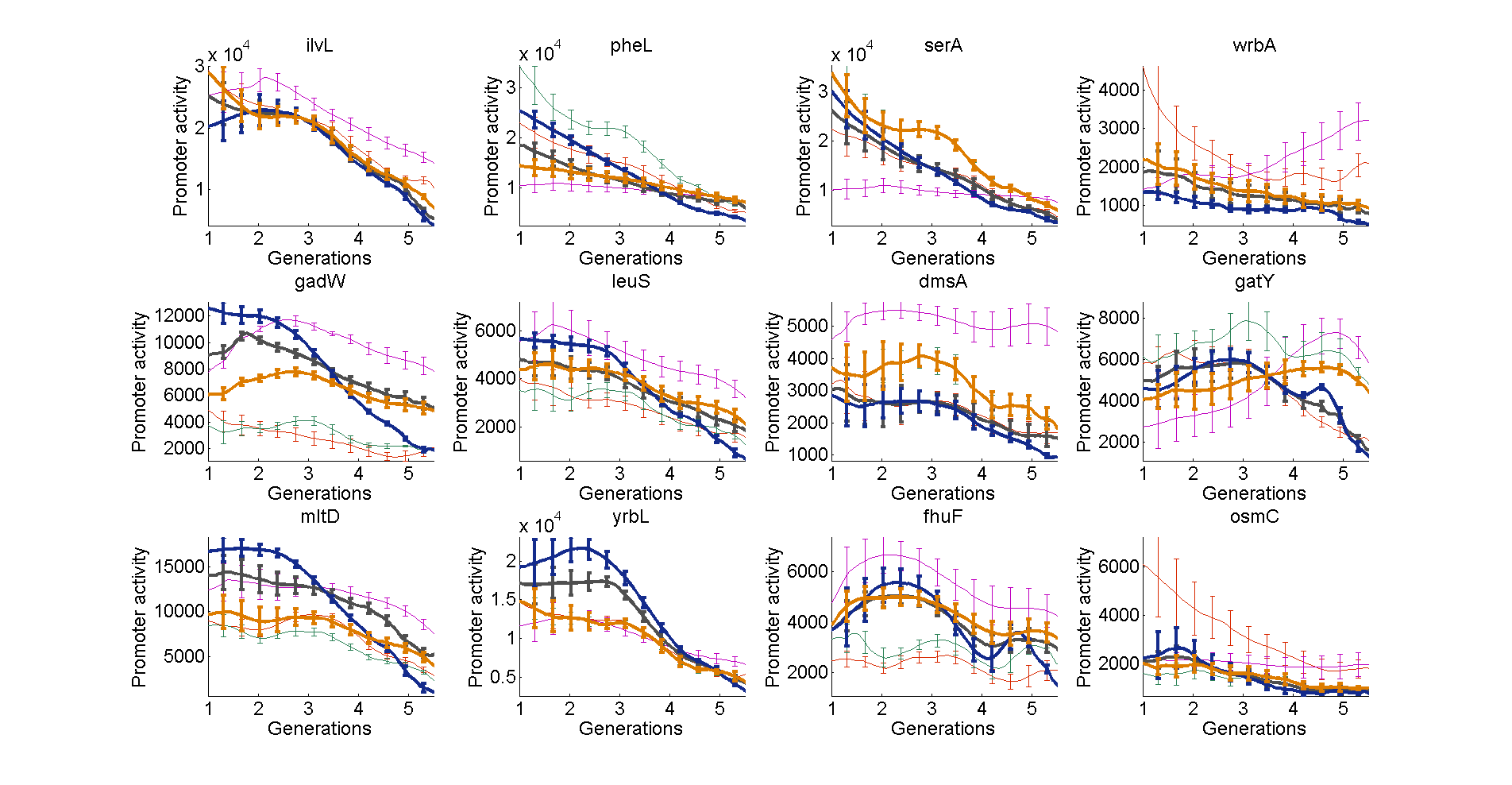


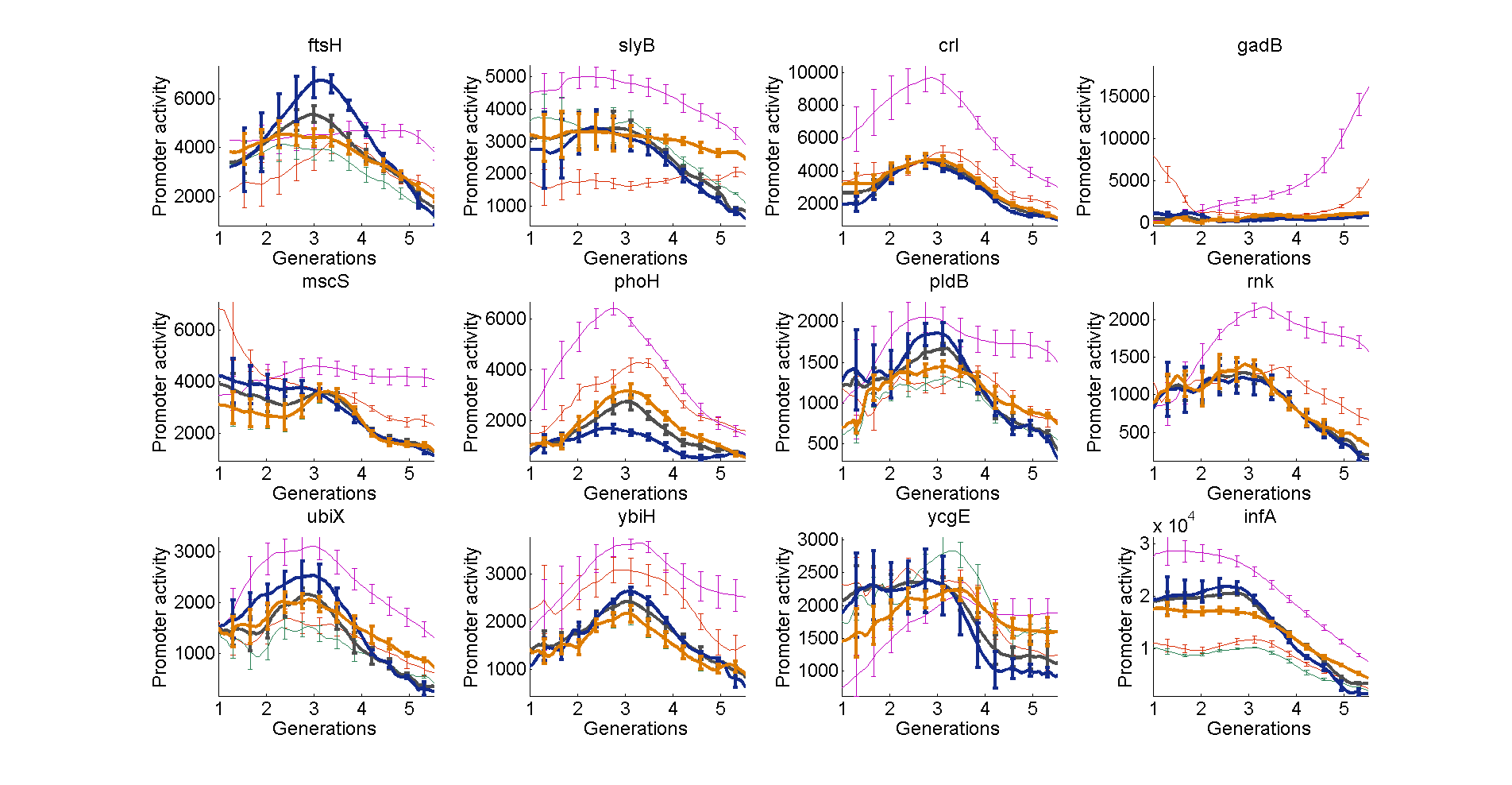


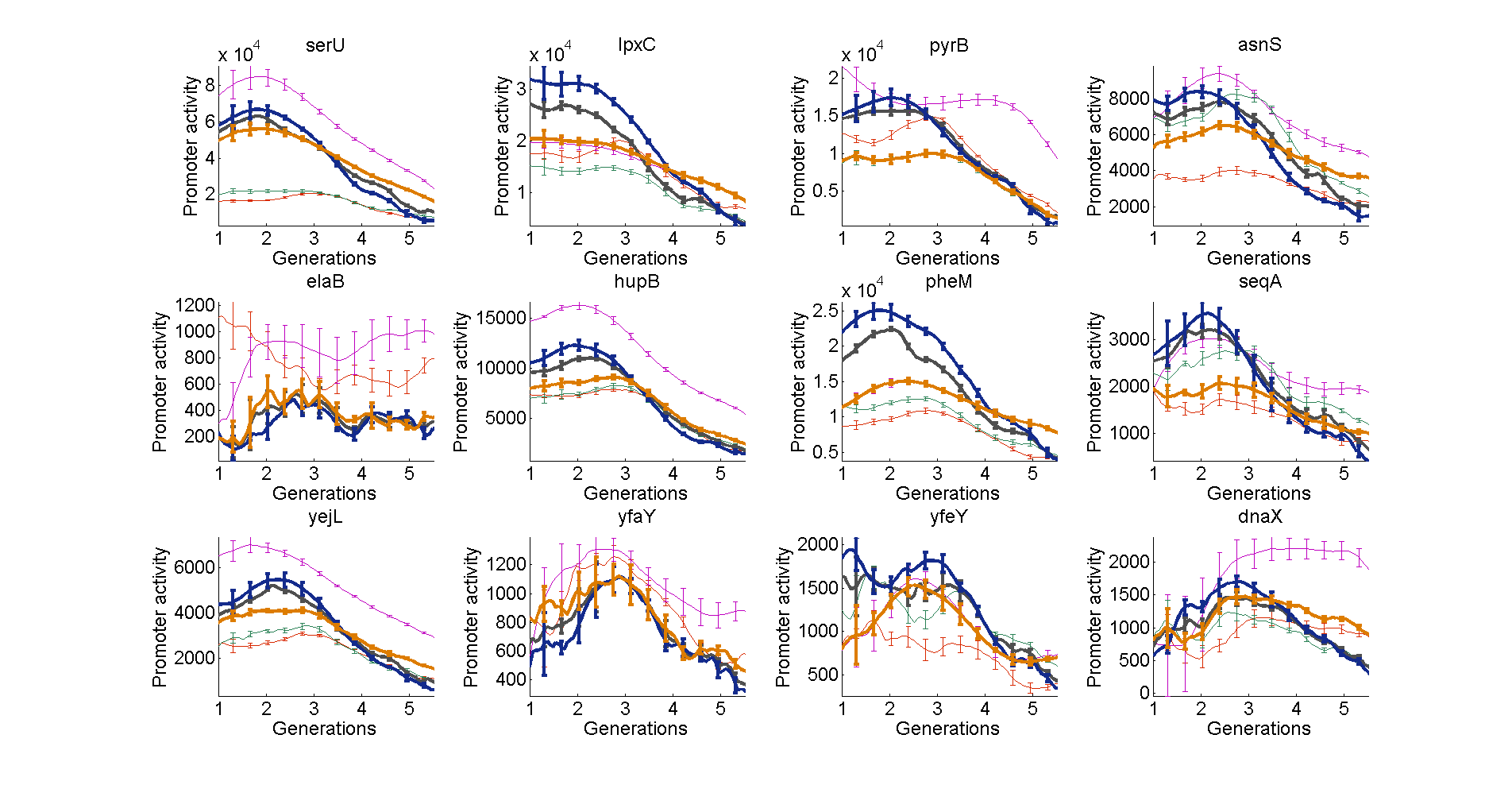


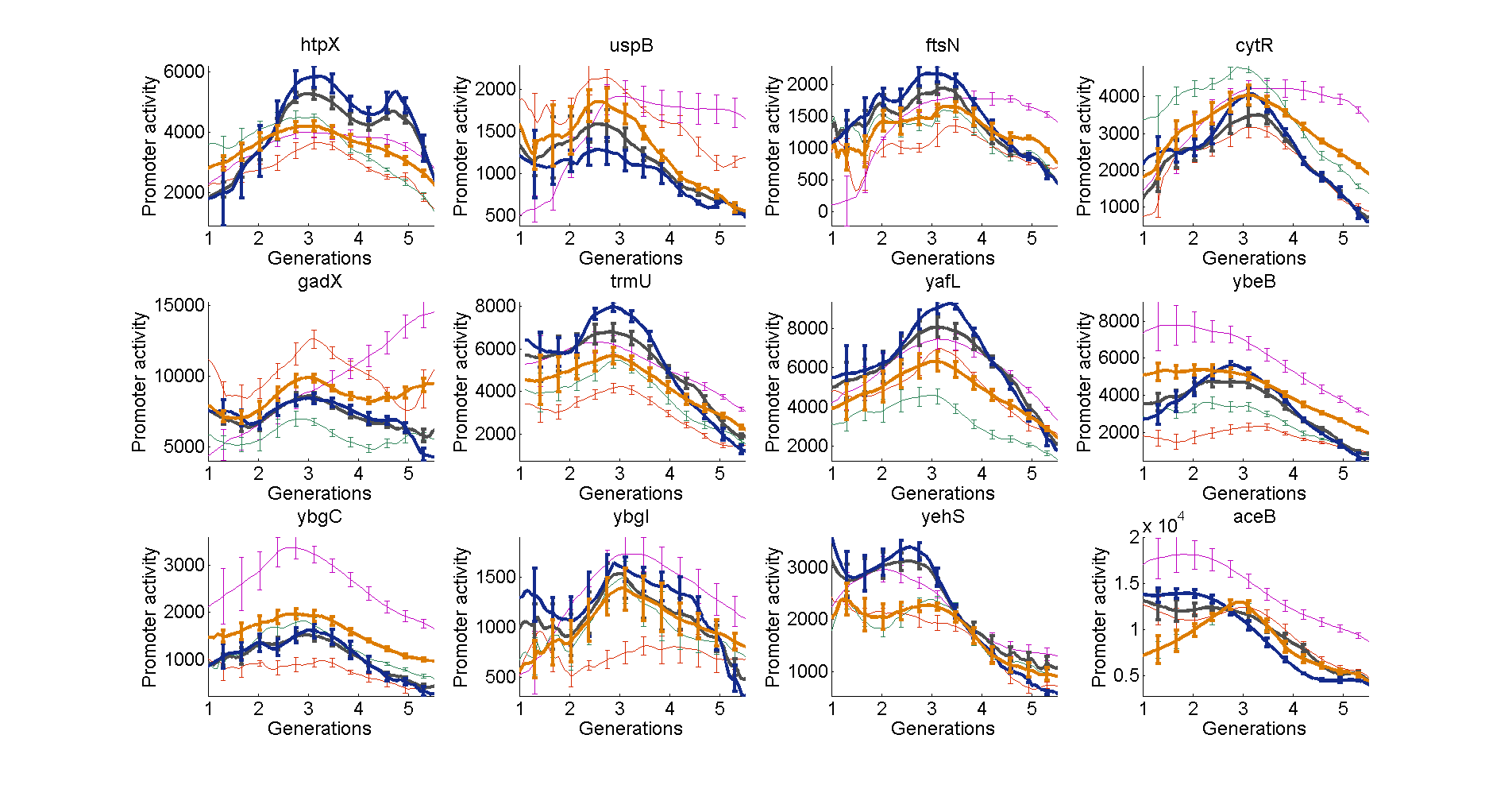


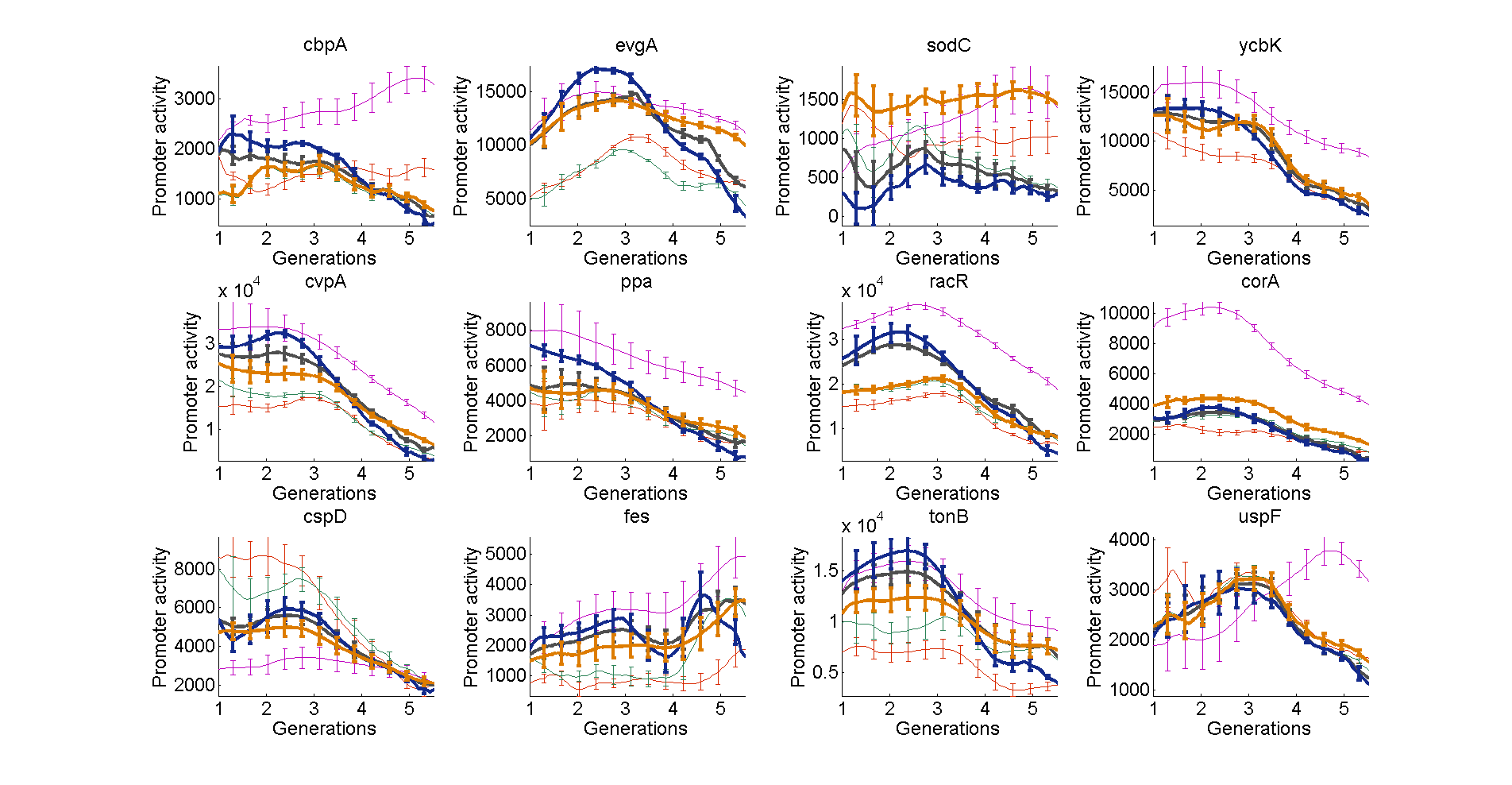


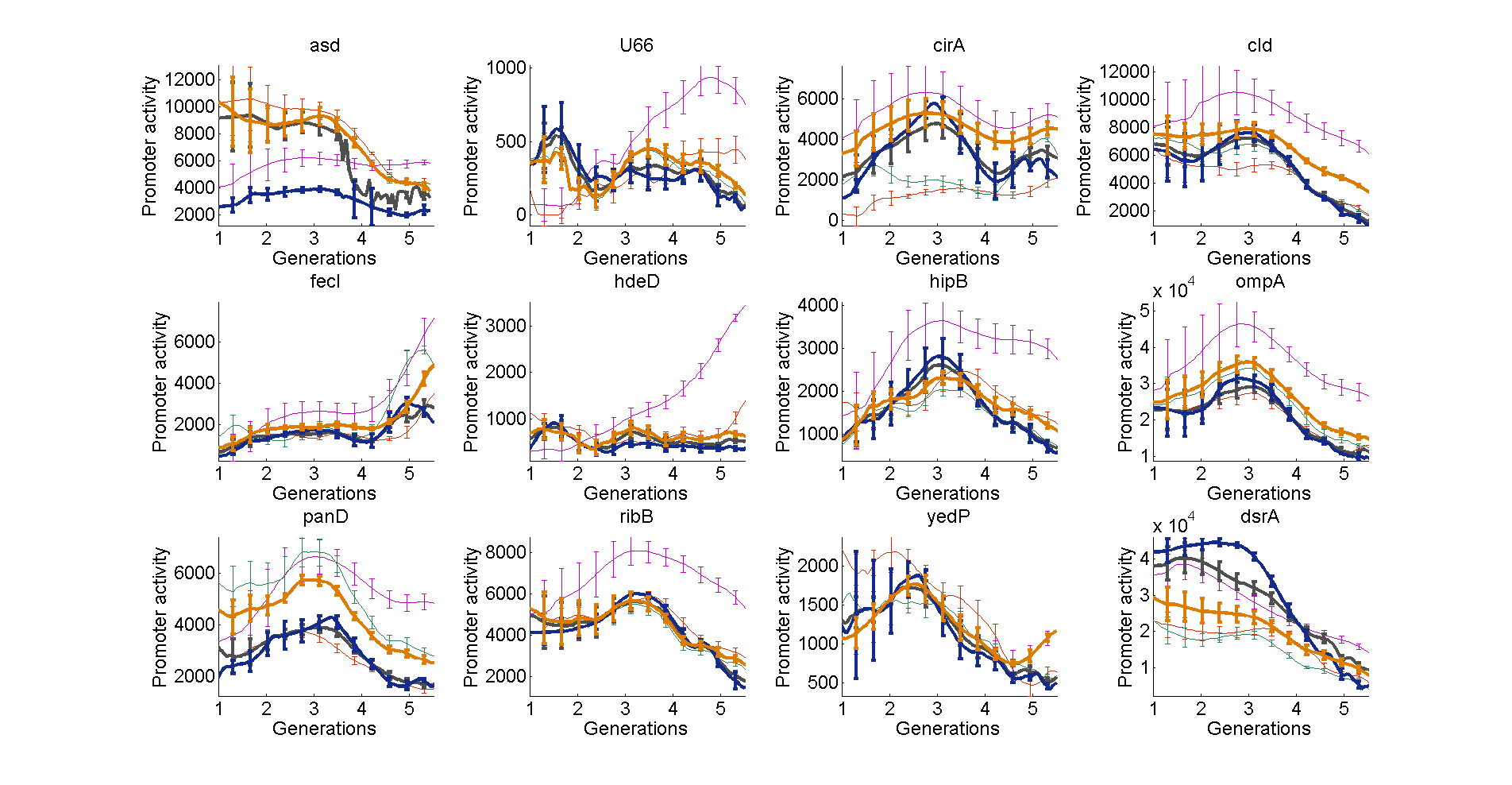


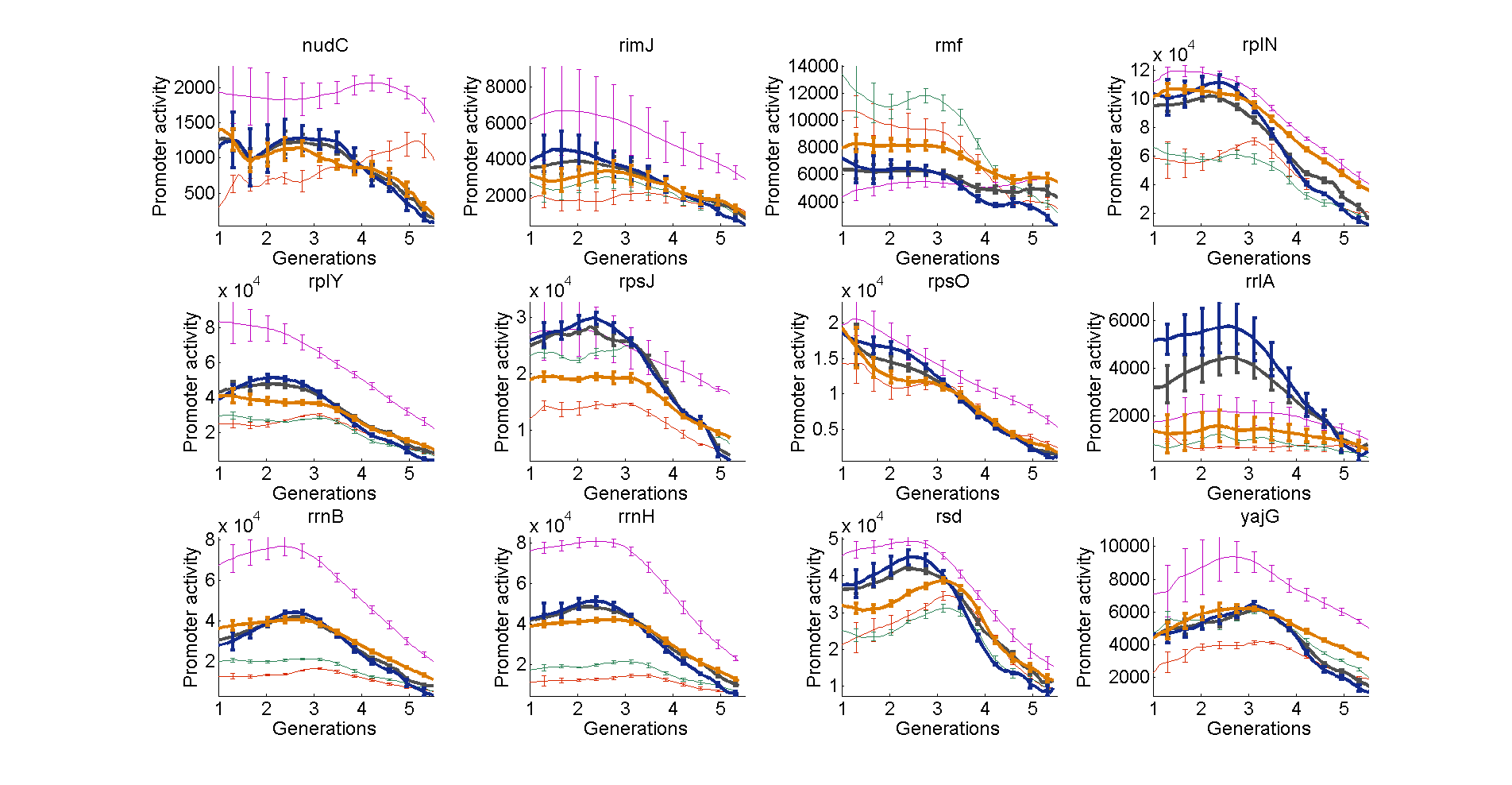


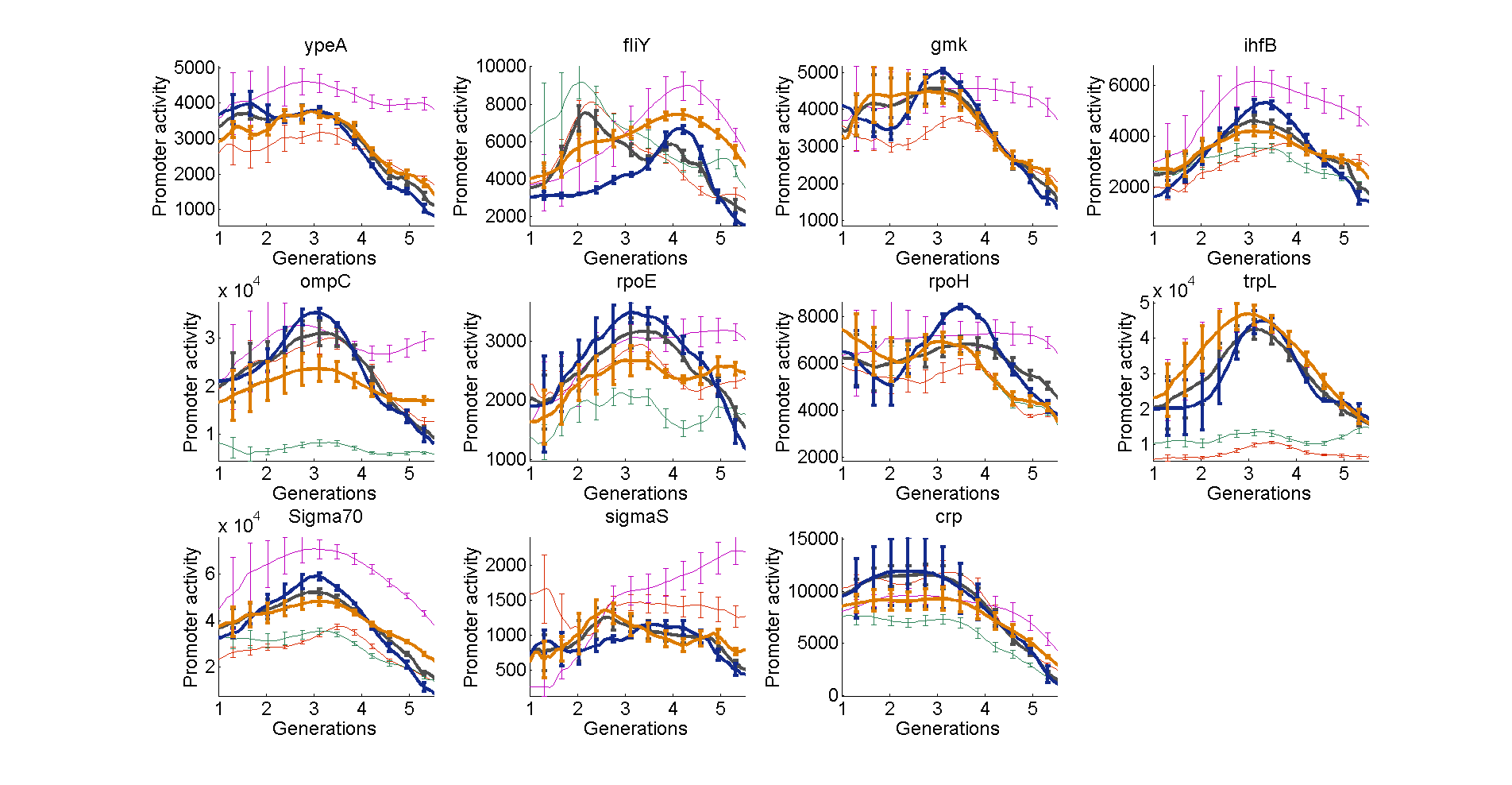


## NaCl, H_2_O_2_, Casamino acids, NaCl + H_2_O_2_ + Casamino acids

Red – Standard medium + NaCl 300mM

Green – Standard medium + H_2_O_2_ 10µM

Pink – Standard medium + Casamino acids 0.05%

Blue – Standard medium + NaCl 300mM + H_2_O_2_ 10µM + Casamino acids 0.05%

Black – Best fit linear superposition

Orange – Predicion of NaCl 300mM + H_2_O_2_ 10µM + Casamino acids 0.05%


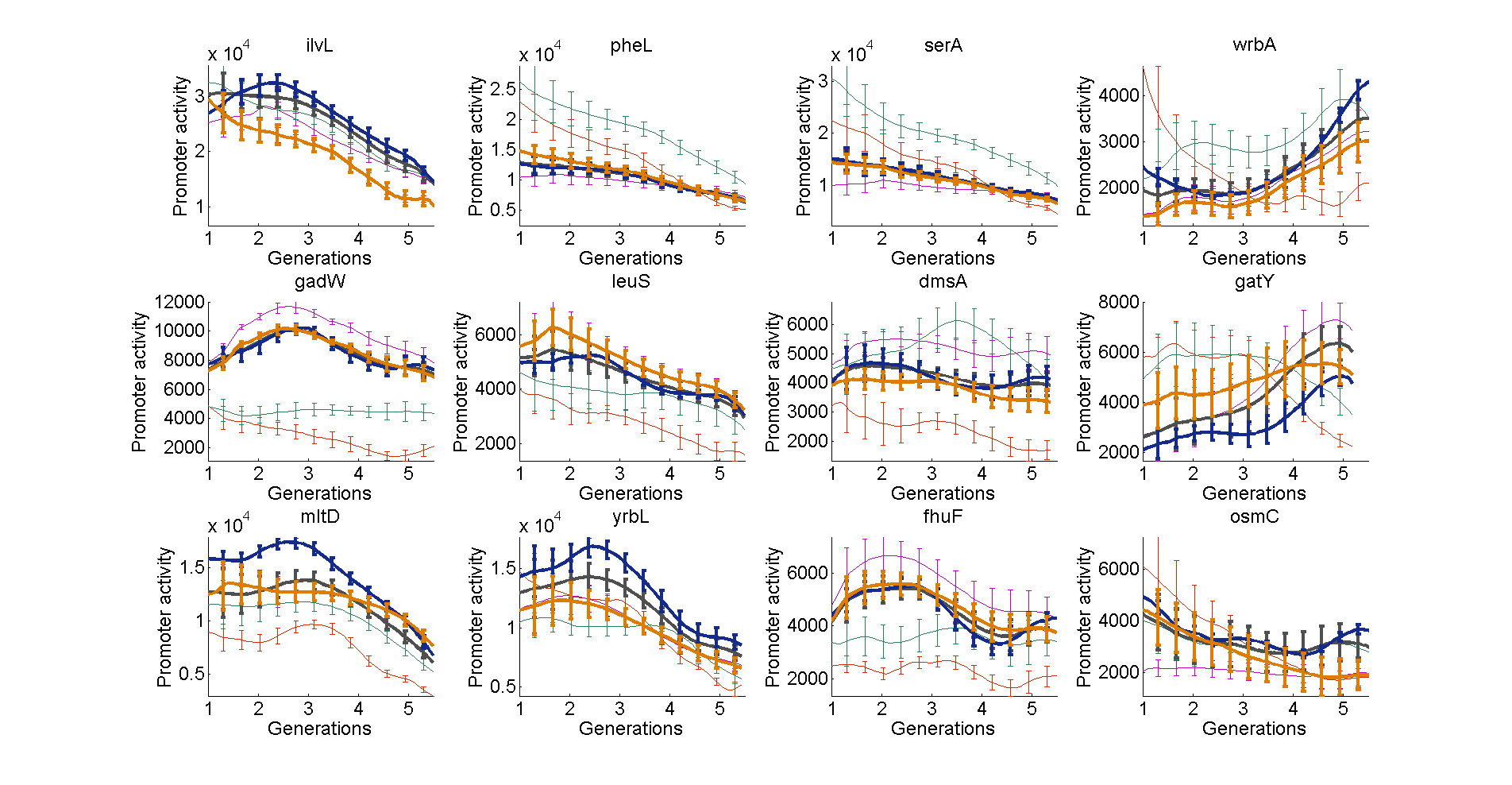


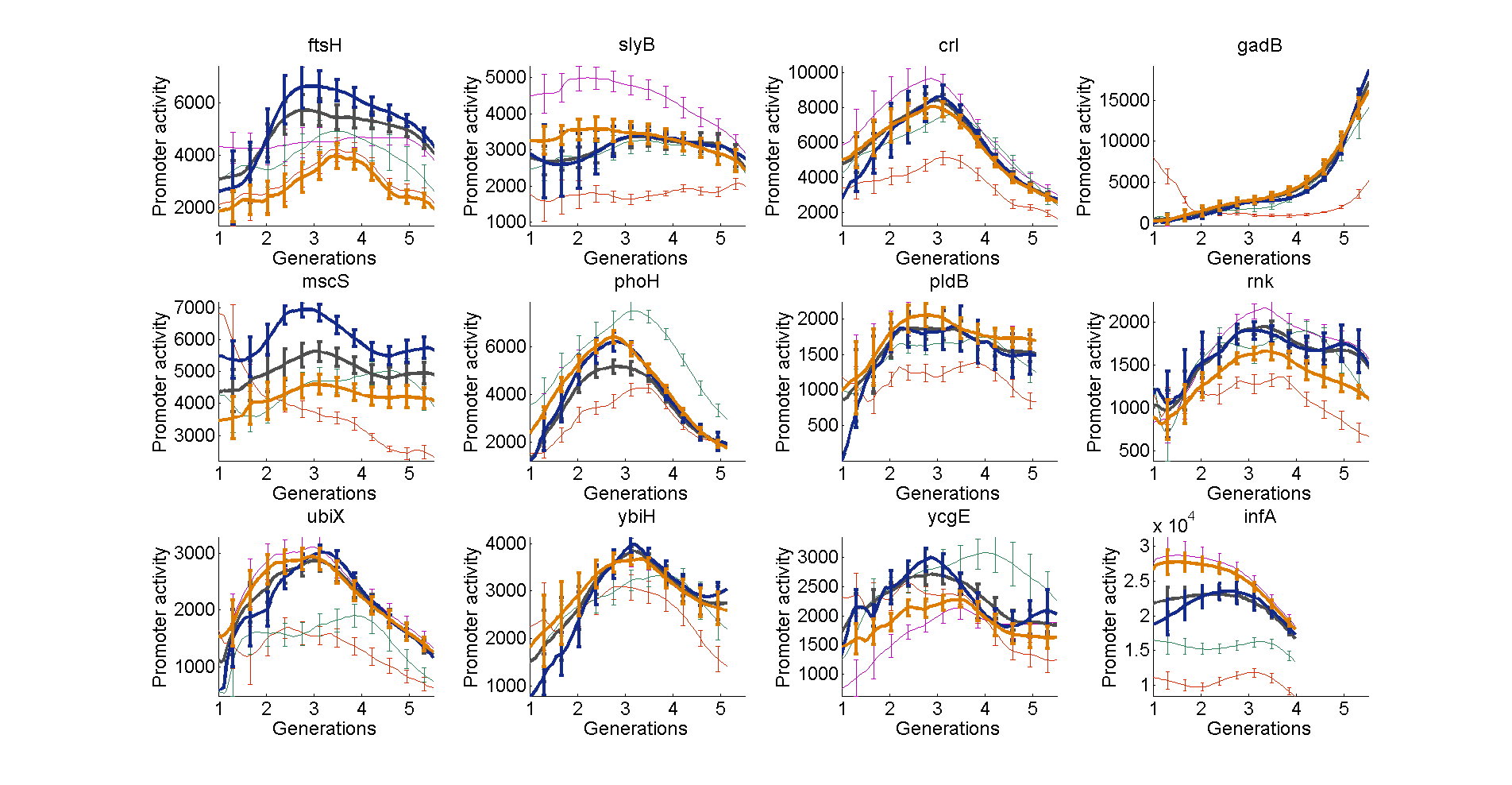


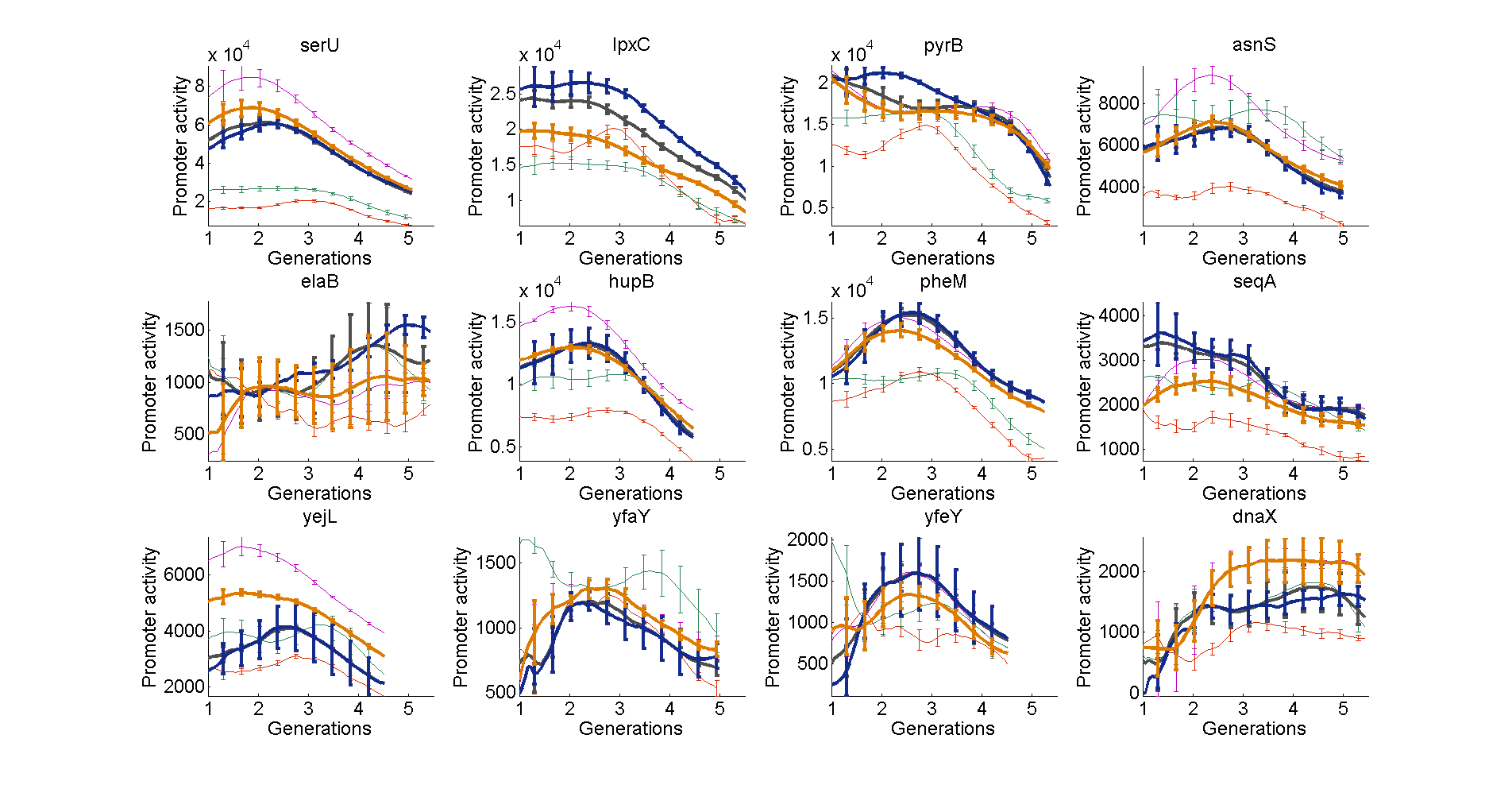


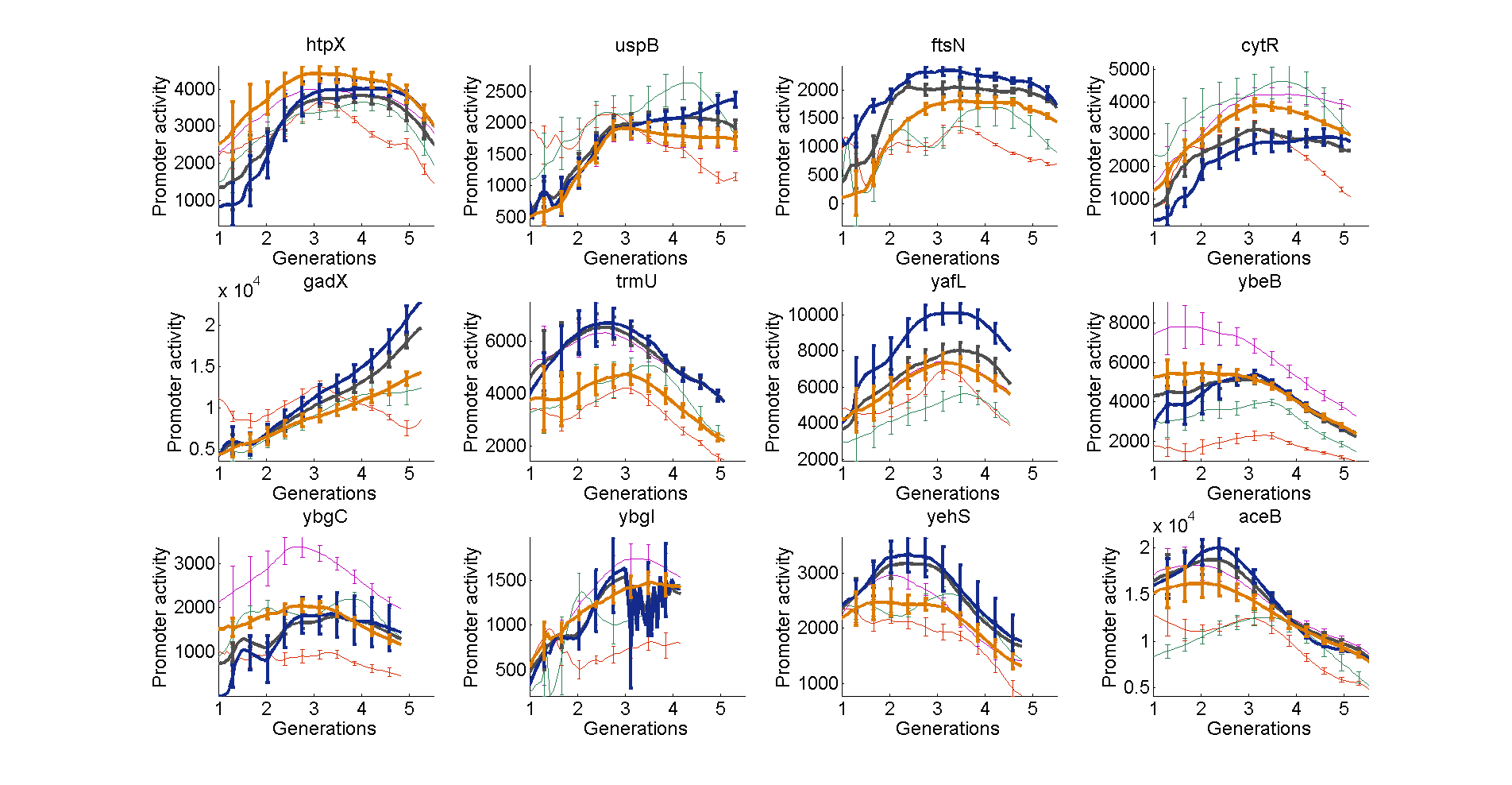


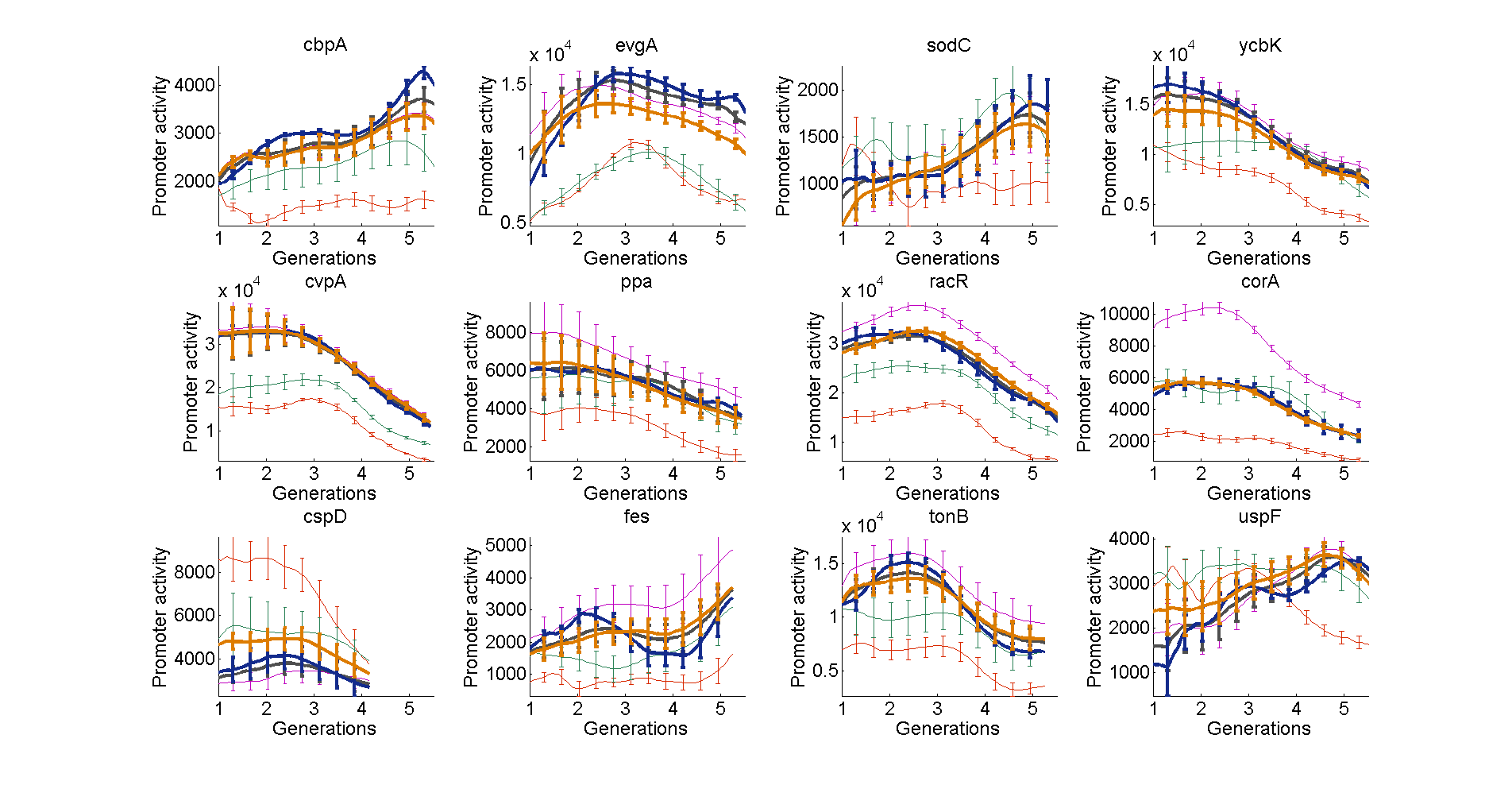


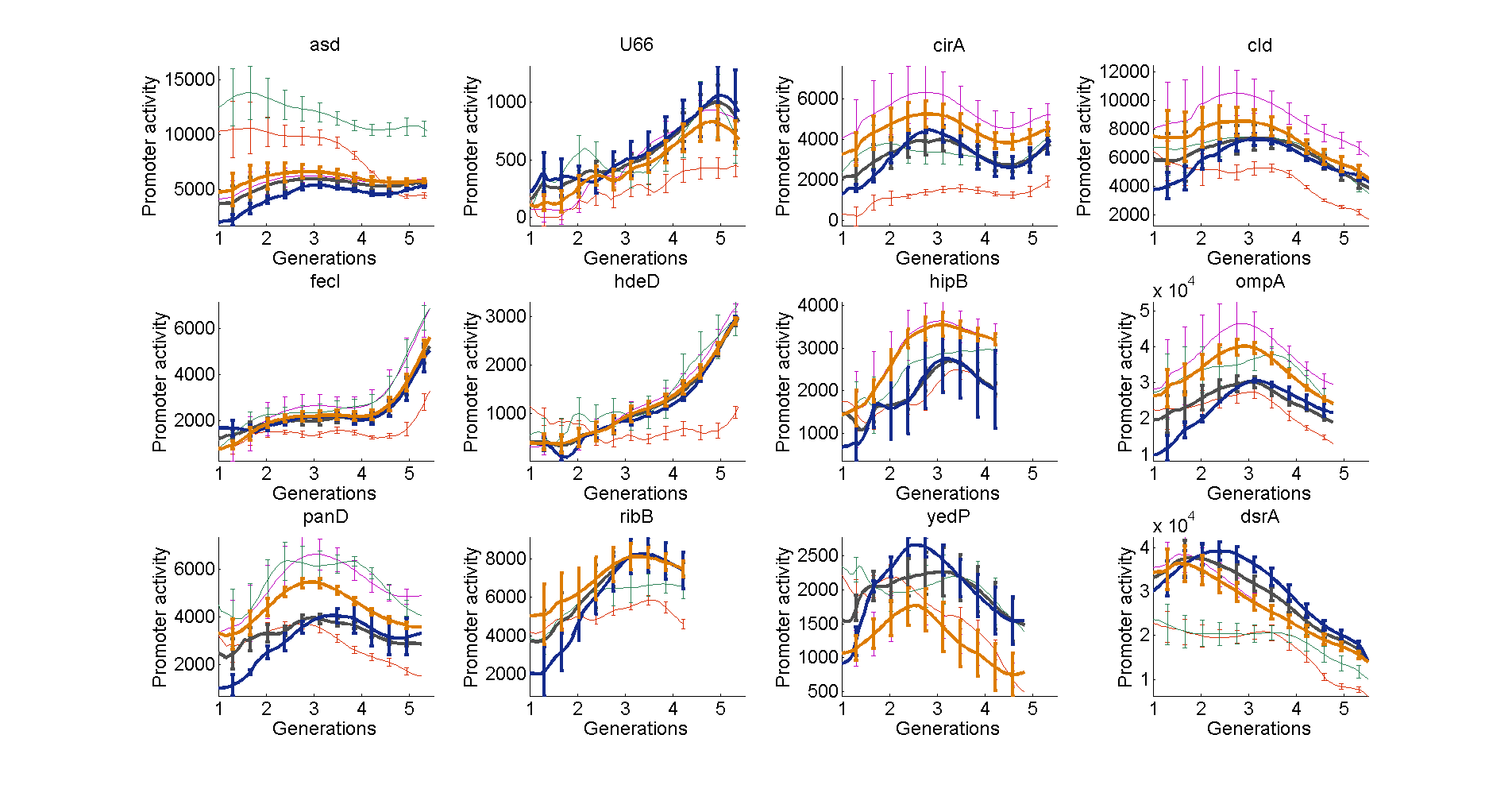


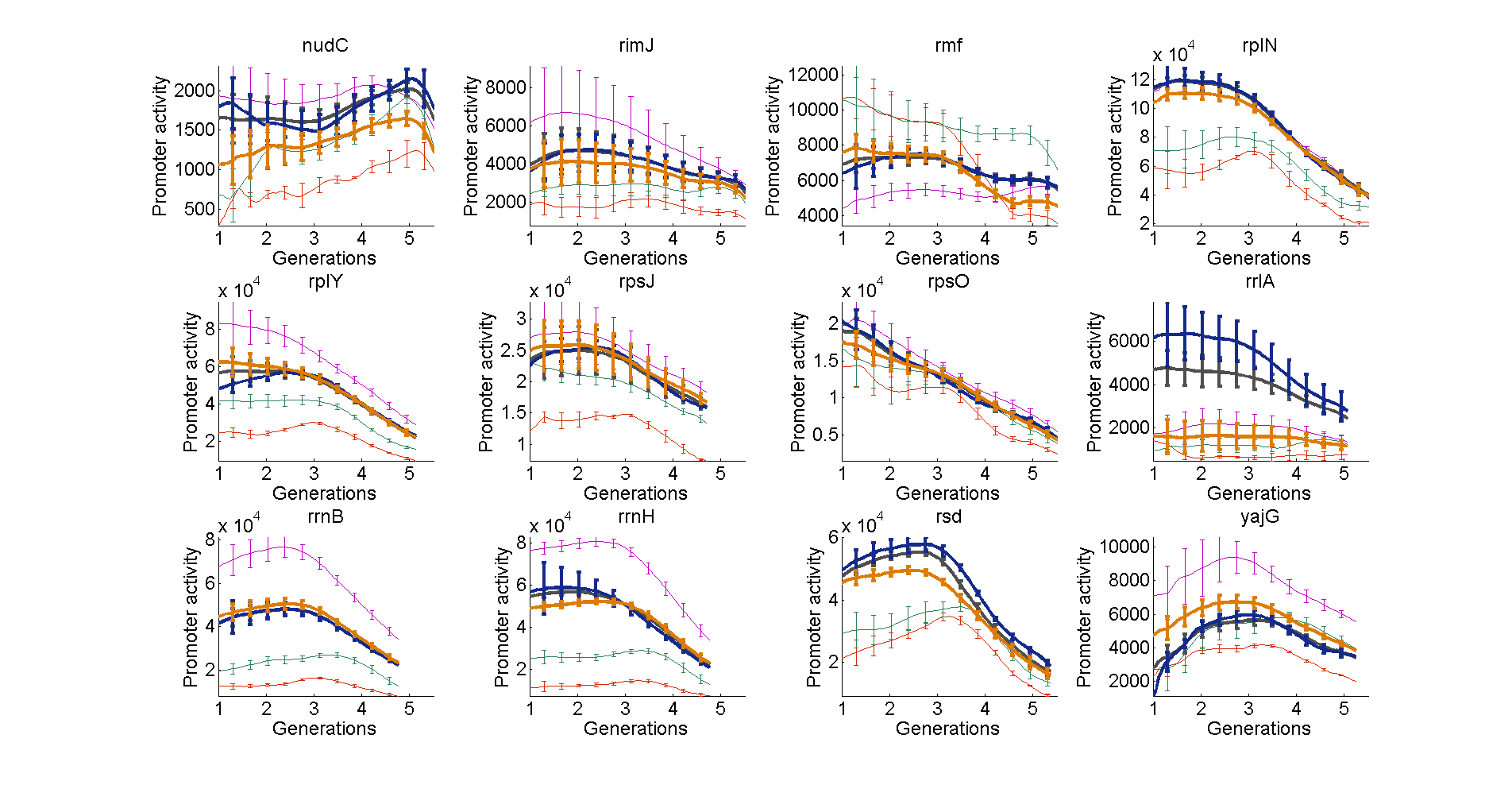


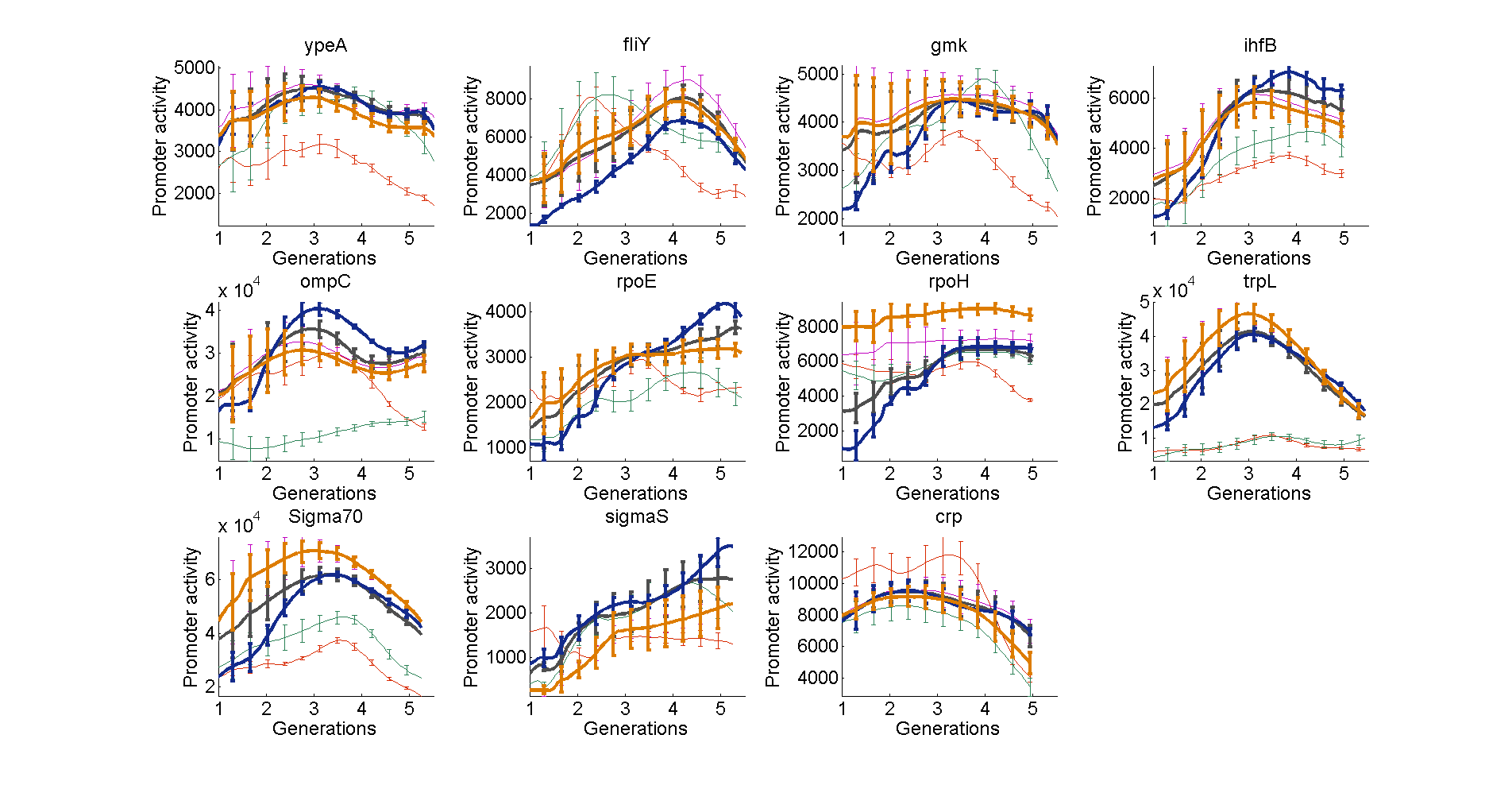


## NaCl, Ethanol, H_2_O_2_, NaCl + Ethanol + H_2_O_2_

Red – Standard medium + NaCl 300mM

Green – Standard medium + Ethanol 3%

Pink – Standard medium + H_2_O_2_ 10µM

Blue – Standard medium + NaCl 300mM + Ethanol 3% + H_2_O_2_ 10µM

Black – Best fit linear superposition

Orange – Predicion of NaCl 300mM + Ethanol 3% + H_2_O_2_ 10µM


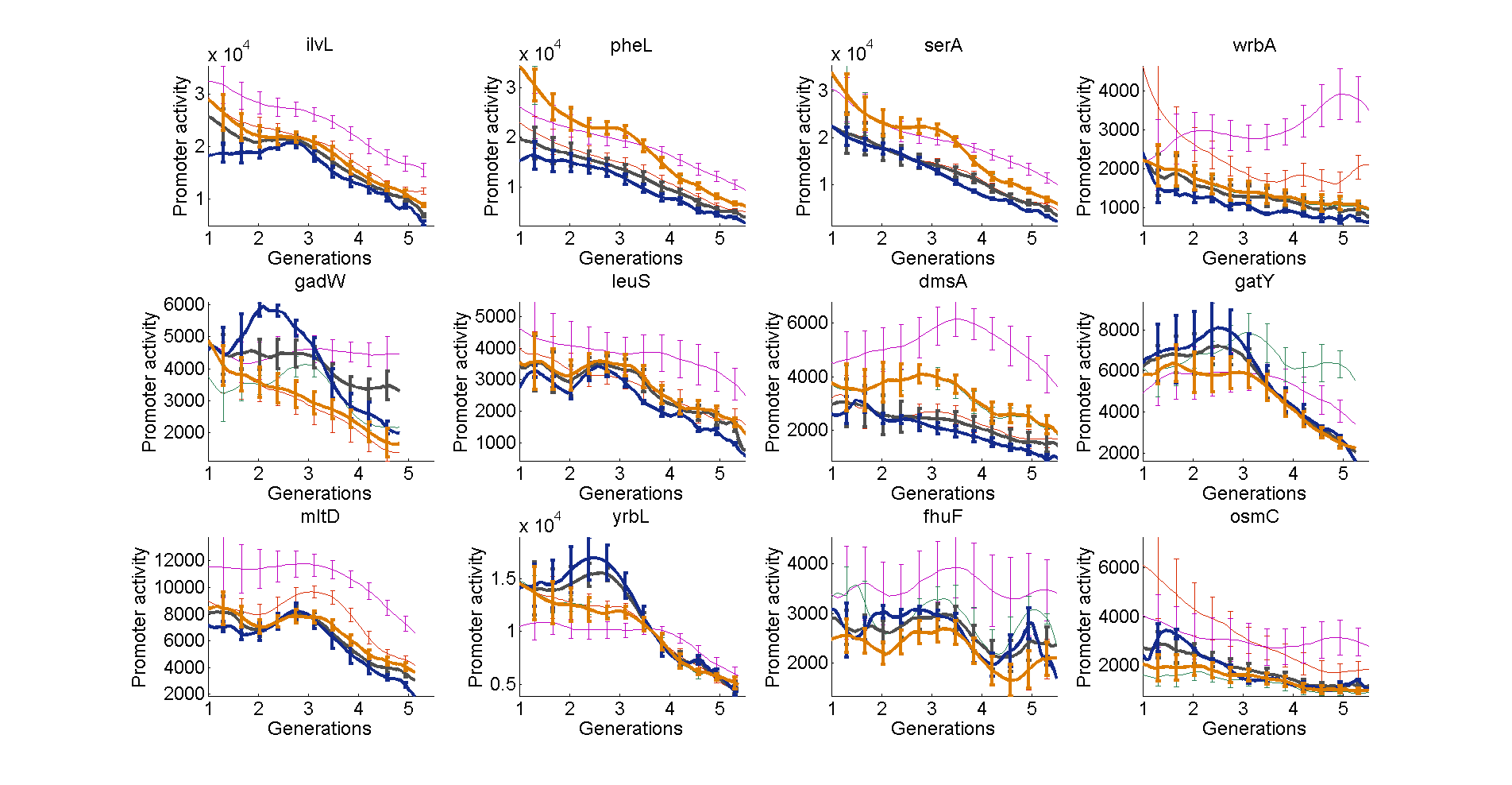


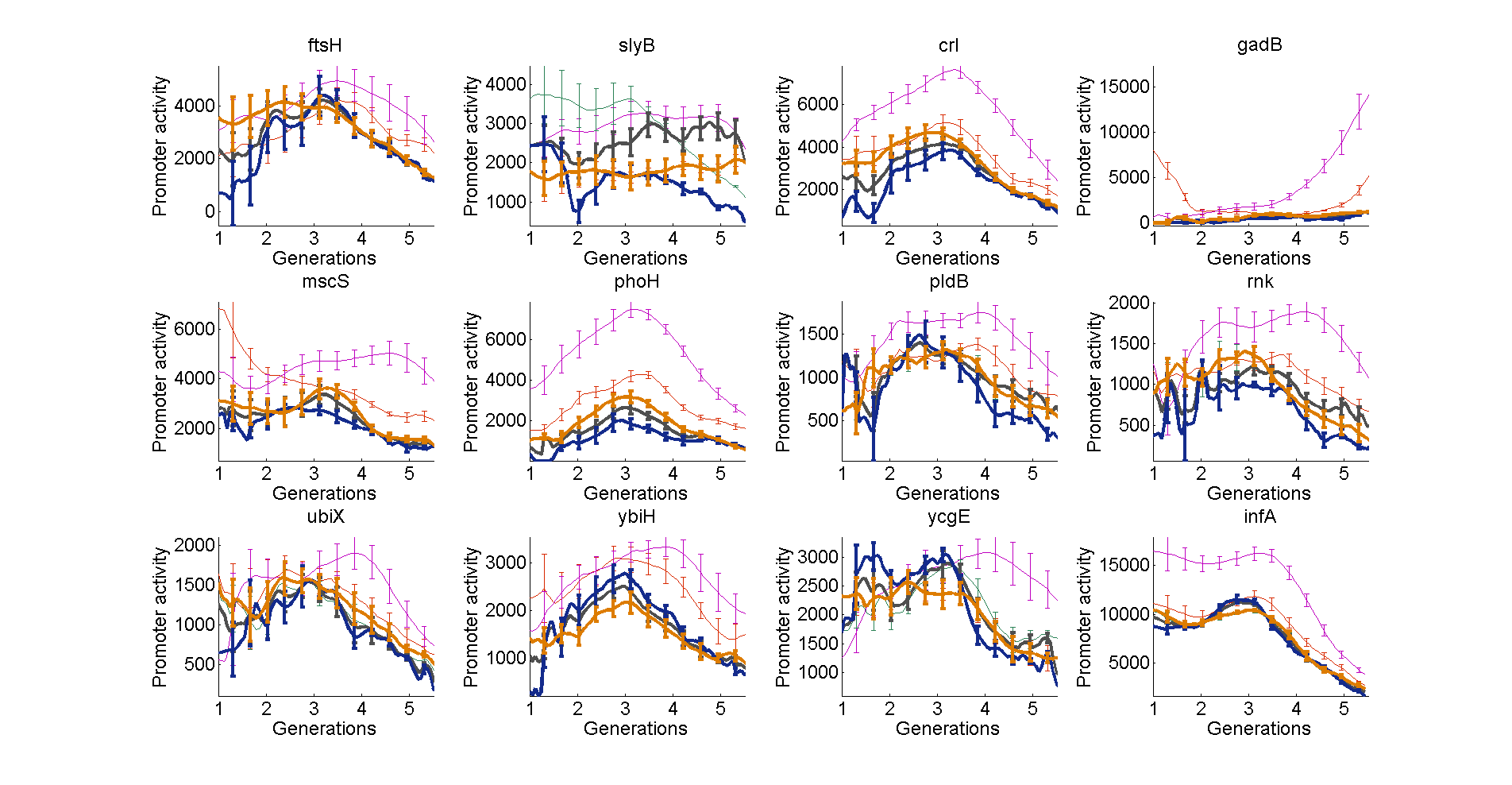


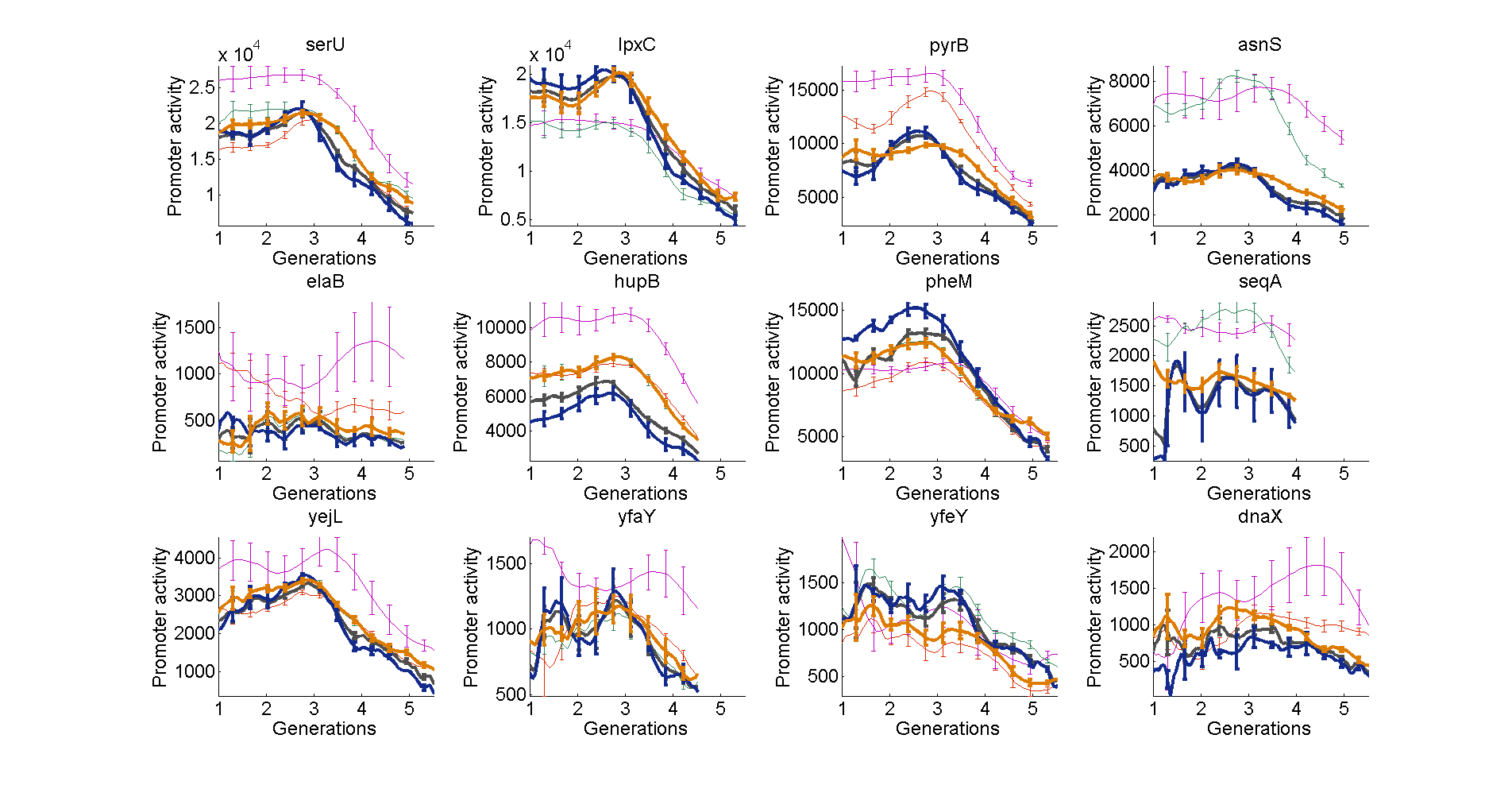


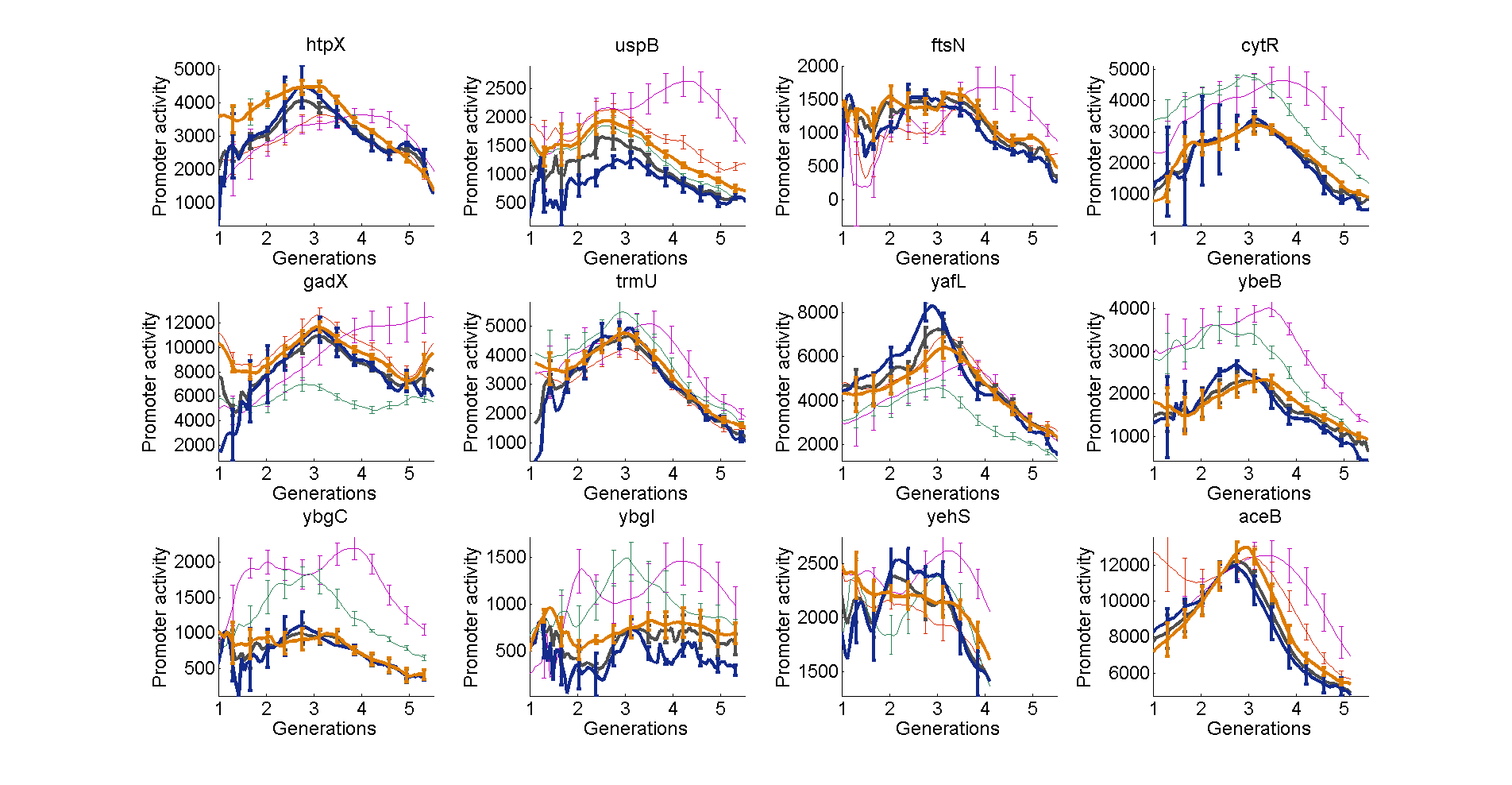


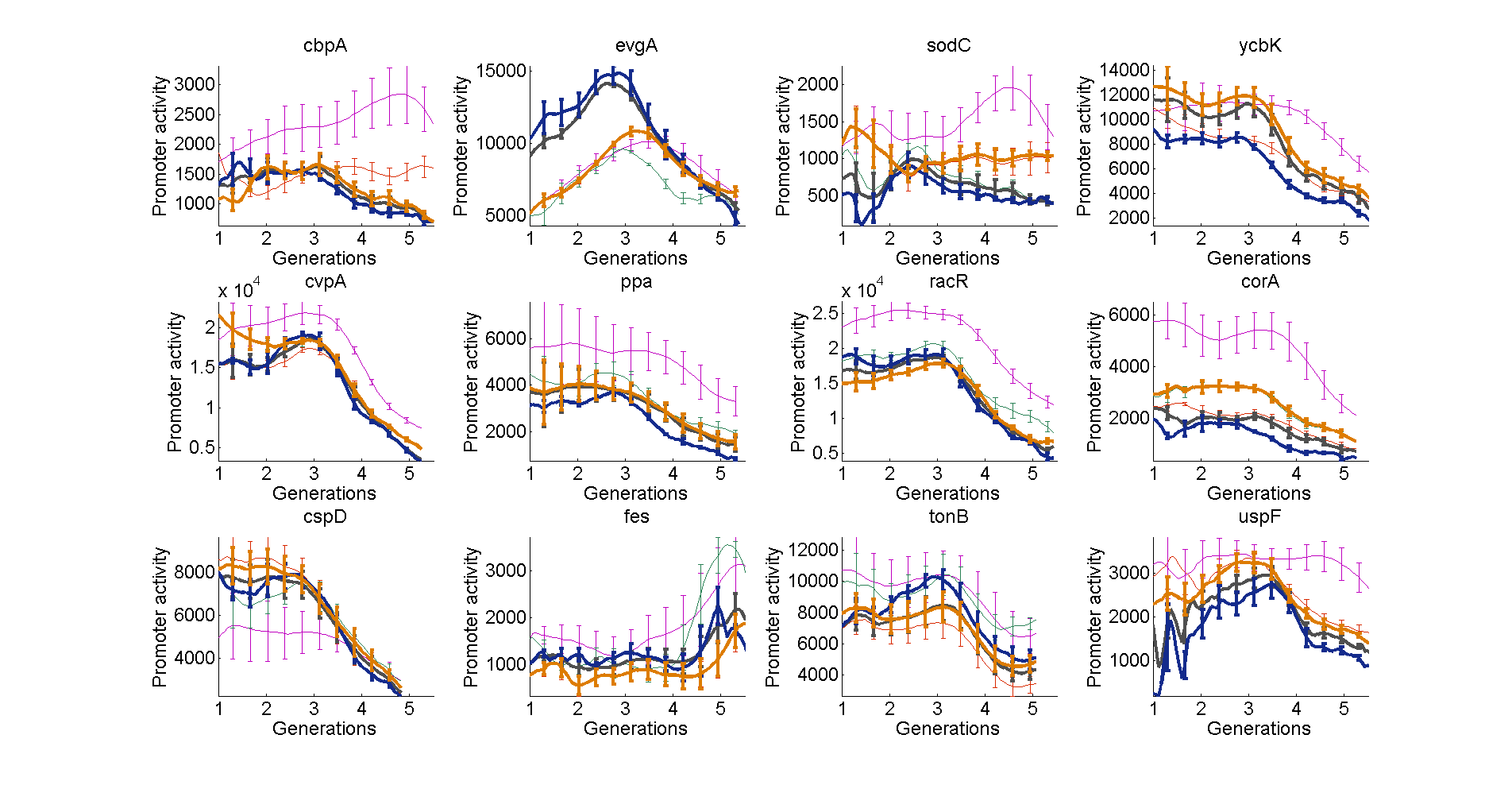


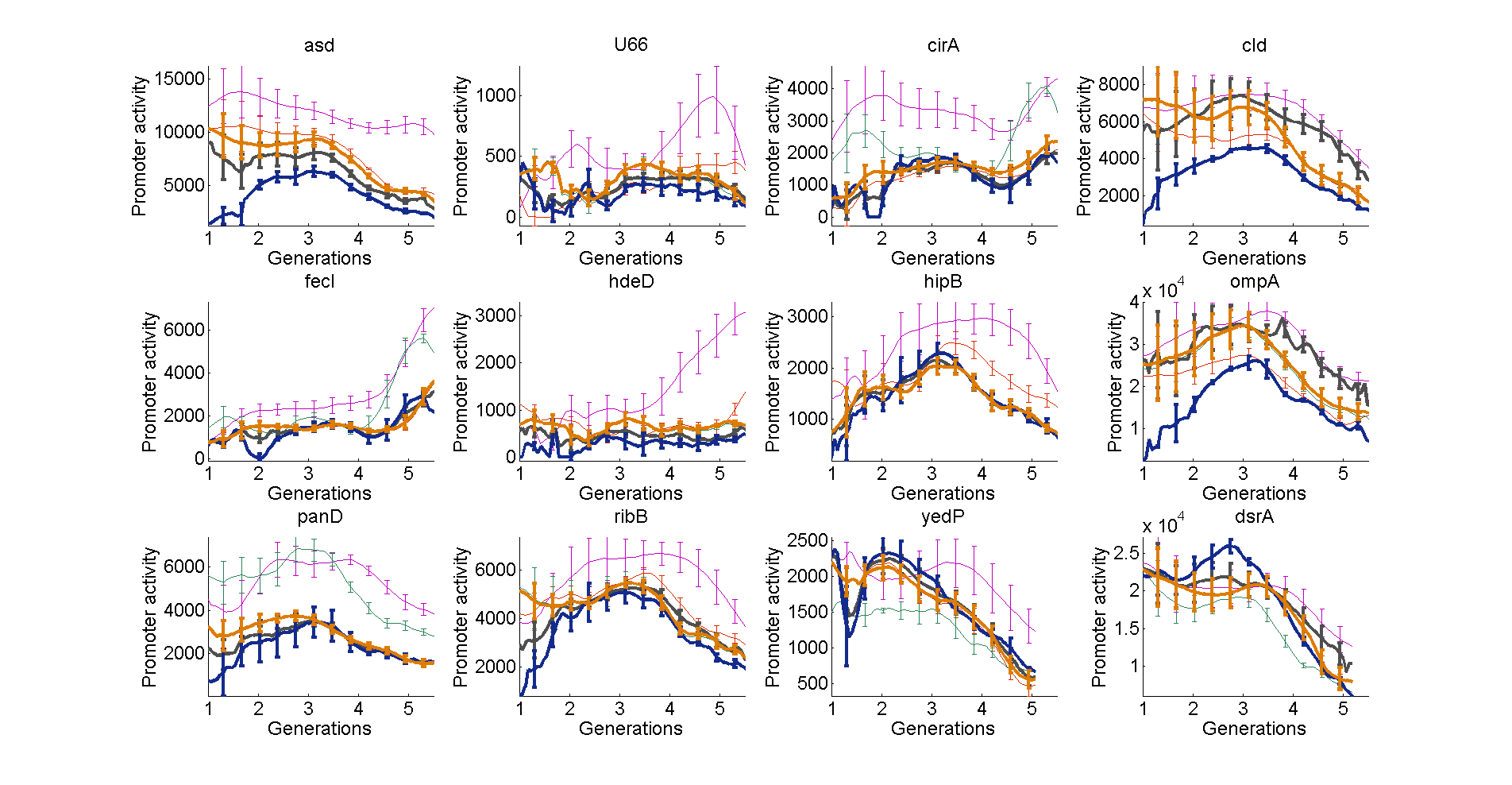


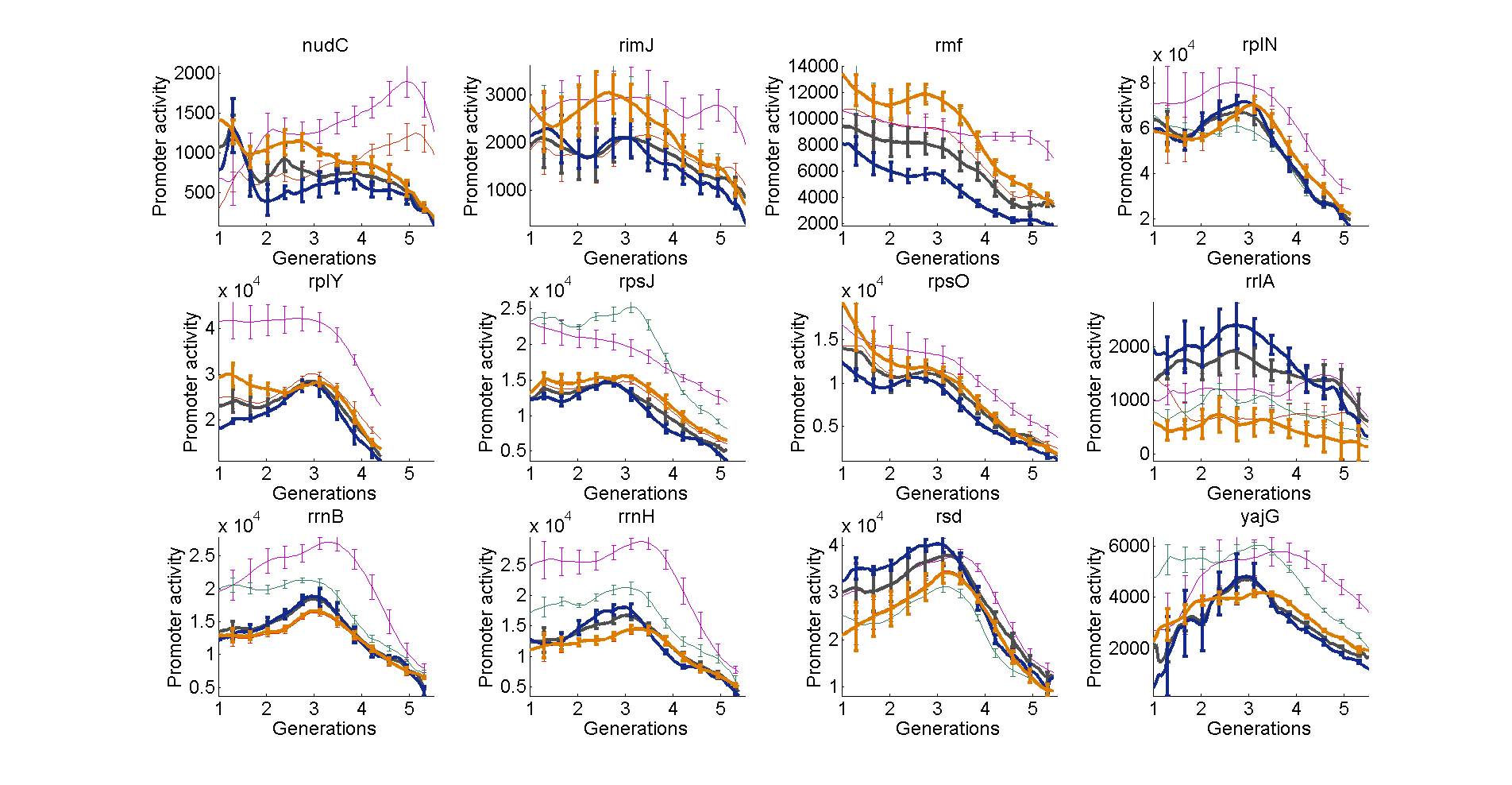


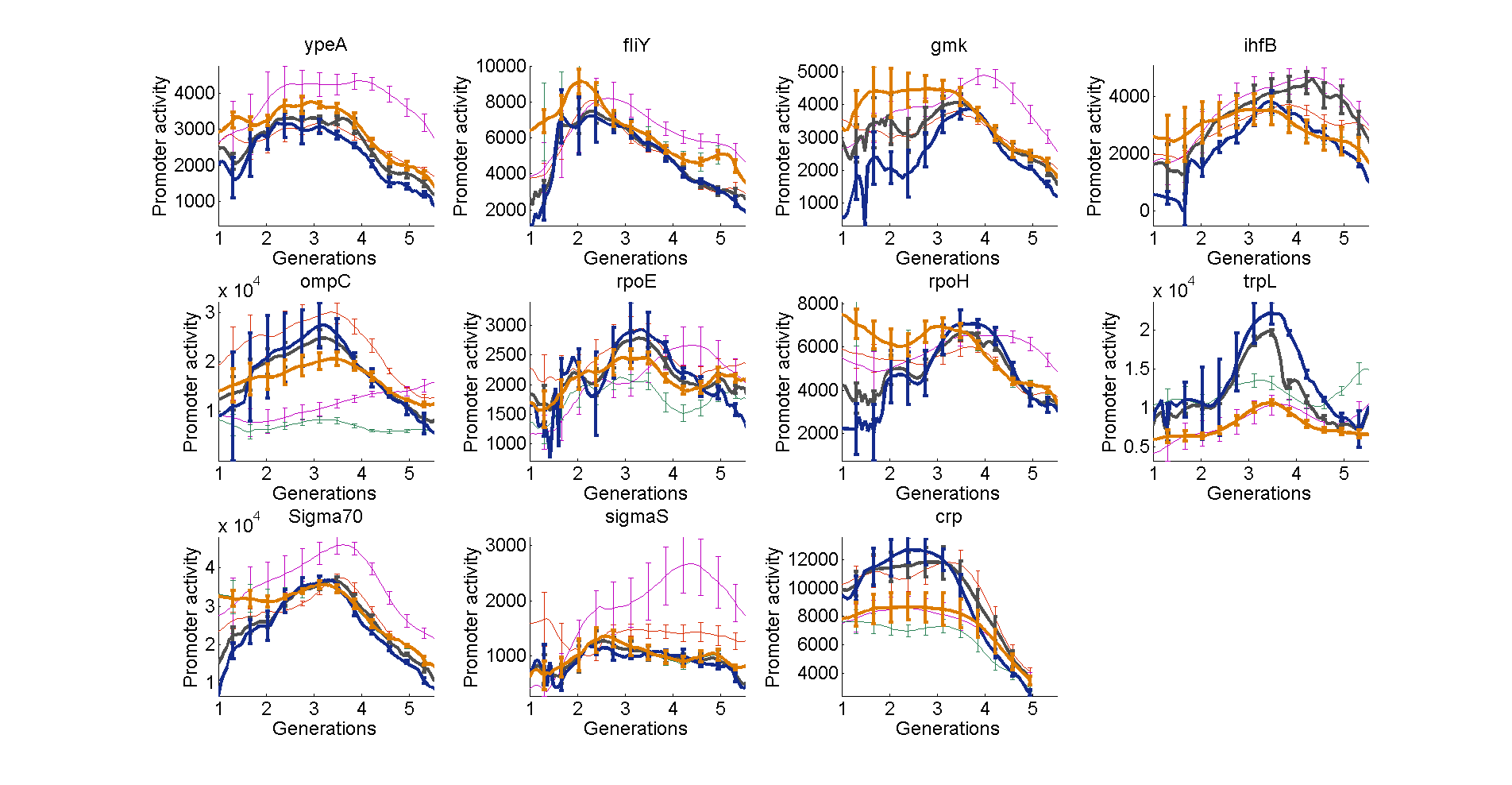


## Casamino acids, Ethanol, H_2_O_2_, Casamino acids + Ethanol + H_2_O_2_

Red – Standard medium + Casamino acids 0.05%

Green – Standard medium + Ethanol 3%

Pink – Standard medium + H_2_O_2_ 10µM

Blue – Standard medium + Casamino acids 0.05% + Ethanol 3% + H_2_O_2_ 10µM

Black – Best fit linear superposition

Orange – Predicion of Casamino acids 0.05% + Ethanol 3% + H_2_O_2_ 10µM


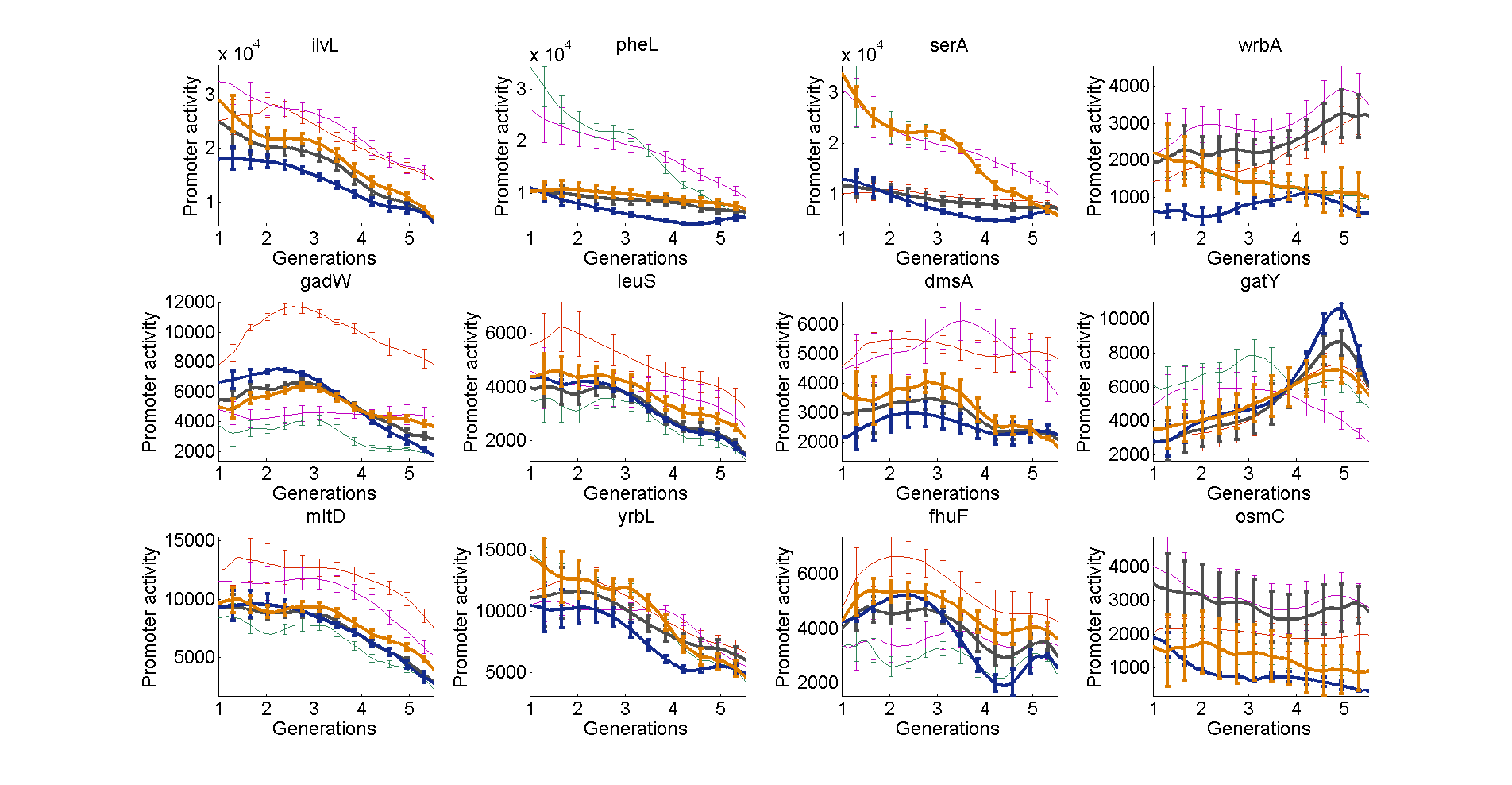


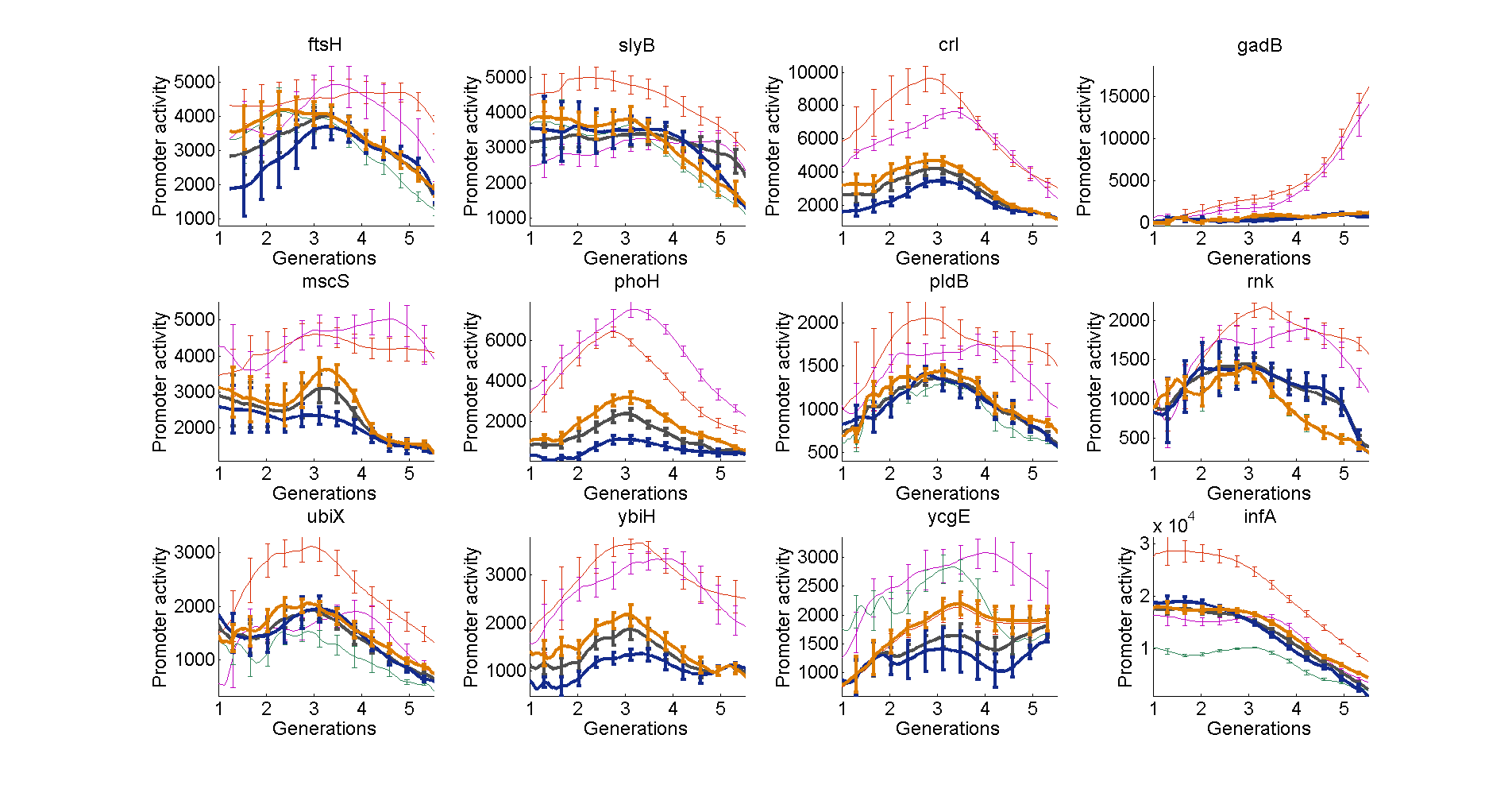


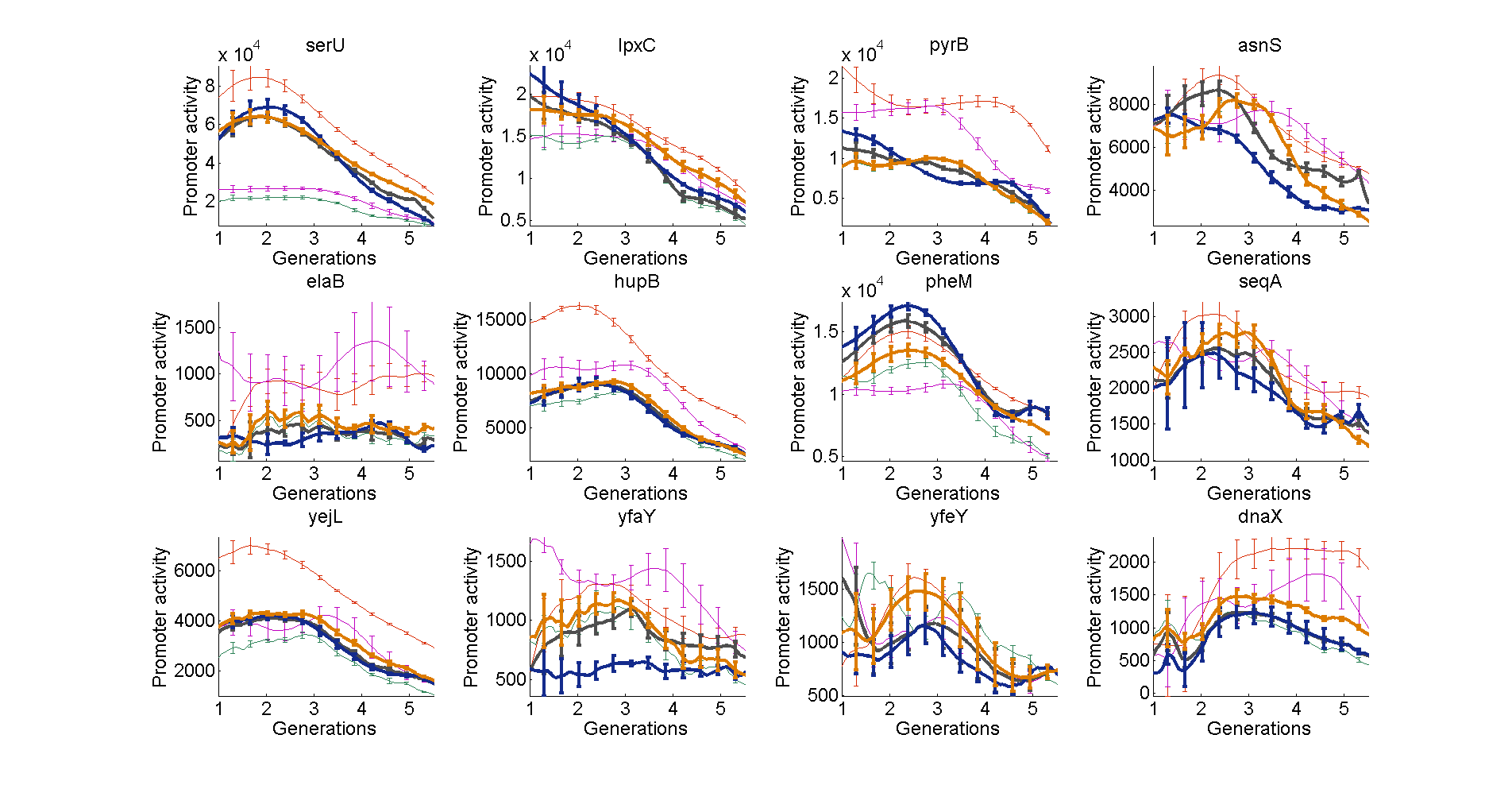


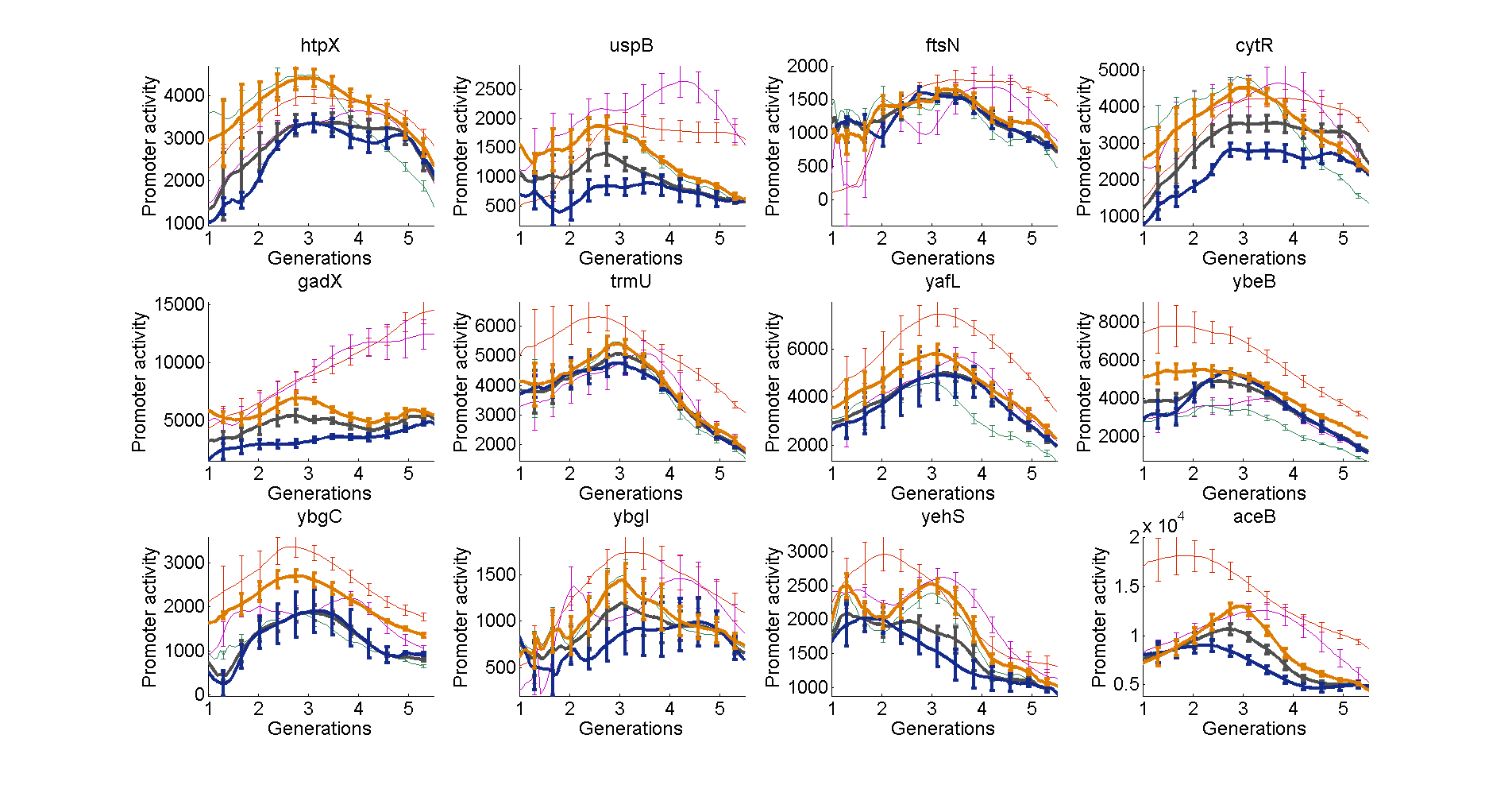


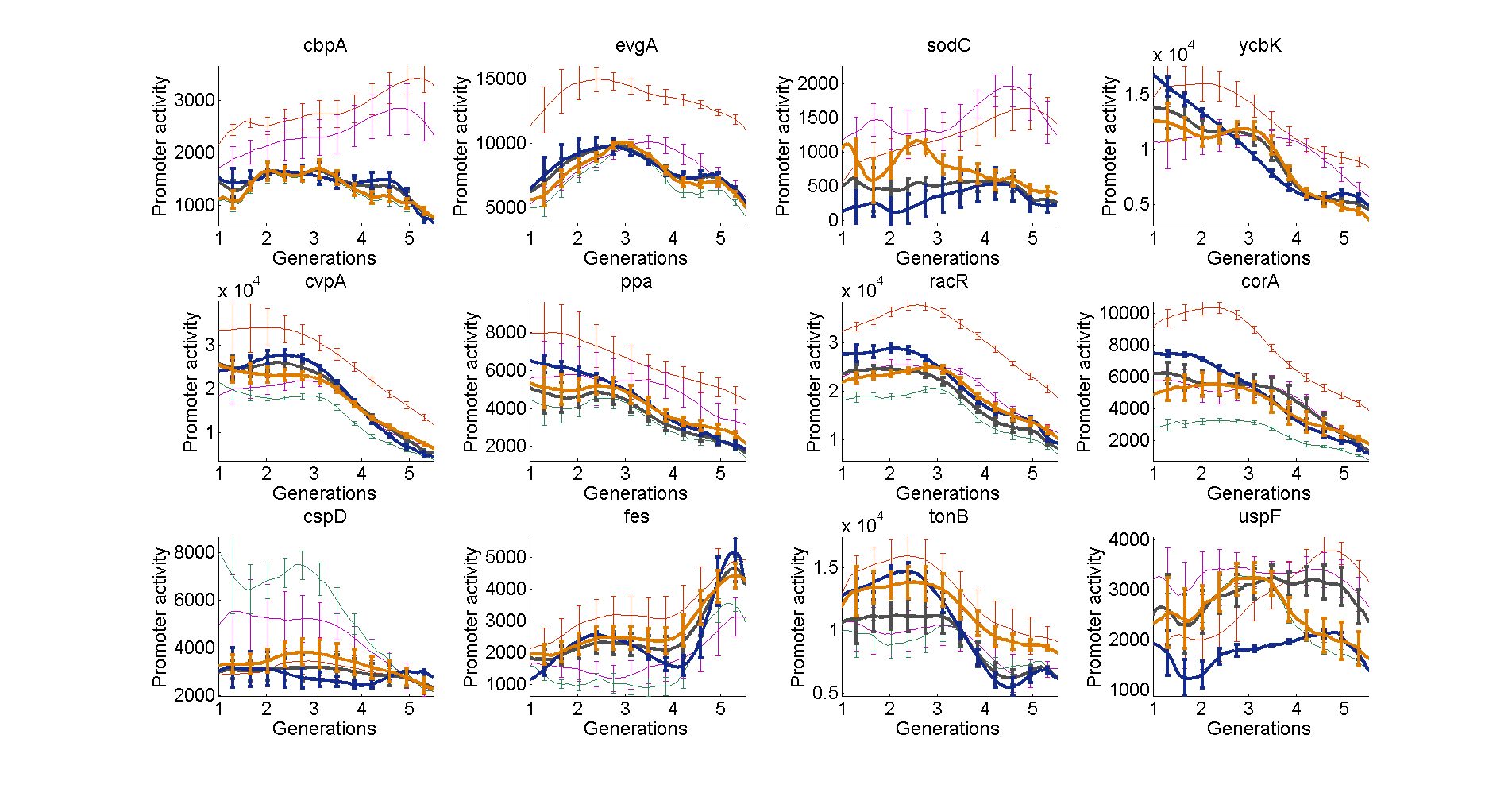


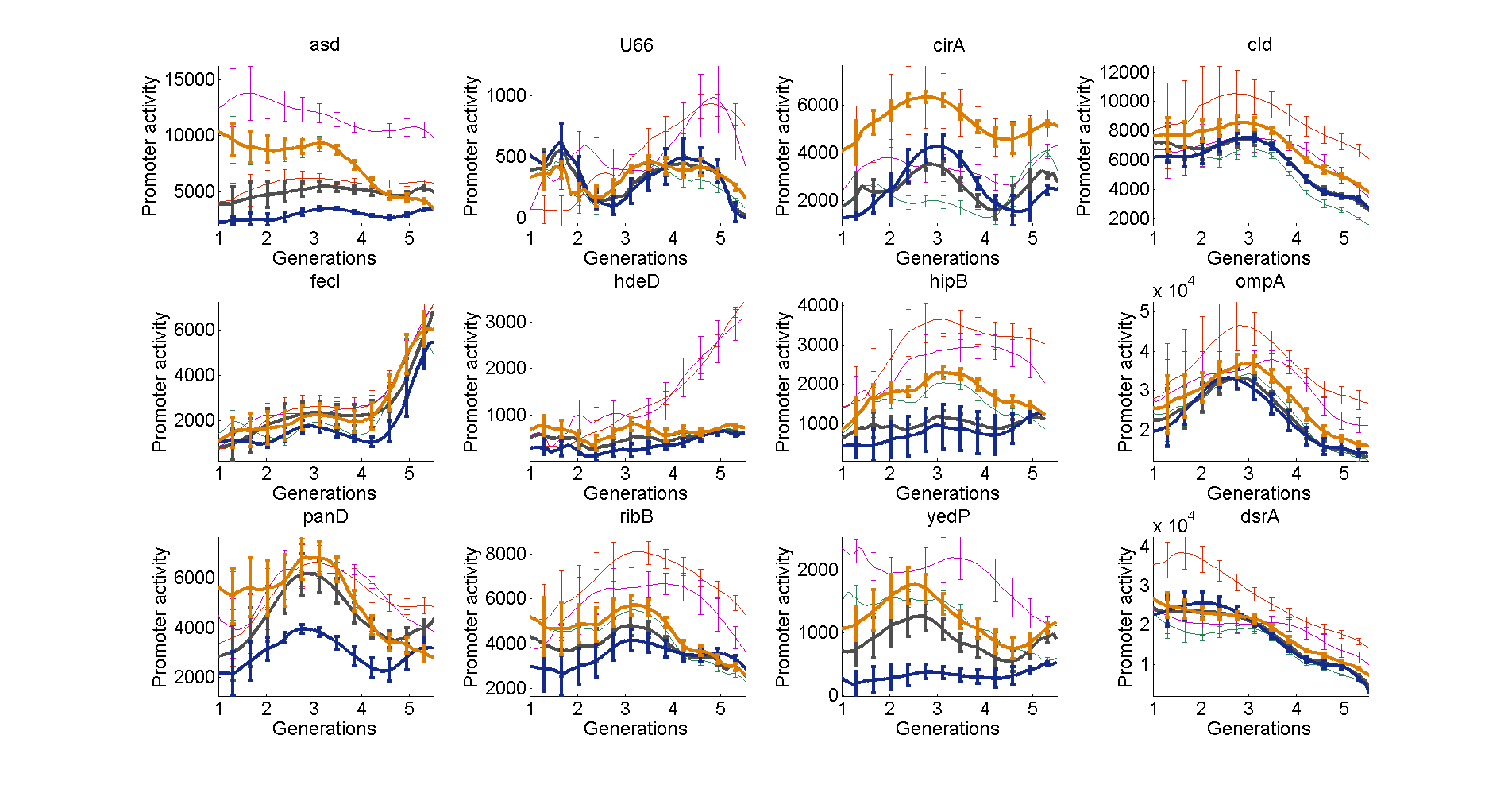


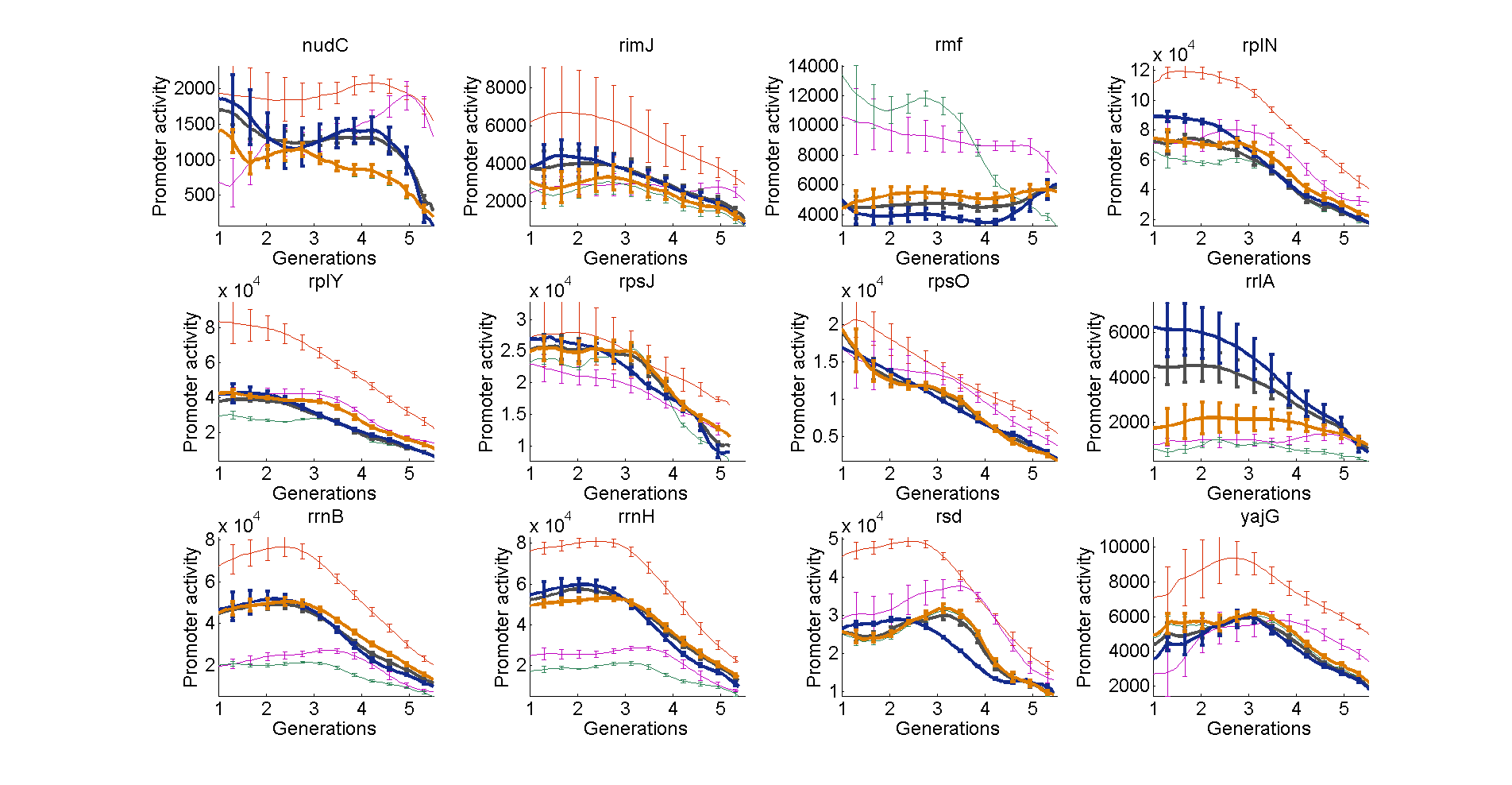


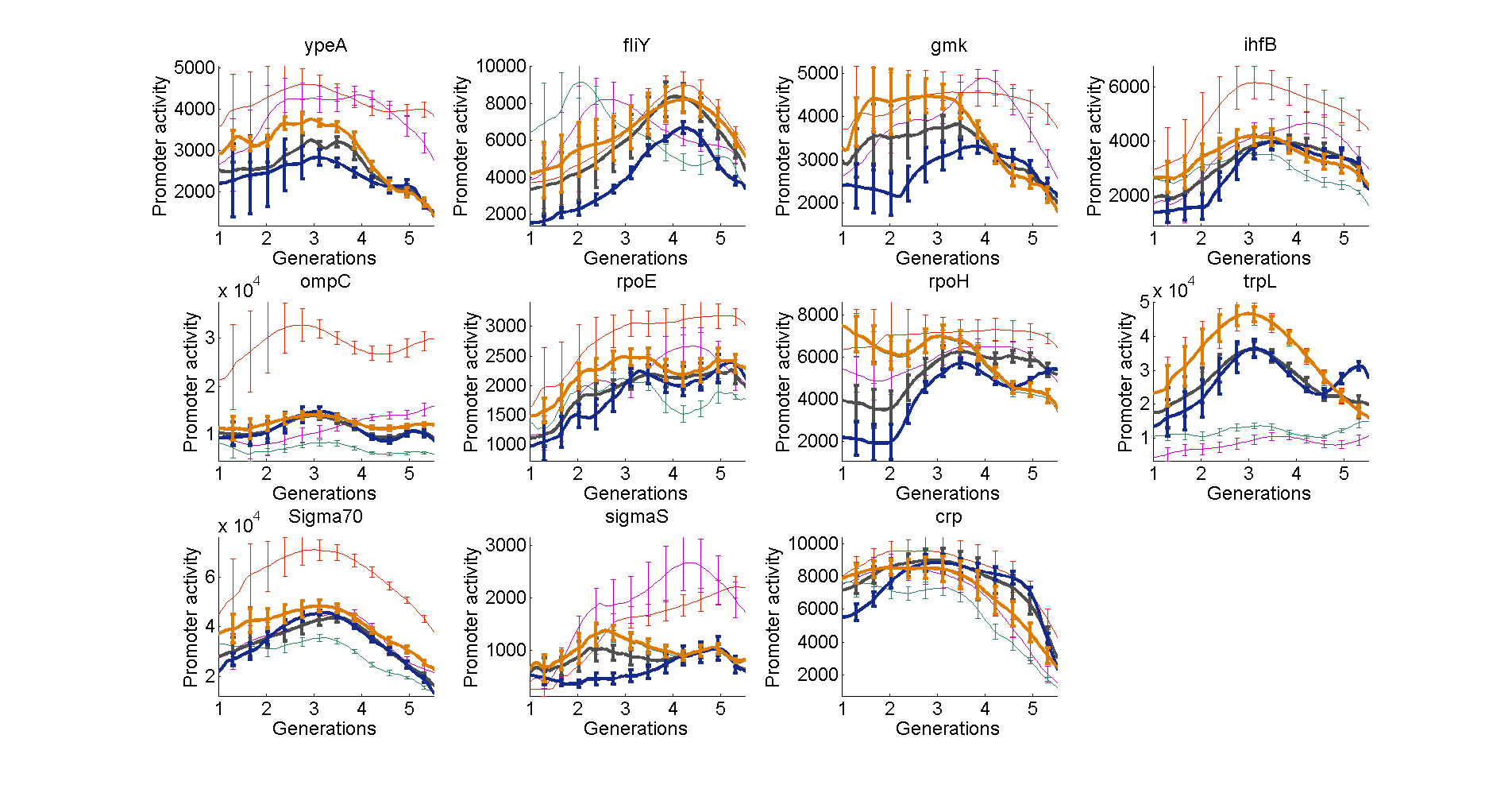


## Casamino acids, Ethanol, H_2_O_2_, NaCl, Casamino acids + Ethanol + H_2_O_2_ + NaCl

Red – Standard medium + Casamino acids 0.05%

Green – Standard medium + Ethanol 3%

Pink – Standard medium + H_2_O_2_ 10µM

Purple – Standard medium + NaCl 300mM

Blue – Standard medium + Casamino acids 0.05% + Ethanol 3% + H_2_O_2_ 10µM + NaCl 300mM

Black – Best fit linear superposition

Orange – Predicion of Casamino acids 0.05% + Ethanol 3% + H_2_O_2_ 10µM + NaCl 300mM


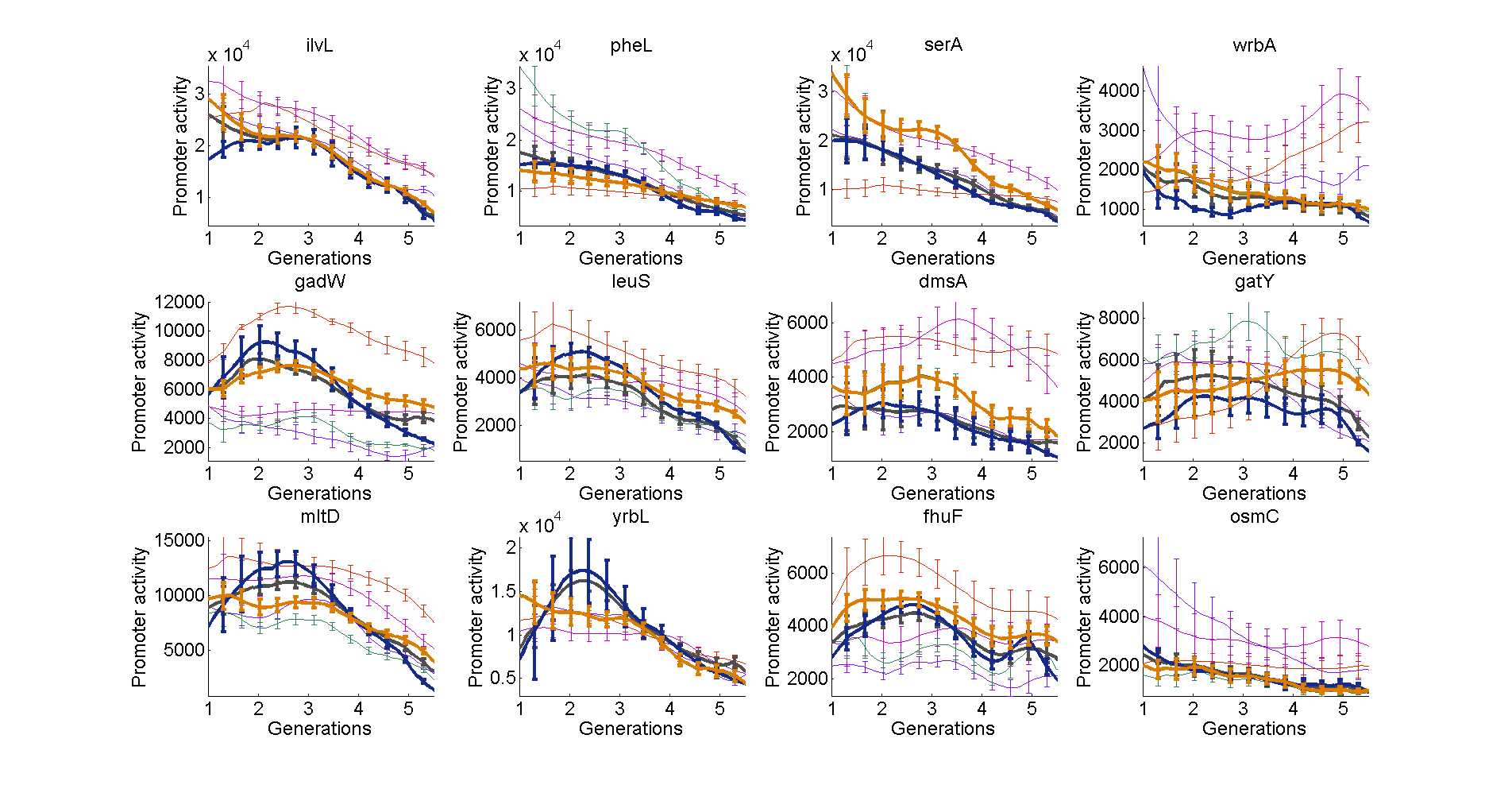


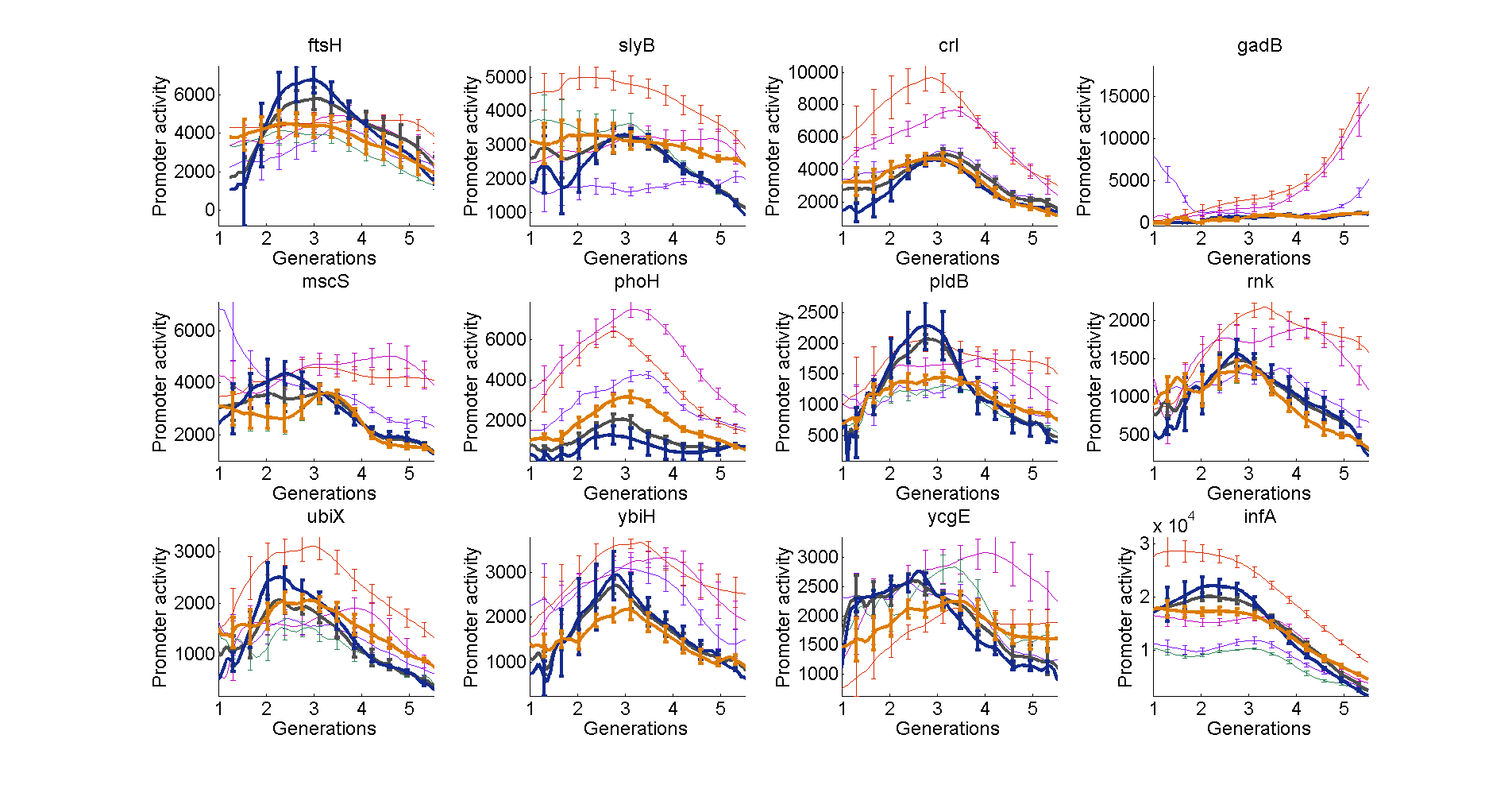


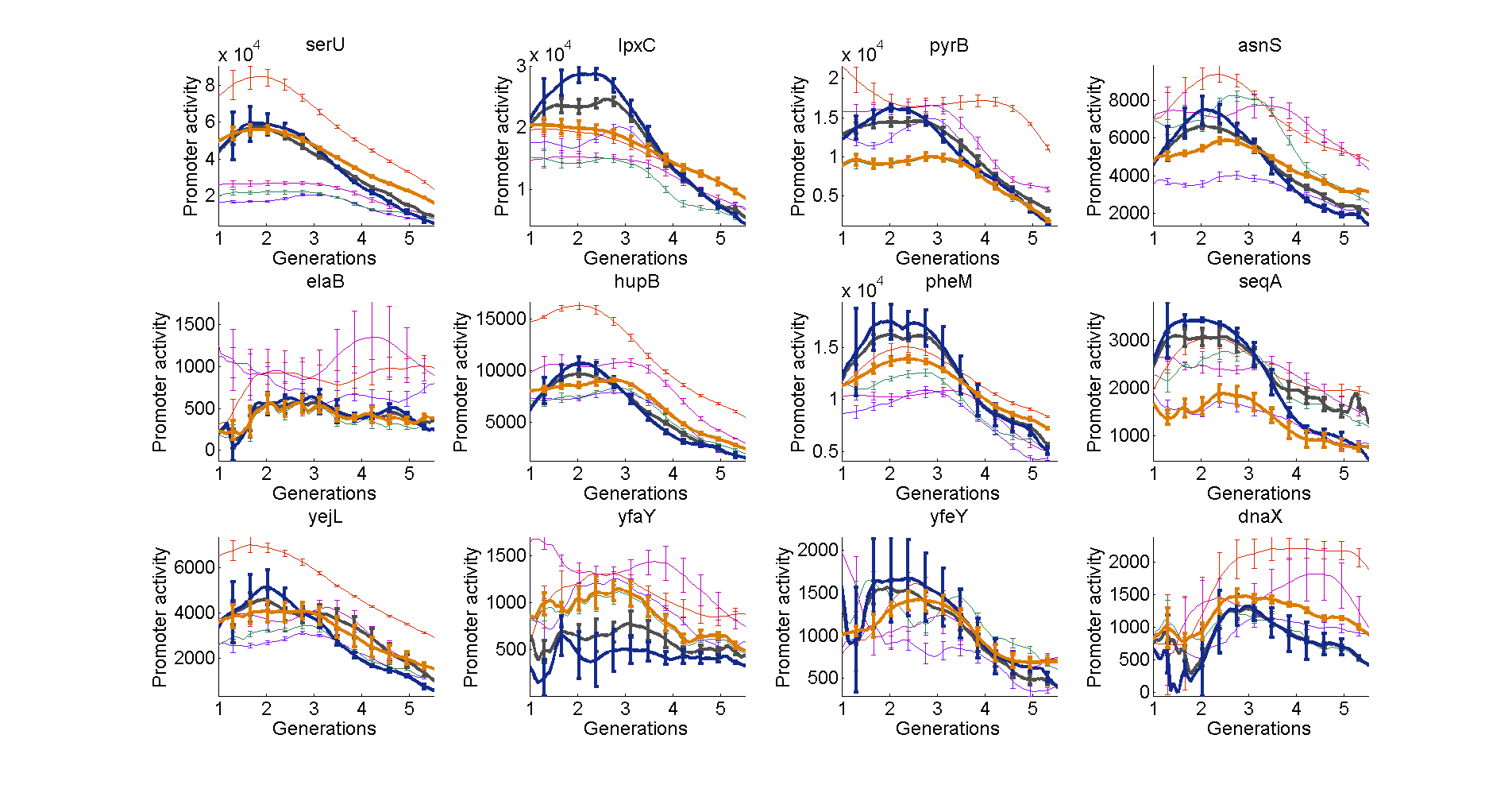


## Glucose, Lactose, Glucose + Lactose

Red – M9 only (not the standard medium) + Glucose 0.03%

Green – M9 only (not the standard medium) + Lactose 0.4%

Blue – M9 only (not the standard medium) + Glucose 0.03% + Lactose 0.4%

Black – best fit linear combination

# References

1. Markovsky I, Huffel SV (2004) A Matlab toolbox for weighted total least squares approximation. Dept. EE, K.U.Leuven. Available: ftp://ftp.esat.kuleuven.be/pub/SISTA/markovsky/reports/04-220.ps.gz.

2. Akaike H (1974) A new look at the statistical model identification. IEEE Trans Autom Control 19: 716–723. doi:10.1109/TAC.1974.1100705.

3. Geva-Zatorsky N, Dekel E, Cohen AA, Danon T, Cohen L, et al. (2010) Protein Dynamics in Drug Combinations: a Linear Superposition of Individual-Drug Responses. Cell 140: 643–651. doi:10.1016/j.cell.2010.02.011.
